# Multi‐Fold Fan‐Shape Surface State Induced by an Isolated Weyl Phonon Beyond No‐Go Theorem

**DOI:** 10.1002/advs.202207508

**Published:** 2023-04-23

**Authors:** Hua‐Hua Fu, Qing‐Bo Liu, Zhe‐Qi Wang, Xiang‐Feng Yang

**Affiliations:** ^1^ School of Physics and Wuhan National High Magnetic Field Center Huazhong University of Science and Technology Wuhan 430074 P. R. China; ^2^ Institute for Quantum Science and Engineering Huazhong University of Science and Technology Wuhan 430074 P. R. China

**Keywords:** density functional theory calculations, isolated Weyl points, topological materials, topological phonons, Weyl nodal walls

## Abstract

Absence of any surface arc state has been regarded as the fundamental property of singular Weyl points, because they are circumvented from the Nielsen‐Ninomiya no‐go theorem. In this work, through systematic investigations on topological properties of isolated Weyl phonons (IWPs) surrounded by closed Weyl nodal walls (WNWs), which are located at the Brillouin zone (BZ) boundaries of bosonic systems, it uncovers that a new kind of phononic surface state, that is, the multi‐fold fan‐shape surface state named by us, is exhibited to connect the projections of IWP and WNWs. Importantly, the number of fan leaves in this surface state is associated with the Chern number of IWP. Moreover, the topological features of charge‐two IWP in K_2_Mg_2_O_3_ (SG No. 96) and charge‐four IWP in Nb_3_Al_2_N (SG No. 213) confirm further the above fundamental properties of this kind of surface state. The theoretical work not only provides an effective way to seek for IWPs as well as to determine their Chern number in real materials, but also uncovers a new class of surface states in the topological Weyl complex composed of IWPs and WNWs.

## Graphical abstract

We uncover that a new kind of phononic surface state, that is, the multi‐fold fan‐shape surface state named by us, is exhibited to connect the projections of isolated Weyl point (IWP) and the surrounded Weyl nodal walls. Importantly, the number of fan leaves in this surface state is associated with the Chern number of IWP.

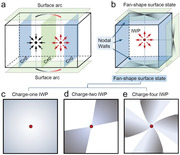

## Introduction

**Figure 1:**
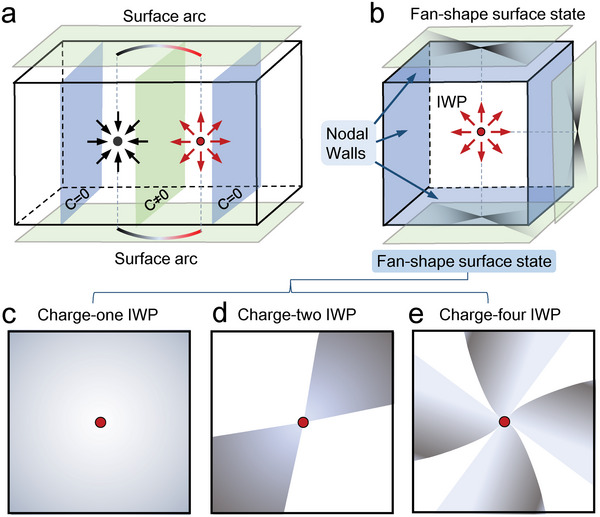
Multi‐fold fan‐shape surface state induced by IWP. a) A conventional Weyl semimetal respecting the no‐go theorem and exhibiting surface arc state connecting the projections of bulk WPs with opposite chirality on the surface BZ. b) Schematic drawing of a phononic crystal with an IWP in the BZ center enclosed by the topologically charged WNWs on the BZ boundaries. Fan‐shape surface state rather than surface arc state exists to connect the projections of IWP and WNWs. c) The one‐fold fan‐shape surface state induced by Charge‐one IWP, here “one‐fold” indicates only one fan leaf to cover the whole surface BZ. d,e) Two‐fold and four‐fold fan‐shape surface states induced by charge‐two IWP and charge‐four IWP, respectively.

Weyl points (WPs) have long been regarded as one class of important elementary particles, because they not only preserve the Lorentz symmetry, but also can be hybridized to form a Dirac point, owing to the paired WPs with opposite chirality.^[^ ^1^, ^2^ ^]^ It is recognized that WPs can be divided into two categories, that is, conventional and unconventional WPs.^[^ ^3^, ^4^, ^5^, ^6^, ^7^, ^8^, ^9^, ^10^ ^]^. The former are contributed by two degenerated bands with a monopole charge of ±1 and need the translation symmetry protection in materials; while the latter by a quantized monopole charge equal to or larger than one,^[^ ^11^ ^]^ and the protections from additional symmetries, such as *C*_31_, *S*_4_ and others,^[^ ^12^ ^]^ are required. As one of their common characteristics, WPs should appear in pairs and the compensation effect must be satisfied in all Weyl systems according to the famous Nielsen‐Ninomiya no‐go theorem.^[^ ^13^, ^14^ ^]^ Following this rule, the total topological charge of all WPs in a topological system should be zero, and the produced surface arc state, as illustrated in **Figure** **1**a, should be exhibited in surface Brillouin zone (BZ) to connect the projections of WPs with opposite chirality.

On the other side, searching for new kind of Weyl phonons has been considered as another central topic in phononic crystals.^[^ ^15^, ^16^, ^17^, ^18^, ^19^, ^20^, ^21^, ^22^, ^23^, ^24^, ^25^, ^26^, ^27^, ^28^, ^29^, ^30^, ^31^, ^32^, ^33^, ^34^ ^]^ Up to date, various Weyl phonons, including single Weyl phonons with Chern number C=±1,^[^ ^35^ ^]^ charge‐two Weyl phonons with C=±2^[^ ^36^ ^]^ and charge‐four Weyl phonons with C=±4,^[^ ^37^ ^]^ have already been proposed to exist in real materials. In particular, spin‐1 and charge‐2 Weyl phonons have been observed in experiments.^[^ ^38^, ^39^ ^]^ In them, the complex charges are still restrained by the no‐go theorem and thus, the double‐helicoid surface state and large surface arcs are exhibited. Moreover, a charge‐four Weyl phonon has been proposed to compensate with four single Weyl phonons in BiIrSe and exhibit interesting quadruple‐helicoid surface arcs.^[^ ^37^ ^]^ Hence, the phononic surface arc state has also been considered as the fundamental property of Weyl phonons due to the constraint from no‐go theorem. Apart from these zero‐dimensional (0D) Weyl phonons, 1D Weyl nodal‐line phonons^[^ ^40^, ^41^, ^42^, ^43^ ^]^ and 2D Weyl nodal‐surface phonons^[^ ^44^, ^45^ ^]^ have already been proposed recently, which provide new material plateaus and crystal structures to understand the conventional no‐go theorem. To explore new kind of Weyl phonons, isolated Weyl phonons (IWPs), which are circumvented from the no‐go theorem, open up a new door. If IWPs exist in real materials, some puzzling issues arise naturally. According to previous studies on monopole WPs, surface arcs disappear from the systems.^[^ ^46^, ^47^ ^]^ However, this conclusion does not deny the possibility of other kind of surface state induced by IWPs. That is to inquiry whether are there other kind of surface state which can be regarded as the hallmark of IWPs? Moreover, how to understand fully the compensation effect on IWPs to ensure zero total charge in a phononic crystal?

In this work, by checking the symmetries in 230 space groups (SGs), we first exhaust the all IWPs existing in the 3D Brillouin zone (BZ) of bosonic systems, and enumerate them at the high‐symmetry points of SGs in **Table** **1**. Due to the unpaired feature, no any surface arc state appears. However, there still exist other kind of surface state with unique geometric configurations. Due to the fact that IWPs are absorbed by the surrounded 2D Weyl nodal walls (WNWs) as drawn in Figure 1b, the surface states are diverged from IWPs to connect WNWs, and further to form multi‐fold fan‐shape surface states. Interestingly, the number of fan leaves in them is tightly associated with the Chern number of IWPs. Considering the all possible screw rotation symmetries in real materials, the charge‐one, charge‐two and charge‐four IWPs may exist as illustrated in Figure 1c–e. It should be stressed that it is the first time to uncover this peculiar phononic surface state, which provides an effective way to check IWPs and identify their Chern numbers. To confirm our findings, we study the topological phonons in two real materials, that is, K_2_Mg_2_O_3_ in SG 96 and Nb_3_Al_2_N in SG 213, and uncover that the charge‐two IWP with C=−2 and the charge‐four IWP with C=+4 exist in them. More importantly, the multi‐fold fan‐shape surface state is exhibited distinctly. Our theoretical work not only provides material platforms to the IWPs beyond no‐go theorem, but also uncovers a new class of surface state in the topological complex composed of IWP and WNWs.

**Table 1:** The completed list of IWPs in 230 SGs. From the first to the seventh column, the related SG number, SG symbol, high‐symmetry point, point groups (PGs), Chern numbers (CNs), Irreps and Generators are denoted

SG No.	SG Symbol	*k*‐point	PGs	CNs	Irreps	Generators
92	*P*4_1_2_1_2	Γ	*D*_4_	±2	Γ_5_	$\left\{C_{4z}^{+}|00\frac{1}{4}\right\}\;\left\{C_{2x}|\frac{1}{2}\frac{1}{2}0\right\}$, T
96	*P*4_3_2_1_2	Γ	*D*_4_	±2	Γ_5_	$\left\{C_{4z}^{+}|00\frac{3}{4}\right\}\;\left\{C_{2x}|\frac{1}{2}\frac{1}{2}0\right\}$, T
198	*P*2_1_3	Γ	*T*	±4	Γ_2_Γ_3_	$\left\{C_{31}^{+}|000\right\}\;\left\{C_{2z}|\frac{1}{2}0\frac{1}{2}\right\}\;\left\{C_{2y}|0\frac{1}{2}\frac{1}{2}\right\}$, T
212	*P*4_3_32	Γ	*O*	±4	Γ_3_	$\left\{C_{31}^{-}|000\right\}\;\left\{C_{2z}|\frac{1}{2}0\frac{1}{2}\right\}\;\left\{C_{2x}|\frac{1}{2}\frac{1}{2}0\right\}\;\left\{C_{2a}|\frac{1}{4}\frac{3}{4}\frac{3}{4}\right\},T$
213	*P*4_1_32	Γ	*O*	±4	Γ_3_	$\left\{C_{31}^{-}|000\right\}\;\left\{C_{2z}|\frac{1}{2}0\frac{1}{2}\right\}\;\left\{C_{2x}|\frac{1}{2}\frac{1}{2}0\right\}\;\left\{C_{2a}|\frac{3}{4}\frac{1}{4}\frac{1}{4}\right\}$, T

## Results and Discussion

To seek for the IWPs with various charge numbers, we should first find out the litter groups (LGs) having the irreducible representations (irreps) with the corresponding dimensions in the presence of time‐reversal symmetry (T). Note that for any IWP, our searching covers the all 230 SGs, and the lattice symmetries of materials are located at the high‐symmetry points in 3D BZ. Second, we exclude the SGs that contain the space‐inversion symmetry (P), mirror and improper rotational symmetries to ensure that no any nodal line exists at these points. Third, we need to find the SGs possessing three one‐fold, two‐fold or four‐fold screw axial symmetries along three axis directions, which may form three nodal surfaces at *k*_*x*_, *k*_*y*_ and *k*_*z*_ = ±π planes.^[^ ^48^ ^]^ Finally, we screen out the related multi‐fold points at high‐symmetry points, and then calculate their charge numbers one by one to gain the IWPs hosting the charges we need (e.g., ±2 or ±4). Following this process, the all two‐fold and four‐fold Weyl phonons meeting the requirements of IWPs are obtained as listed in Table 1. Furthermore, through symmetry analysis based on a two‐band *k* · *p* model Hamiltonian of IWPs with C=±2 at the point Γ in SGs 96 in Table 1, we find that the low‐energy phononic dispersion of charge‐two IWPs displays a *k*‐type feature along the [001] direction, while a *k* ^2^‐type feature along other directions in phononic bands. Meanwhile, from the four‐band *k* · *p* model Hamiltonian of IWPs with C=±4 at the point Γ in SGs 213 in Table 1, we conclude that the low‐energy phononic band of charge‐four IWP displays a *k* ^3^‐type feature along the [111] direction, while a *k* ^2^‐type feature along other directions. These peculiar features of low‐energy dispersions give the fundamental properties to determine charge‐two and charge‐four IWPs in real materials.

**Figure 2:**
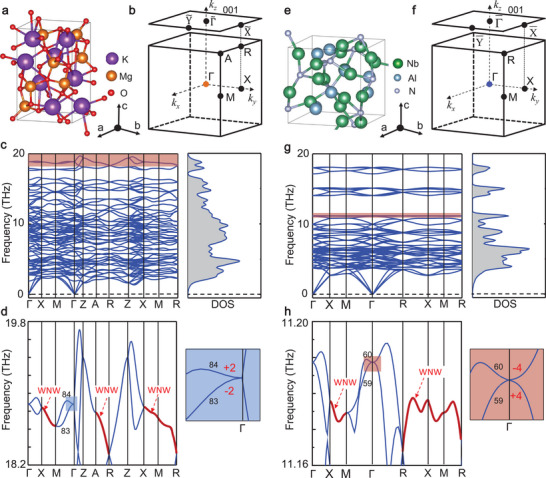
Realistic materials to realize charge‐two and charge‐four IWPs. a) Crystal structure of K_2_Mg_2_O_3_ in SG 96 in a primitive cell. b) The bulk BZ of K_2_Mg_2_O_3_ and the (001) surface BZ. c) The phonon spectra of K_2_Mg_2_O_3_ along the high‐symmetry paths and the corresponding phononic density of states (DOS). d) An enlarged image of phonon dispersions of a light‐red box highlighted in c, where the nontrivial bands (red lines) to construct WNWs and two nontrivial bands with C=±2 to construct the charge‐two IWP at the point Γ (right panel) are also given. e) Crystal structure of Nb_3_Al_2_N in SG 213 in a primitive cell. f) Bulk BZ of Nb_3_Al_2_N and the (001) surface BZ. g) Phononic spectra and the phononic DOS of Nb_3_Al_2_N. h) An enlarged image of phonon dispersions of a light‐red box highlighted in g, where the nontrivial bands (red lines) to construct WNWs and two nontrivial bands with C=±4 to construct the charge‐four IWP at the point Γ (right panel) are also highlighted.

To verify the existence of topological complex composed by charge‐two (or charge‐four) IWPs and the surrounded WNWs, and to further explore their topologically nontrivial features, we study the phononic structures of two real material samples, that is, K_2_Mg_2_O_3_ in SG 96 (*P*4_3_2_1_2) and Nb_3_Al_2_N in SG 213 (*P*4_1_32). We obtained their crystallographic data from Ref.^[^ ^49^ ^]^ and plotted their primitive cells in **Figure** **2a,e**, respectively. The primitive cell of K_2_Mg_2_O_3_ contains twenty‐eight atoms with eight K, and eight Mg and twelve O atoms, and the primitive cell of Nb_3_Al_2_N contains twenty‐four atoms with twelve Nb, eight Al and four N atoms. Moreover, the first 3D BZs of these two material samples are drawn in Figure 2b,f, respectively, and the top black square denotes the (001) surface BZ.

**Figure 3:**
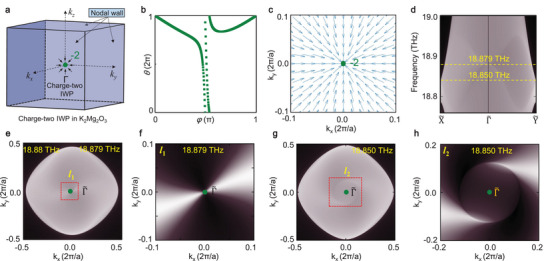
Topological features of charge‐two IWP and the related surface states in K_2_Mg_2_O_3_. a) Spatial distributions of charge‐two IWP and the related WNWs in the first BZ. b) Evolutions of Wannier centers of the 83^*th*^ phononic band in K_2_Mg_2_O_3_. c) Spatial distributions of Berry curvature in the (001) surface BZ. d) Surface states in the (001) plane. e,g) Isofrequency surface contours at the frequency value 18.879 and 18.850 THz, respectively, where the square regimes *l*_1_ and *l*_2_ are chosen around the projection $\tilde{\Gamma }$ of high‐symmetry point Γ. f,h) Isofrequency surface contours along the square paths *l*_1_ and *l*_2_, respectively.

As listed in Table‐I, two classes of IWPs, that is, charge‐two and charge‐four IWPs together with the corresponding symmetry‐prediction conditions, irreps and generators, are summarized in details. First, we discuss the topological features of charge‐two IWPs and the related surface states. The phononic bands and density of states (DOS) of K_2_Mg_2_O_3_ are drawn in Figure 2c, in which the absence of imaginary frequencies indicates its thermodynamical stability. One can see that two‐band crossings appear at the high‐symmetry point Γ. To demonstrate clearly the topological characters around the IWP, in Figure 2d, we enlarged the phonon dispersions including the 83^*rd*^ and 84^*th*^ bands, which are highlighted by a light‐red box in Figure 2c. We find that the low‐energy parts of the 83^*rd*^ and 84^*th*^ bands at Γ (see right panel in Figure 2d) display *k*‐relation dispersions in the *k*_*z*_ direction while *k* ^2^‐relation dispersions in other directions, in good agreement with the above fundamental properties of charge‐two IWP drawn from *k* · *p* model. Moreover, our further calculations show that these two bands possess C=±2 (see Figure 2d), and the evolutions of Wannier center of the 83^*rd*^ phononic band indicate its nontrivial feature (see **Figure** **3**b). These properties verify that a well‐defined WP appears at the point Γ. More importantly, along the high‐symmetry pathes X‐M, A‐R and X‐M‐R (see red lines highlighted in Figure 2d), the two‐fold nontrivial bands with the Chern numbers C=±2 maintain well, indicating the existence of nodal surface in these surface planes.

The above findings gained from phononic spectra demonstrate that a charge‐two IWP is located at the center of 3D BZ (see the dot colored by turquoise) and the closed WNWs are distributed at all boundaries of 3D BZ as illustrated in Figure 3a. Through examining the charity of this charge‐two IWP, we find that its Chern number is ‐2 (see Figure 3a). Moreover, we are sure that there no any other class of WPs with opposite Chern numbers exist in the first BZ of K_2_Mg_2_O_3_. These properties confirm the unipolarity of charge‐two IWP and demonstrate that the charge‐two IWP is absorbed by the surrounded WNWs at the BZ boundaries. Therefore, the WNWs can be viewed as the counterpart of charge‐two IWP, which establishes a peculiar compensation effect for unipolar WPs. To support this conclusion, the spatial distribution of Berry curvature of charge‐two IWP in the (001) plane is calculated as drawn in Figure 3c. We find that the projection of charge‐two IWP at $\tilde{\Gamma }$ acts as an isolated “sink” point of Berry curvature, and the Berry curvature field of charge‐two IWP is emitted from the surrounded WNWs to the projection of charge‐two IWP, constructing the special topologically nontrivial features of the topological Weyl complex composed of charge‐two IWP and the surrounded WNWs.

The phononic surface states of K_2_Mg_2_O_3_ in the (001) plane are calculated and shown in Figure 3d. We find that a quadratic Dirac cone appears at the point $\tilde{\Gamma }$ and along the high‐symmetry path $\tilde{X}-\tilde{\Gamma }-\tilde{Y}$. To gain more details, the isofrequency surface contours at 18.879 THz are drawn in Figure 3e and in the square regime *l*_1_, the phononic surface state is highlighted in Figure 3f. One can see that there is indeed no any surface arc state to connect the projection of WP, which is much different from the topological features of any paired WPs reported previously. However, it is interesting that another kind of phononic surface states, looking like two symmetrical fan leaves, appear symmetrically around the point $\tilde{\Gamma }$ and spread from this point to the BZ boundaries (see Figure 3f). It should be stressed that these fan‐shape surface state is generated both by charge‐two IWP and by the related nontrivial WNWs, indicating its robustness, which is very similar to other kinds of surface states in topological systems, such as the surface arc produced by Weyl points and the drumbhead‐shape surface state by Weyl nodal lines. Moreover, the number of fan leaves in it is determined by the four‐fold rotation symmetry $\left\{C_{4z}^{+}|00\frac{1}{4}\right\}$ in the *k*_*z*_‐direction, hence it is associated with the Chern number of charge‐two IWP. That is to say the charge number “2” is equal to the number of fan leaves, since they are both associated with the screw rotation symmetry.

It is obvious that the geometrical configurations of fan‐shape surface states uncovered here are much different from other surface states, including the surface arc state and quadruple‐helix surface state reported previously in topological systems. Nevertheless, the formation of fan‐shape surface states can be well understood here. First, as illustrated in some previous literatures,^[^ ^50^, ^51^, ^52^ ^]^, Chern number can still be applied as an effective topological invariant to define the topologically nontrivial nature of WNWs. Although the Chern numbers of some WNWs are very difficult to calculate, we can obtain the charge number of WNWs studied here getting help from the rule that the net topological charge in the first BZ should be zero. Thus, the topological charge of the WNWs in the material sample K_2_Mg_2_O_3_ should be +2, since there only one WP with the charge ‐2 exists in the first BZ. Second, as drawn in Figure 3a, the Berry flux generated from the IWP with the charge ‐2 is absorbed absolutely by the six nodal surfaces at all BZ boundaries. This property basically determines the spatial distributions and geometrical configuration of the related surface state. Moreover, the formation of fan‐shape surface state can been drawn from the evolution of surface arc state drawn in Figure 1a. We may imagine that as the left WP in Figure 1a is decomposed into countless nodal points, which disperse further in all surfaces BZ to construct the gapless WNWs. Whereas, the right WP maintains well to work as an IWP. In this process, the original surface arc state expands naturally to form fan‐shape surface state. This evolution of surface arc state gives us the simplest physical image to grasp the physical characters of fan‐shape surface state. Considering the unique physical properties mentioned above, we regard these fan‐shape surface state as a new kind of surface state in topological systems, since they have not been reported anywhere up to date. To emphasize this finding, we name the surface states drawn in Figure 3f,h two‐fold fan‐shape surface state, and their fundamental properties provide us an effective way to detect charge‐two IWP and its Chern number.

To examine the robustness of fan‐shape phononic surface states uncovered here, we turn to study the surface state in another isofrequency plane with the frequency 18.850 THz (see Figure 3h), which is far from the crossing point of bands 83^*rd*^ and 84^*th*^ as shown in Figure 3d. In this situation, the surface states are partially contributed by trivial bulk states. However, the twofold fan‐shape surface state displays well (see Figure 3h), indicating the robustness of the twofold fan‐shape surface state. Furthermore, considering the same screw rotation symmetries in other vertical axis directions, this kind of surface state should appear in other surfaces BZ. Motivated by this derivation, we studied the surface state in the (100) plane of K_2_Mg_2_O_3_ as provided in Figure S1 (Supporting Information). One may see that the twofold fan‐shape surface state also exists in that plane. The difference in twofold fan‐shape surface states in the (001) and (100) planes reflects the anisotropy in these two vertical directions of K_2_Mg_2_O_3_. Nevertheless, it should be pointed out that this kind of surface states can still be scattered by bulk states. However, due to its robustness, we may distinguish the fan‐shape surface states form bulk states by contrasting the surface and bulk state projections with those of the bare bulk band projections, and further by examining the disparities between them.

**Figure 4:**
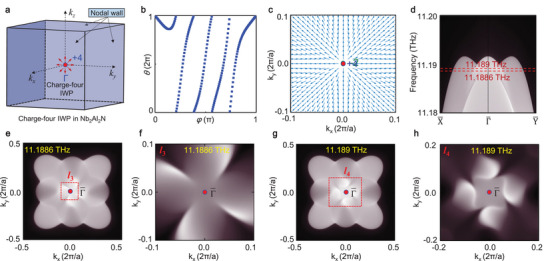
Topological features of charge‐four IWP and the related surface states in Nb_3_Al_2_N. a) Spatial distributions of charge‐four IWP and the related WNWs in the first BZ. b) Evolutions of Wannier centers of the 59^*th*^ phononic band in Nb_3_Al_2_N. c) Spatial distributions of Berry curvature in the (001) surface BZ. d) Surface states in the (001) plane. e,g) Isofrequency surface contours at the frequency value 11.1886 and 11.189 THz, respectively, where the square regimes *l*_3_ and *l*_4_ are chosen around the projection ($\bar{\Gamma }$) of high‐symmetry point Γ. f,h) Isofrequency surface contours along the square regimes *l*_3_ and *l*_4_, respectively.

To demonstrate the universality of multi‐fold fan‐shape surface state induced both by IWP and WNWs, we turn to study the charge‐four IWP in a real material. The phononic dispersion of Nb_3_Al_2_N (SG 213) is given in Figure 2g and meanwhile, the evolutions of Wannier centers of a special band (i.e., the 59^*th*^), the distributions of Berry curvature and the surface state in the (001) plane are drawn in Figure 4b–d. The isofrequency surface states and the related surface states in the square regime *l*_3_ are drawn in Figure 4e,f. One may find that two‐band crossings, constructed by the 59^*th*^ and 60^*th*^ bands, appear at the point Γ. Our further calculations show that this crossing point displays as a distinct WP with the charge +4, as illustrated in Figure 2h, that is, an enlarged image of the light‐red box highlighted in Figure 2g. Moreover, in SG 213, three screw‐rotation symmetries $\left\{C_{2x}|\frac{1}{2}\frac{1}{2}\frac{1}{4}\right\}$, $\left\{C_{2y}|\frac{1}{2}\frac{1}{2}\frac{3}{4}\right\}$ and $\left\{C_{2z}|00\frac{1}{2}\right\}$ exist in the 3D BZ, leading to the appearance of 2D degenerate points or bands at the high‐symmetry points M, R and X, or along the paths X‐M, R‐X, and R‐M, as drawn in Figure 2h by red lines, which also support that the appearance of 2D degenerate points in the surface plane R‐M‐X. Considering further that the four‐fold rotation symmetry in this plane, Weyl nodal surface exists in both surface planes of *k*_*y*_ = ±π. By the same symmetry analysis, Weyl nodal surfaces also exist in other four surface planes at *k*_*x*, *z*_ = ±π. As a result, the nontrivial WNWs exist at the 3D BZ boundaries of Nb_3_Al_2_N.

Similar to the charge‐two IWP in K_2_Mg_2_O_3_, the charge‐four IWP is also localized at the center of 3D BZ and compensated by the surrounded WNWs (see **Figure** **4**a). Moreover, the charge‐four IWP can not exhibit any quadruple‐helicoid surface arc state, apart from a quadratic Dirac cone at the point $\bar{\Gamma }$ and near the frequency 11.188 THz. More importantly, the four‐fold fan‐shape surface state appears clearly in the square regime *l*_3_ of the isofrequency surface contours (see Figure 4f). As discussed above, this fan‐shape surface state is also robust, because it is generated both by charge‐four IWP and the surrounded WNWs, and associated with the three‐fold rotation symmetry $\left\{C_{31}^{-}|000\right\}$ in the *k*_*z*_‐direction. As expected, the number of fan leaves in them is equal to Chern number (i.e., +4) of charge‐four IWP, verifying further the conclusion that the multi‐fold fan‐shape surface state is a new kind of surface state and can be used to identify the Chern number of IWPs.

Furthermore, the surface states at the frequency 11.189 THz, away from the band crossing point, are also provided in Figure 4g,h. Although these surface states are contributed partially by trivial bulk states, the basic characters of four‐fold fan‐shape surface state displays well. Besides, the surface states in the (100) plane are provided in Figure S2 (Supporting Information). One may find that the four‐fold fan‐shape geometric configuration in them appear distinctly in that surface plane. These two additional evidences support further the robustness of the four‐fold fan‐shape surface state.

## Conclusion

In summary, by using symmetry analysis and first‐principles calculations, we uncovered a new kind of Weyl phonons, that is, IWPs in phononic crystals, which are circumvented from the original Nielsen‐Ninomiya no‐go theorem. IWPs can be classified into three categories, including charge‐one, charge‐two and charge‐four IWPs, and their projections in surfaces BZ are not connected by any surface arc states. However, the Berry curvature field of those IWPs are absorbed by the surrounded WNWs, a new class of surface states, that is, the multi‐fold fan‐shape surface states, are exhibited in surfaces BZ to form the compensation effect between the IWPs and the surrounded WNWs, which establish the fundamental properties of topological Weyl complex composed of IWPs and WNWs. Importantly, the number of fan leaves in them is tightly associated with the Chern number of IWPs, providing an effective way to identify the IWPs and their Chern numbers in bosonic systems. To confirm further the existence of this new class of surface state, we studied further the phononic structures of two real material samples, that is, K_2_Mg_2_O_3_ in SG No. 96 and Nb_3_Al_2_N in SG No. 213. Note that these new findings can be expanded to the isolated WPs existing in electronic, photonic and acoustic systems, and the related multi‐fold fan‐shape surface state can be observed experimentally in topological materials hosting the IWPs with large charges. Moreover, after carefully checking the symmetries in all SGs 230, we find that charge‐three isolated Weyl phonons can not exist in real phononic crystals. Nevertheless, charge‐three isolated WPs can exist in fermion systems, especially in the high‐symmetry lines Γ‐A in SGs 168‐173 and 177‐182.

Additional notes: like other kinds of phononic surface states, the fan‐shape phononic surface states can also be applied to realize phonon‐based transport behaviors such as quantized circular photogalvanic effect, phonon‐based diode devices and low‐energy‐dissipation phonon‐based transport devices and others.^[^ ^53^, ^54^ ^]^ Nevertheless, in comparison with surface arc state and quadruple‐helix surface state, the fan‐shape phononic surface state may exhibit weaker transport signatures due to their divergence behavior or burying in trivial bulk states. However, if we obtain high‐quality material samples or design ideal acoustic crystals,^[^ ^55^ ^]^ we believe that this new kind of topological surface states may be observed easily in experiments. Additionally, to confirm our findings, IWPs and the related fan‐shape surface state in other SGs listed in Table 1 are also provided in Supporting Information, Section C– G.

## Experimental Section

### k · *p* Model Hamiltonian

Consider a two‐band model of IWPs with C=±2 or ±4 at the point Γ in SGs 96 and 213. Note that the point Γ in these two SGs possesses the T symmetry. Thus, the two‐band *k* · *p* Hamiltonian of IWPs could be derived as described below

(1) $$H_{kp}=g_{x}\left(k\right)\sigma_{x}+g_{y}\left(k\right)\sigma_{y}+g_{z}\left(k\right)\sigma_{z},$$

where *k* = (*k*_*x*_, *k*_*y*_, *k*_*z*_), σ_*x*, *y*, *z*_ represents the three Pauli matrices, and *g*_*x*, *y*, *z*_(*k*) represents the complex functions versus *k*_*x*_, *k*_*y*_ and *k*_*z*_. In the momentum space, the T symmetry gives the following relation

(2) $$TH_{kp}\left(k\right)T^{-1}=H_{kp}\left(-k\right),$$

where *T* was a complex conjugate operator under 2D irreps, that is, Γ_5_ in SG 96 and Γ_3_ in SG 213. Under this operation, *g*_*x*_(*k*) and *g*_*z*_(*k*) were even functions, while *g*_*y*_(*k*) is an odd one. With these relations, the expanded *k* · *p* Hamiltonian versus *k* in low‐energy region can be written as

(3) $$\begin{array}{c} \begin{array}{cc} g_{x}\left(k\right) & =\sum_{i,j=x,y,z}a_{x}^{ij}k_{i}k_{j},\\ g_{y}\left(k\right) & =\sum_{i=x,y,z}a_{y1}^{i}k_{i}+\sum_{i,j,m=x,y,z}a_{y2}^{ijm}k_{i}k_{j}k_{m},\\ g_{z}\left(k\right) & =\sum_{i,j=x,y,z}a_{z}^{ij}k_{i}k_{j}. \end{array} \end{array}$$

Then, other required symmetry operations were further considered in SGs 96 and 213. Here, the representation matrixes for $\left\{C_{4z}^{+}|00\frac{3}{4}\right\}$ ($C_{4z}^{+}$: $xyz\mapsto \bar{y}xz$) and $\left\{C_{2x}|\frac{1}{2}\frac{1}{2}0\right\}$ at Γ in SG 96 can be described as $C_{4z}^{+}=-i\sigma_{y}$ and *C*_2*x*_ = −σ_*x*_. Under these two operations, the final *k* · *p*‐invariant Hamiltonian with C=±2 can be obtained as,

(4) $$H_{kp}=a_{x}^{xx}\left(k_{x}^{2}-k_{y}^{2}\right)\sigma_{x}+a_{y1}^{z}k_{z}\sigma_{y},$$

where $a_{x}^{xx}$ and $a_{y}^{z}$ are constant coefficients. According to the above two‐band *k* · *p* Hamiltonian, we conclude that the dispersions display a *k*‐type feature along the (001) direction, while a *k* ^2^‐type feature along other directions in phononic bands. Note that this *k* · *p* model is also applicable in SG 92.

The, the representation matrixes for $\left\{C_{31}^{-}|000\right\}$ ($C_{31}^{-}$:*xyz*↦*yzx*), $\left\{C_{2x}|\frac{1}{2}0\frac{1}{2}\right\}$, $\left\{C_{2z}|\frac{1}{2}0\frac{1}{2}\right\}$ and $\left\{C_{2a}|\frac{3}{4}\frac{1}{4}\frac{1}{4}\right\}$ (*C*_2*a*_:$xyz\mapsto yx\bar{z}$) at Γ in SG 213 can be described as

(5) $$\begin{array}{c} \begin{array}{cc} C_{31}^{-} & =\left[\begin{array}{cc} -\frac{1}{2} & \frac{\sqrt{3}}{2}\\ -\frac{\sqrt{3}}{2} & -\frac{1}{2} \end{array}\right],\\ C_{2x} & =E,C_{2z}=C_{2x},C_{2a}=\sigma_{x}. \end{array} \end{array}$$

Under the above four operations, the final *k* · *p*‐invariant Hamiltonian with C=±4 can be obtained as,

(6) $$\begin{array}{c} \begin{array}{c} H_{kp}=a_{x}^{xx}\left(k_{x}^{2}-k_{y}^{2}\right)\sigma_{x}+a_{y2}^{xyz}k_{x}k_{y}k_{z}\sigma_{y}\\ +\frac{1}{\sqrt{3}}a_{x}^{xx}\left(k_{x}^{2}+k_{y}^{2}-2k_{z}^{2}\right)\sigma_{z}, \end{array} \end{array}$$

where $a_{x}^{xx}$ and $a_{y2}^{xyz}$ were constant coefficients. According to the above Hamiltonian, it was concluded that the dispersions display a *k* ^3^‐type feature along the [111] direction, while a *k* ^2^‐type feature along other directions in phononic bands. Note that the above *k* · *p* model Hamiltonian was also applicable in SGs 198 and 212.

### First‐Principles Calculations

The phononic dispersions of realistic materials were calculated by the density functional theory using the Vienna ab initio Simulation Package (VASP).^[^ ^56^, ^57^, ^58^ ^]^ The phononic Hamiltonian based on tight‐binding (TB) model, the open‐source software Wanniertools code^[^ ^59^ ^]^ and the surface Green's functions^[^ ^60^ ^]^ were used to study the phononic states and their surface states. Wilson loop method^[^ ^61^ ^]^ was used to calculate the Chern numbers of IWPs, and the program *ir*2*tb* on the phononic Hamiltonian of TB model^[^ ^62^ ^]^ was adopted to calculate the irreps of phononic bands.

## Conflict of Interest

The authors declare no conflict of interest.

## Supporting information

Supporting Information

## Data Availability

The data that support the findings of this study are available on request from the corresponding author. The data are not publicly available due to privacy or ethical restrictions.
