# Supplementary material for: Multi‐Fold Fan‐Shape Surface State Induced by an Isolated Weyl Phonon Beyond No‐Go Theorem
Source: Adv Sci (Weinh). 2023 Apr 23;10(18):2207508. doi: 10.1002/advs.202207508 (PMC10288247; doi:10.1002/advs.202207508)
Supplement: Supplementary file 1 — Supporting Information [file ADVS-10-2207508-s001.pdf]

Supplemental Information for "Multi-fold fan-shape surface state induced by an isolated Weyl phonon beyond no-go theorem"

Hua-Hua Fu,[1,2,*] Qing-Bo Liu,[1,2] Zhe-Qi Wang,[1,2] and Xiang-Feng Yang[1,2]

[1] *School of Physics and Wuhan National High Magnetic Field Center, Huazhong University of Science and Technology, Wuhan 430074, People's Republic of China.*

[2] *Institute for Quantum Science and Engineering, Huazhong University of Science and Technology, Wuhan, Hubei 430074, China.*

[*] hhfu@hust.edu.cn

.

In this file of Supplemental Information file, the fan-shape surface states in the (100) plane of two real material samples $K_2Mg_2O_3$ and Nb_3Al_2N in Sections A and B, provide other 45 thermodynamically stable materials possessing IWPs in SGs 92, 96, 198, 212, and 213, which are listed in Table-I in the main context. Among them, IWPs in SGs 92 and 96 with the charge of ± 2 will be presented in Section C and D, IWPs in SGs 198, 212 and 213 with the charge of ± 4 will be presented in Section E, F and G, respectively.

CONTENTS

A. The fan-shape surface states in the (100) plane of $K_2Mg_2O_3$ 3

B. The fan-shape surface states in the (100) plane of Nb_3Al_2N 3

C. IWPs with the charge of ± 2 in SG 92 and the related realistic materials 4

D. IWPs with the charges of ± 2 in SG 96 and the related realistic materials 13

E. IWPs with the charges of ± 4 in SG 198 and the related realistic materials 20

F. IWPs with the charges of ± 4 in SG 212 and the related realistic materials 37

G. IWPs with the charge of ± 4 in SG 213 and the related realistic materials 41

References 54

A. THE FAN-SHAPE SURFACE STATES IN THE (100) PLANE OF $K_2MG_2O_3$

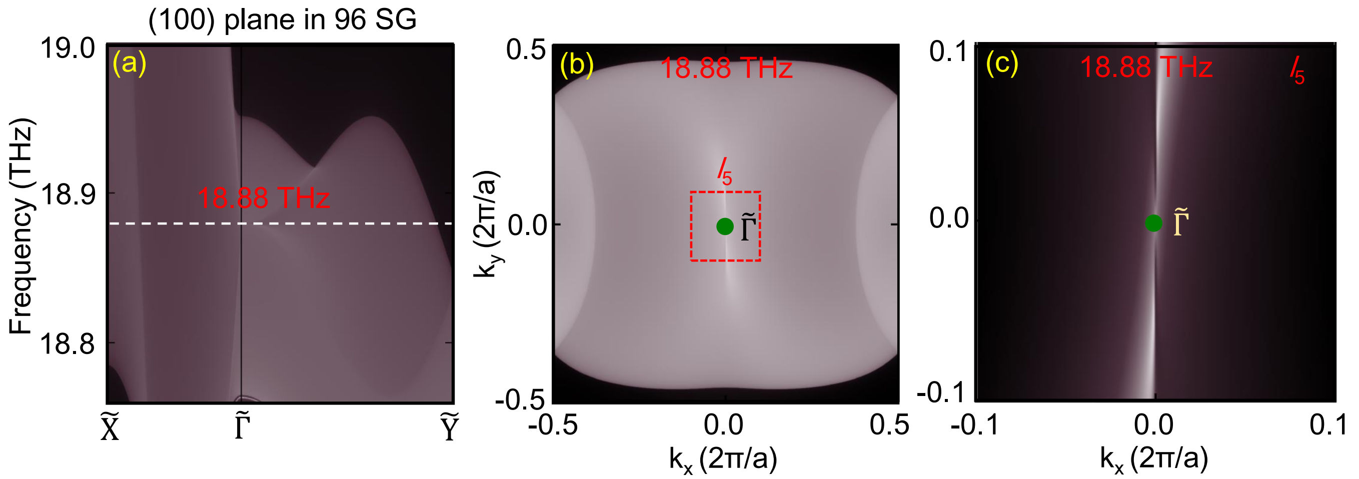

FIG. S1. The fan-shape surface states in the (100) plane of $K_2Mg_2O_3$. (a) The surface states in the (100) plane. (b) and (c) The isofrequency surface contours at the frequency 18.88 THz.

B. THE FAN-SHAPE SURFACE STATES IN THE (100) PLANE OF NB_3AL_2N

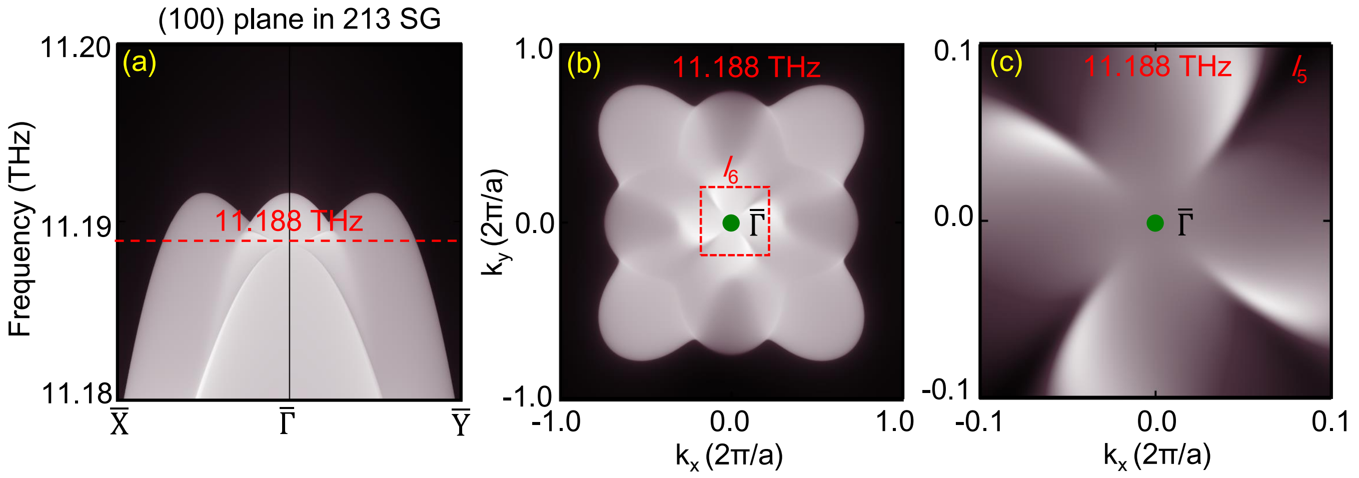

FIG. S2. The fan-shape surface states in the (100) plane of Nb_3Al_2N. (a) The surface states in the (100) plane. (b) and (c) The isofrequency surface contours at the frequency 11.188 THz.

C. IWPS WITH THE CHARGE OF ± 2 IN SG 92 AND THE RELATED REALISTIC MATERIALS

In this section, we present 8 realistic materials candidates in SG No. 92 in Table I in the main context, to illustrate the existence of the IWPs with the charge of ± 2 in realistic materials. Here, we investigate the all thermodynamically stable materials including CdP_2, GeO_2, SiO_2, ZnP_2, $BaPt_2S_3$, $LiAlO_2$, Pt_3I_8 and $Rb_2Be_2O_3$ in SG 92 in Materials Project (MP) [1]. Firstly, we draw their primitive unit cells and the first BZs to show their crystal structures. Then, we calculate their phonon dispersions in the first BZ to show the existence of IWPs. The calculated methods are adopted as the same as those described in the main text. The crystallographics data of CdP_2, GeO_2, SiO_2, ZnP_2, $BaPt_2S_3$, $LiAlO_2$, Pt_3I_8 and $Rb_2Be_2O_3$ are adopted from Ref. [1] and the primitive cells are shown in Fig. S3(a), S4(a), S5(a), S6(a), S7(a), S8(a), S9(a) and S10(a), and the corresponding BZs are shown in Fig. S3(b), S4(b), S5(b), S6(b), S7(b), S8(b), S9(b) and S10(b). To confirm the above results from the symmetry analysis, the phononic dispersions and the some twofold bands (red area) at the point Γ of CdP_2, GeO_2, SiO_2, ZnP_2, $BaPt_2S_3$, $LiAlO_2$, Pt_3I_8 and $Rb_2Be_2O_3$ from *ab initio* calculations [2] are illustrated in Fig. S3(c), S4(c), S5(c), S6(c), S7(c), S8(c), S9(c) and S10(c).

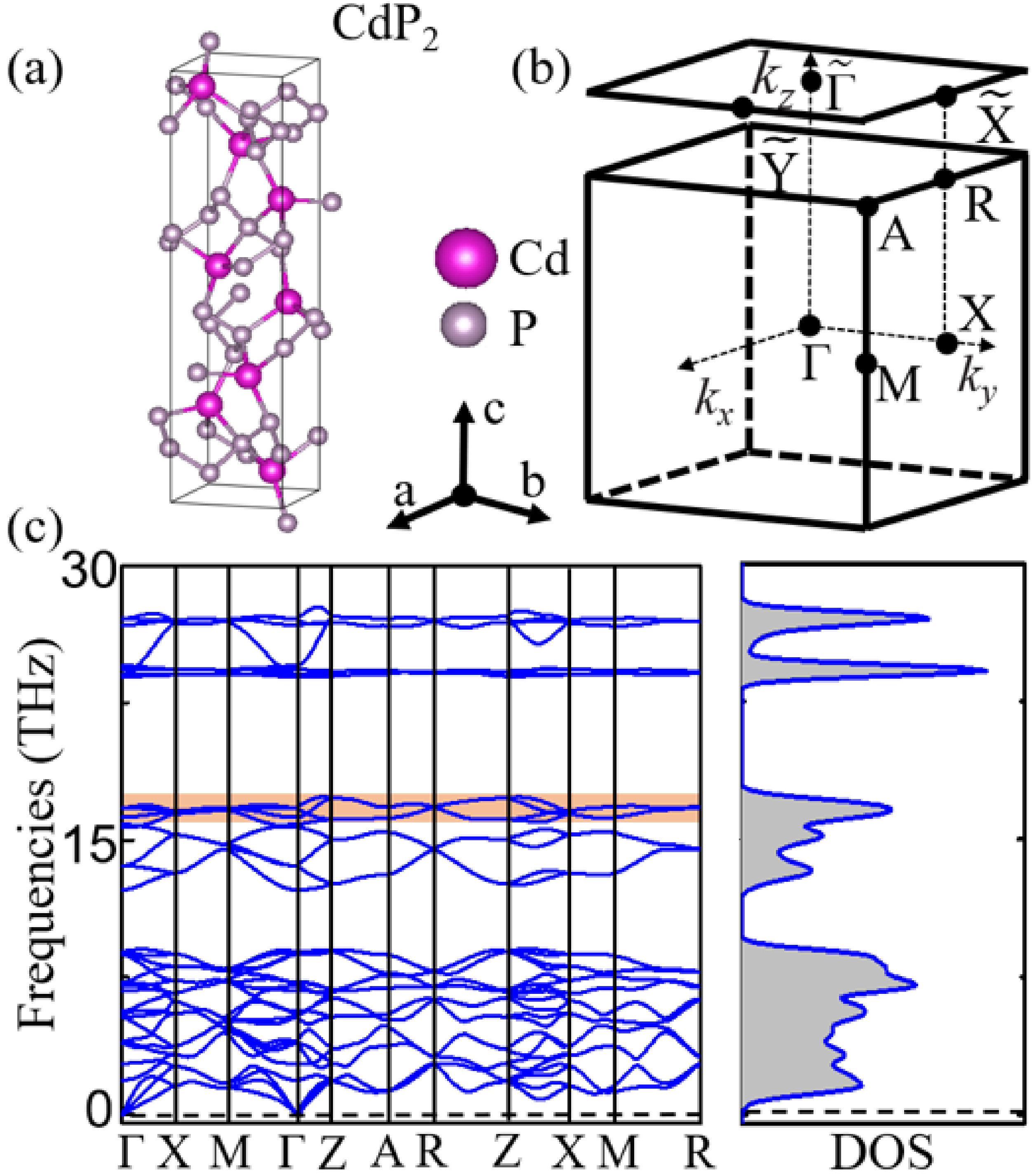

FIG. S3. A realistic candidate CdP_2 in SG 92. (a) A unit cell contains 8 Cd and 16 P atoms. (b) The first BZ of CdP_2. (c) The phononic dispersions along the high-symmetry paths and the phononic density of states (DOSs) of CdP_2. It is clearly seen that the a twofold IWP is localized at the high-symmetry point Γ (a red box), indicating the existence of IWP in this material.

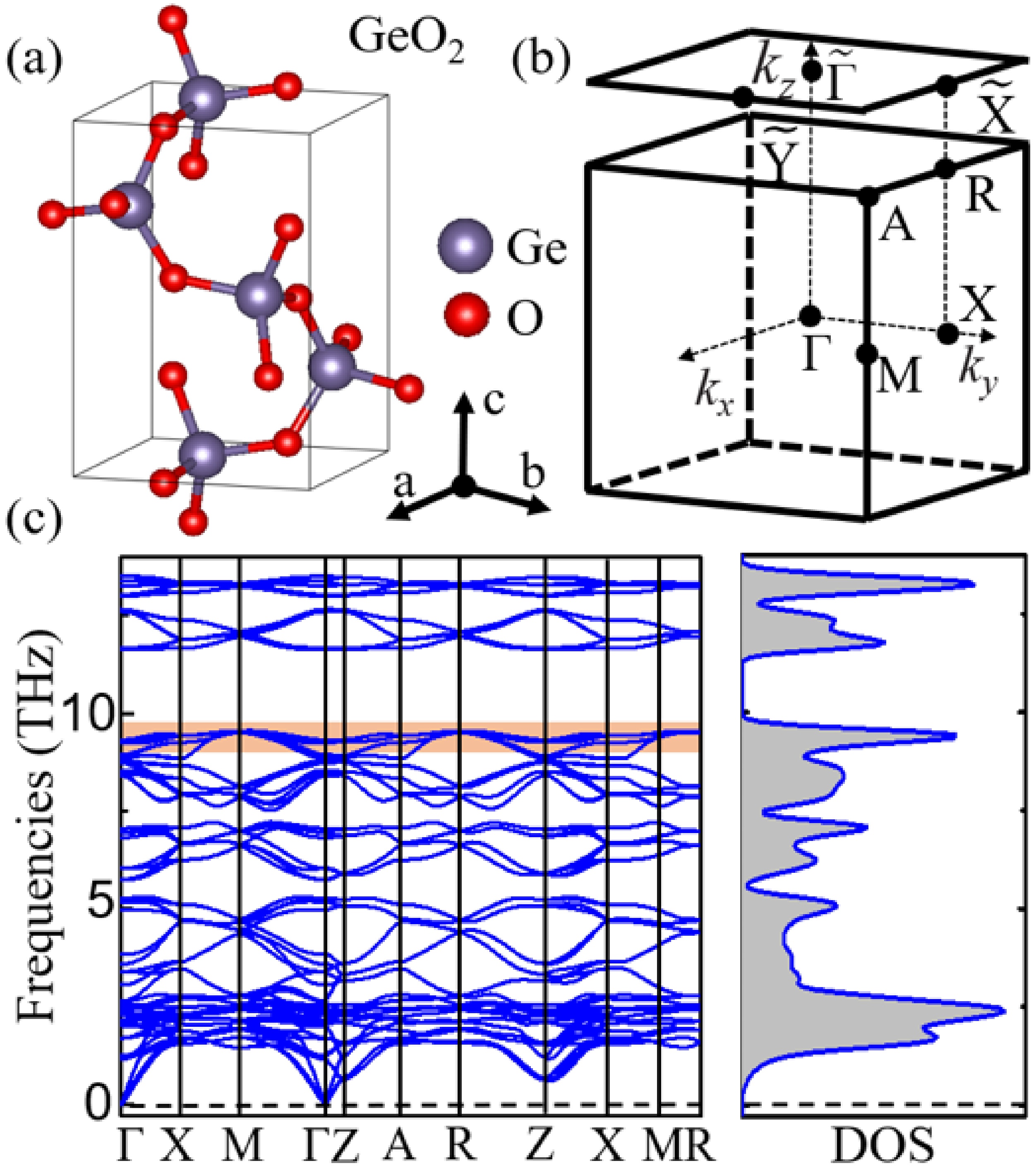

FIG. S4. A realistic candidate GeO_2 in SG 92. (a) A unit cell contains 8 Ge and 16 O atoms. (b) The first BZ of GeO_2. (c) The phononic dispersions along the high-symmetry paths and phononic density of states (DOSs) of GeO_2. It is clearly seen that the a twofold IWP is localized at the high-symmetry point Γ (a red box), indicating the existence of IWP in this material.

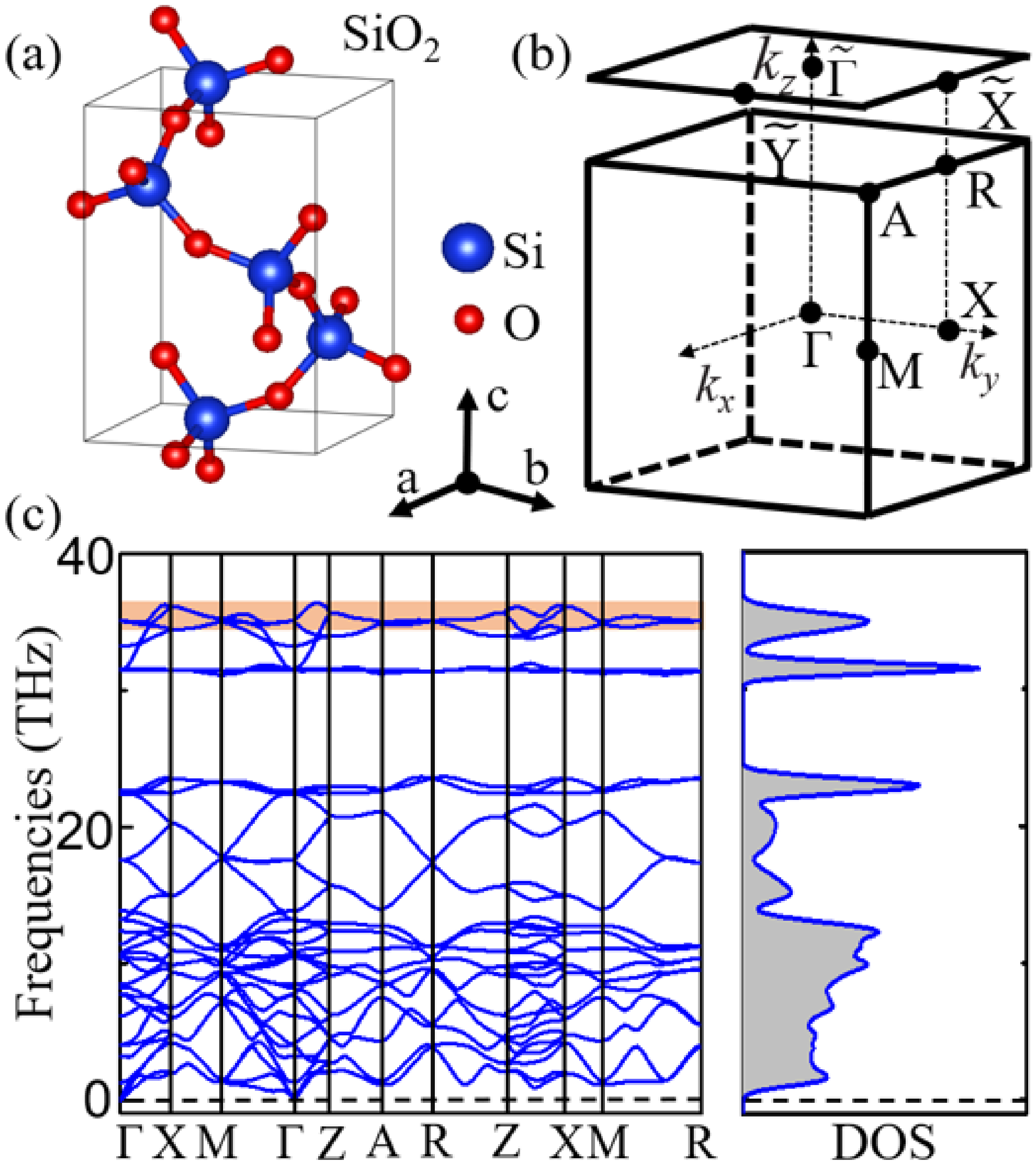

FIG. S5. A realistic candidate SiO_2 in SG 92. (a) A unit cell contains 4 Si and 8 O atoms. (b) The first BZ of SiO_2. (c) The phonon dispersions along high-symmetry paths and phononic density of states (DOSs) of SiO_2. It is clearly seen that the a twofold IWP is localized at the high-symmetry point Γ (a red box), indicating the existence of IWP in this material.

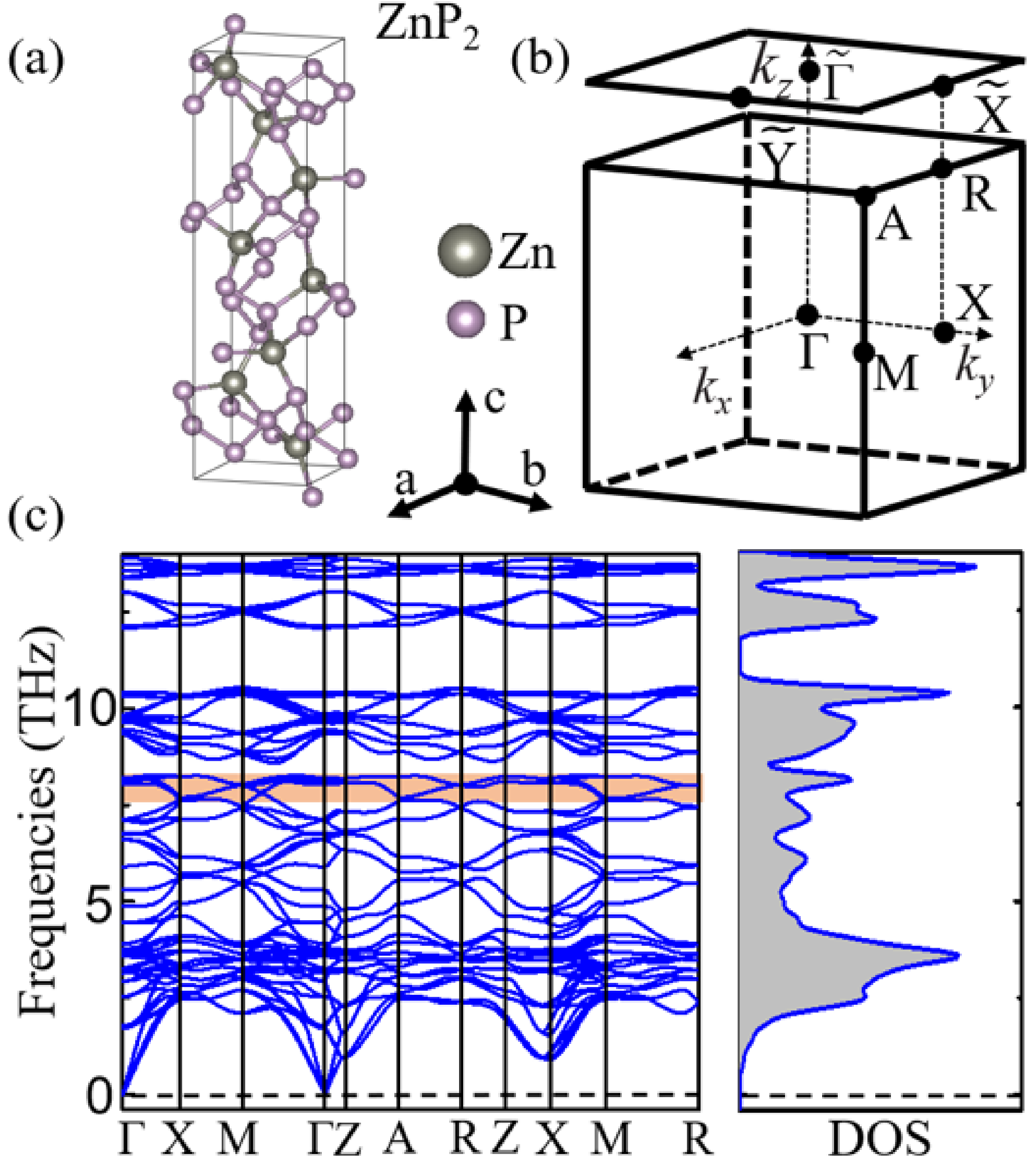

FIG. S6. A realistic candidate ZnP_2 in SG 92. (a) A unit cell contains 8 Zn and 16 P atoms. (b) The first BZ of ZnP_2. (c) The phononic dispersions along the high-symmetry directions and phononic density of states (DOSs) of ZnP_2. It is clearly seen that the a twofold IWP is localized at the high-symmetry point Γ (a red box), indicating the existence of IWP in this material.

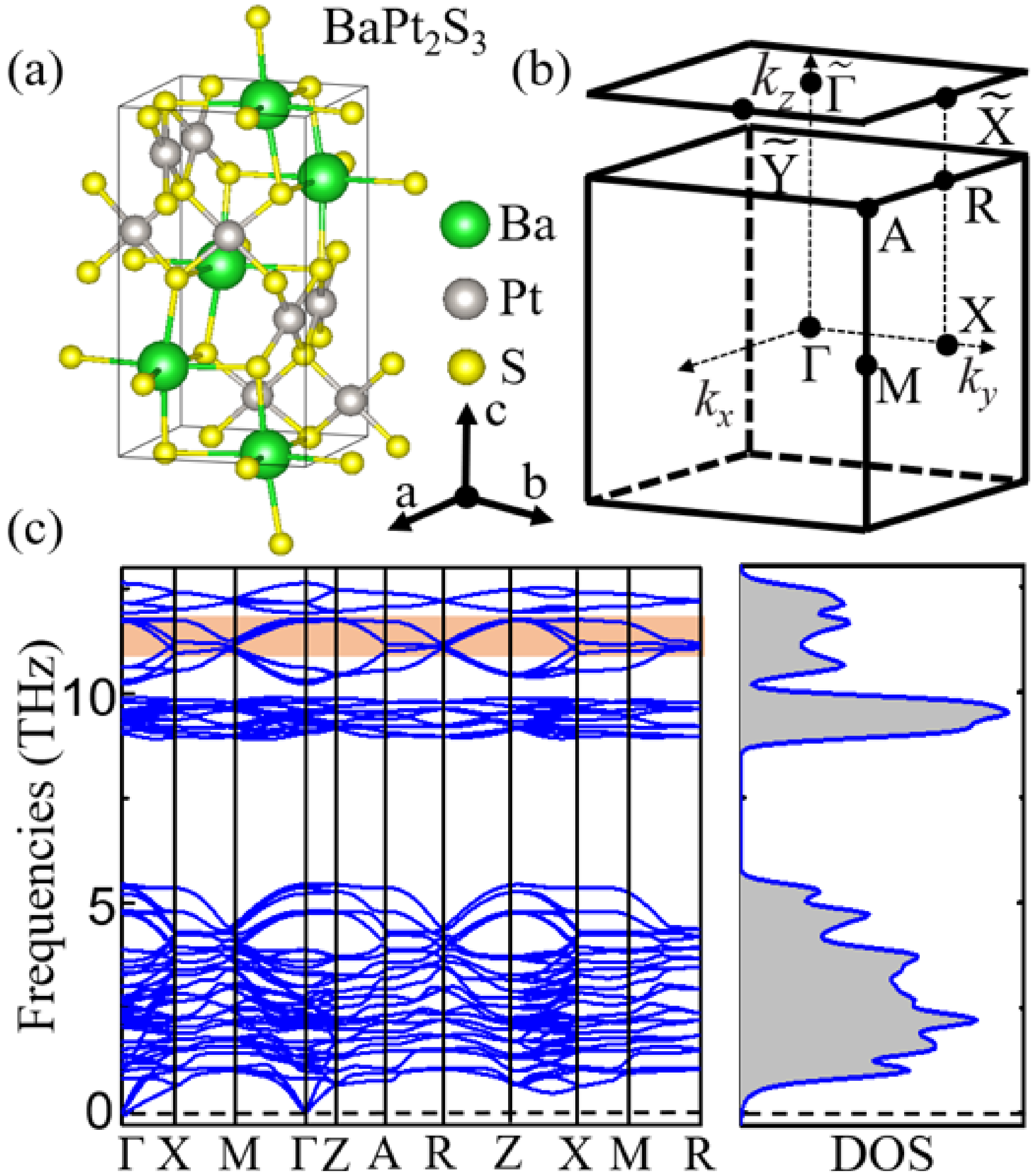

FIG. S7. A realistic candidate $BaPt_2S_3$ in SG 92. (a) A unit cell contains 4 Ba, 8 Pt and 12 P atoms. (b) The first BZ of $BaPt_2S_3$. (c) The phononic dispersions along high-symmetry directions and phononic density of states (DOSs) of $BaPt_2S_3$. It is clearly seen that the a twofold IWP is localized at the high-symmetry point Γ (a red box), indicating the existence of IWPs in this material.

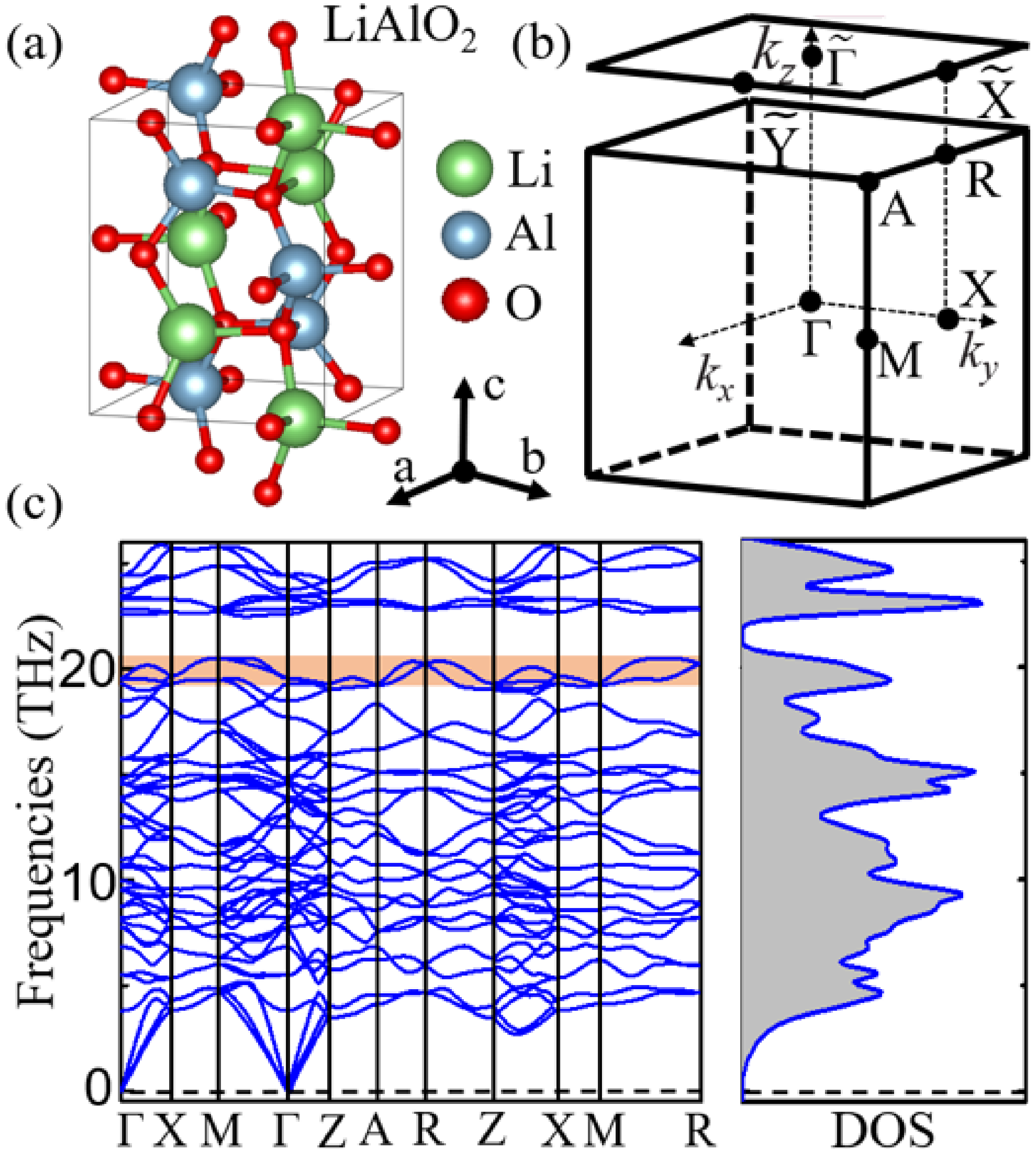

FIG. S8. A realistic candidate $LiAlO_2$ in SG 92. (a) A unit cell contains 4 Li, 4 Al and 8 O atoms. (b) The first BZ of $LiAlO_2$. (c) The phononic dispersions along the high-symmetry directions and the phononic density of states (DOSs) of $LiAlO_2$. It is clearly seen that the a twofold IWP is localized at the high-symmetry point Γ (a red box), indicating the existence of IWP in this material.

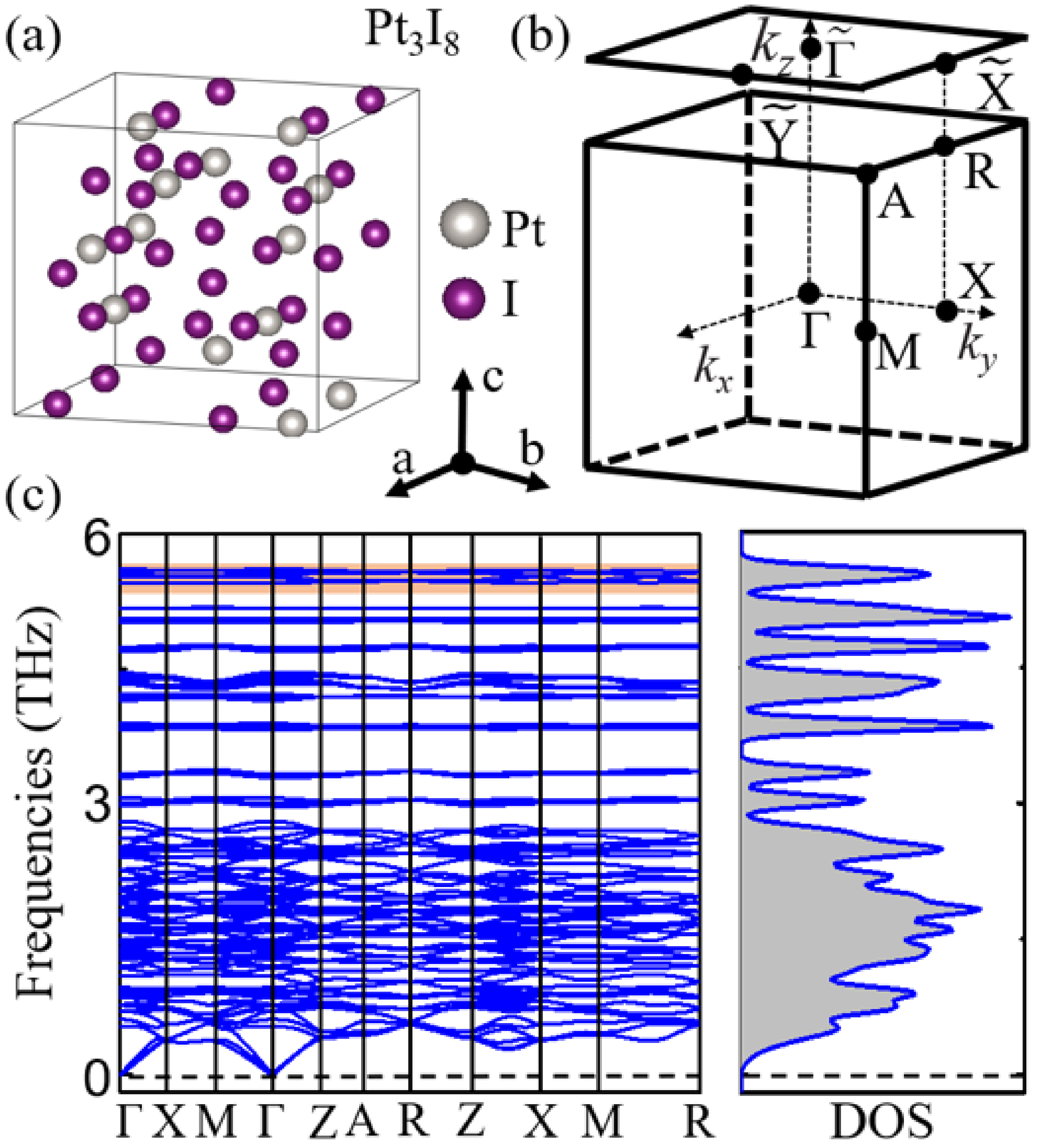

FIG. S9. A realistic candidate Pt_3I_8 in SG 92. (a) A unit cell contains 12 Pt and 32 I atoms. (b) The first BZ of Pt_3I_8. (c) The phononic dispersions along the high-symmetry directions and phononic density of states (DOSs) of Pt_3I_8. It is clearly seen that the a twofold IWP is localized at the high-symmetry point Γ (a red box), indicating the existence of IWP in this material.

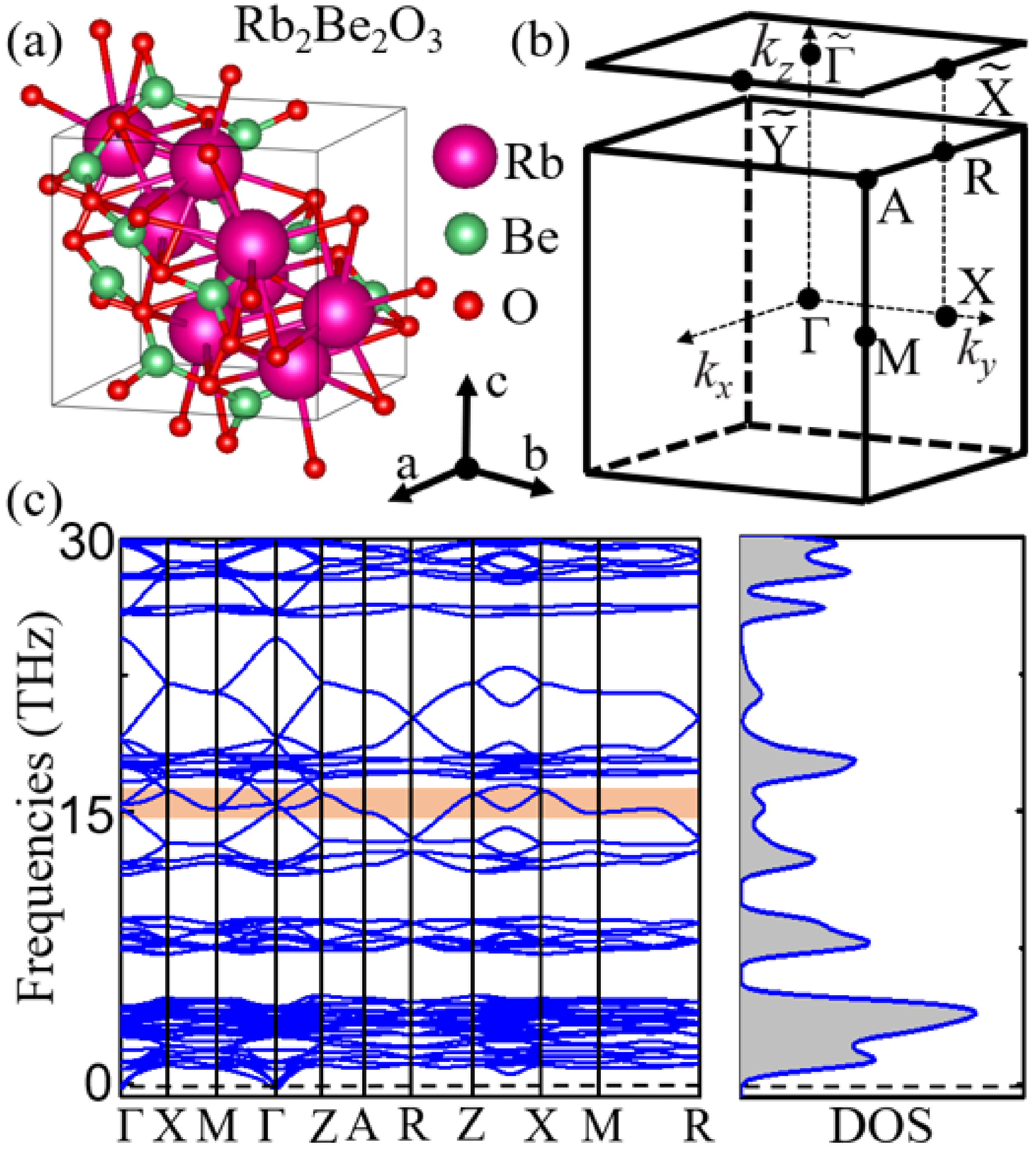

FIG. S10. A realistic candidate $Rb_2Be_2O_3$ in SG 92. (a) A unit cell contains 8 Rb, 8 Be and 12 O atoms. (b) The first BZ of $Rb_2Be_2O_3$. (c) The phononic dispersions along the high-symmetry directions and phononic density of states (DOSs) of $Rb_2Be_2O_3$. It is clearly seen that the a twofold IWP is localized at the high-symmetry point Γ (a red box), indicating the existence of IWP in this material.

D. IWPS WITH THE CHARGES OF ±2 IN SG 96 AND THE RELATED REALISTIC MATERIALS

In this section, we present another six realistic materials candidates in SG No. 96 in Table I in the main context, to illustrate the existence of the IWPs with the charges of ±2 in realistic materials. Here, we investigate the all thermodynamically stable materials including CdP_2, SiO_2, ZnP_2, Ag_2HgO_2, $MgAs_4$ and $Na_2Zn_2O_3$ in SG 96 in Materials Project (MP) [1]. And we first draw their primitive unit cells and the first BZs to show the crystal structures. Then, we calculate their phononic dispersions in the first BZ to describe the existence of IWPs. The calculation methods are the same as those described in the main text. The crystallographics data of CdP_2, SiO_2, ZnP_2, Ag_2HgO_2, $MgAs_4$ and $Na_2Zn_2O_3$ are adopted from Ref. [1] and the primitive cells are shown in Fig. S11(a), S12(a), S13(a), S14(a), S15(a) and S16(a), and the corresponding BZs are shown in Fig. S11(b), S12(b), S13(b), S14(b), S15(b) and S16(b). To confirm the above results from the symmetry analysis, the phononic dispersions and the twofold bands (red area) at the point Γ of CdP_2, SiO_2, ZnP_2, Ag_2HgO_2, $MgAs_4$, $Na_2Zn_2O_3$ from *ab initio* calculations [2] are illustrated in Fig. S11(c), S12(c), S13(c), S14(c), S15(c) and S16(c).

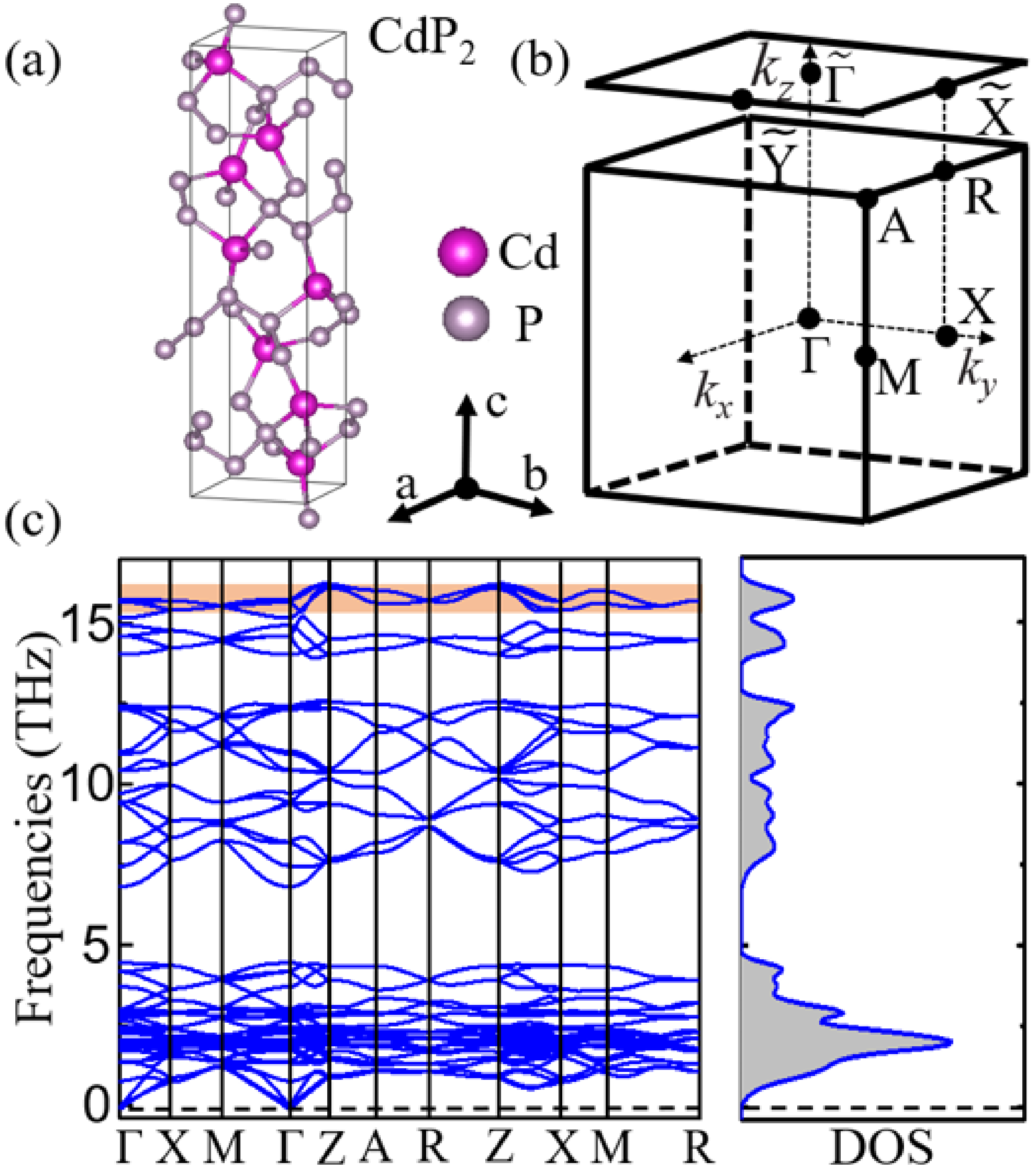

FIG. S11. A realistic candidate CdP_2 in SG 96. (a) A unit cell contains 8 Cd and 16 P atoms. (b) The first BZ of CdP_2. (c) The phononic dispersions along high-symmetry directions and phononic density of states (DOSs) of CdP_2. It is clearly seen that the a twofold IWP is localized at the high-symmetry point Γ (a red box), indicating the existence of IWP in this material.

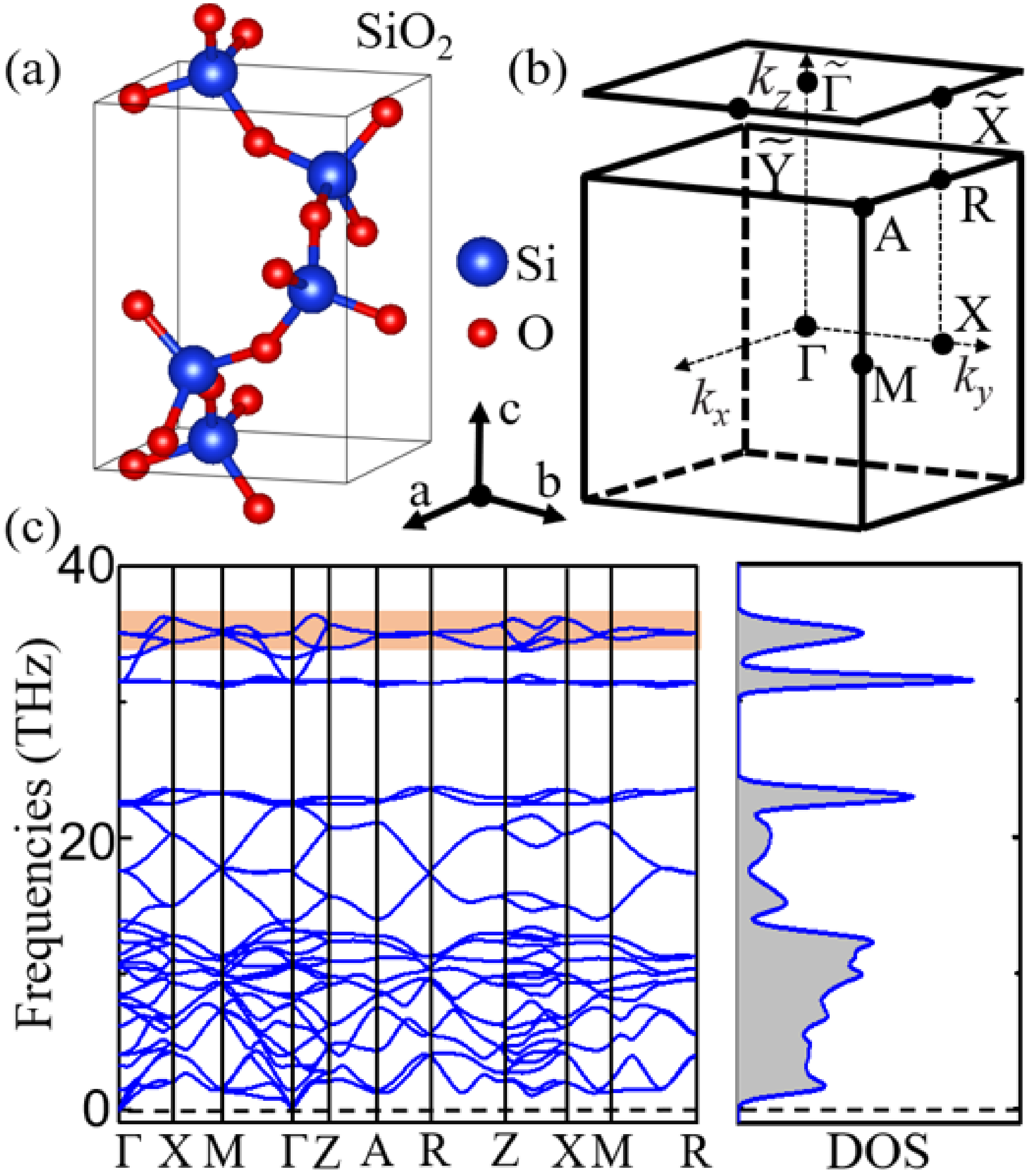

FIG. S12. A realistic candidate SiO_2 in SG 96. (a) A unit cell contains 4 Si and 8 O atoms. (b) The first BZ of SiO_2. (c) The phonon dispersions along high-symmetry directions and the phononic density of states (DOSs) of SiO_2. It is clearly seen that the a twofold IWP is localized at the high-symmetry point Γ (a red box), indicating the existence of IWP in this material.

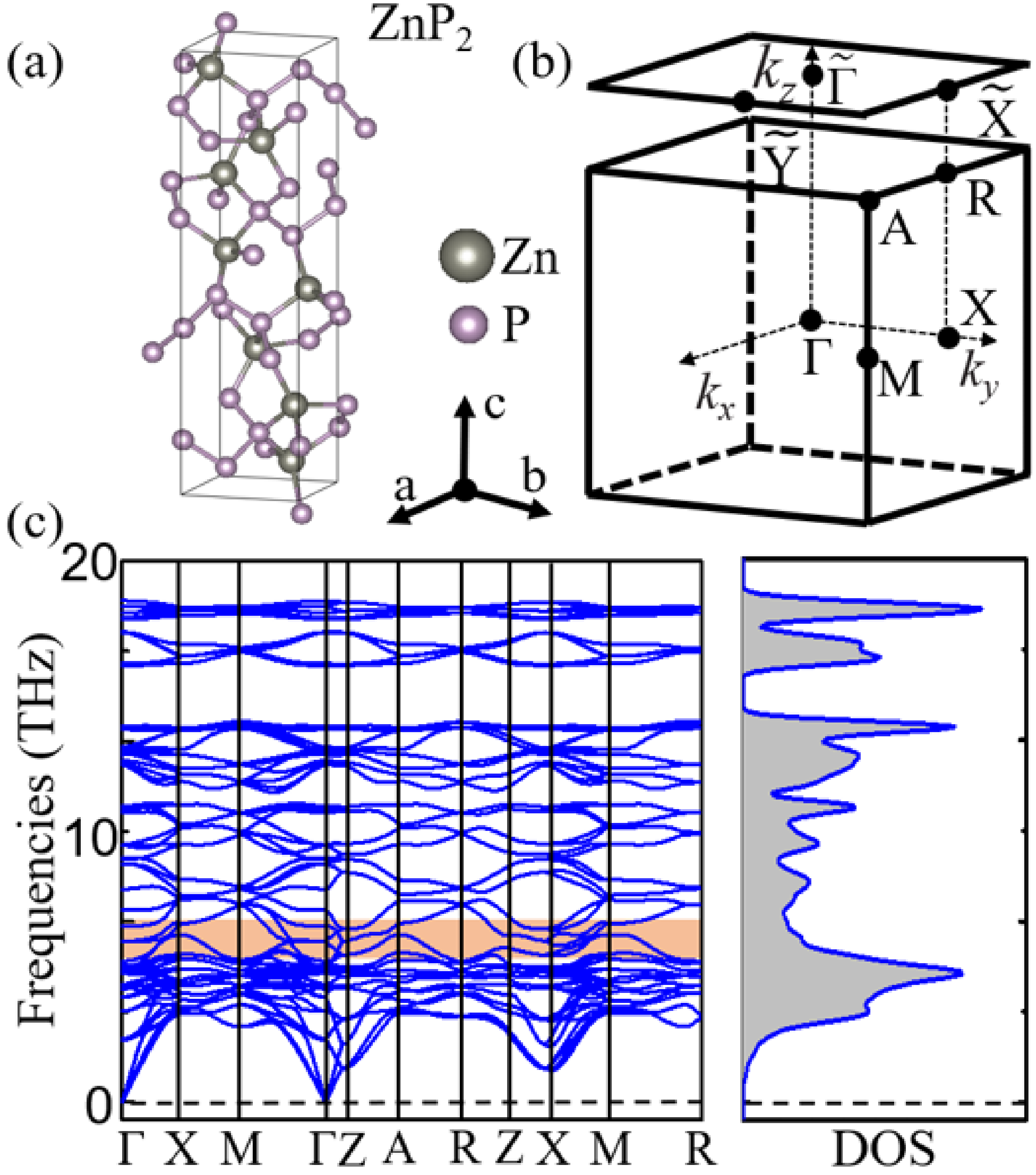

FIG. S13. A realistic candidate ZnP_2 in SG 96. (a) A unit cell contains 8 Zn and 16 O atoms. (b) The first BZ of ZnP_2. (c) The phononic dispersions along high-symmetry directions and the phononic density of states (DOSs) of ZnP_2. It is clearly seen that the a twofold IWP is localized at the high-symmetry point Γ (a red box), indicating the existence of IWP in this material.

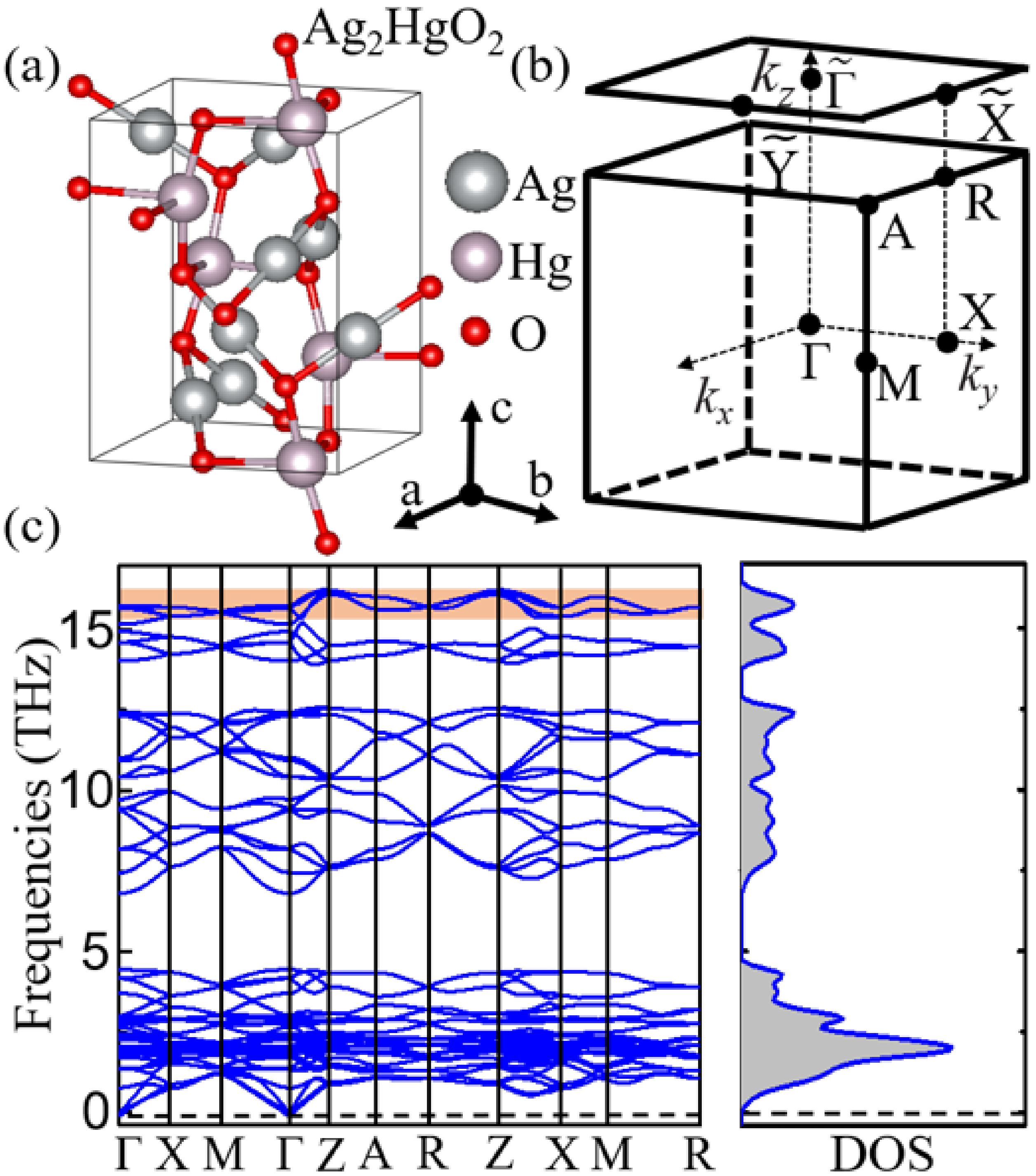

FIG. S14. A realistic candidate Ag_2HgO_2 in SG 96. (a) A unit cell contains 8 Ag, 4 Hg and 8 O atoms. (b) The first BZ of Ag_2HgO_2. (c) The phonon dispersions along the high-symmetry directions and the phononic density of states (DOSs) of Ag_2HgO_2. It is clearly seen that the a twofold IWP is localized at the high-symmetry points Γ (a red box), indicating the existence of IWP in this material.

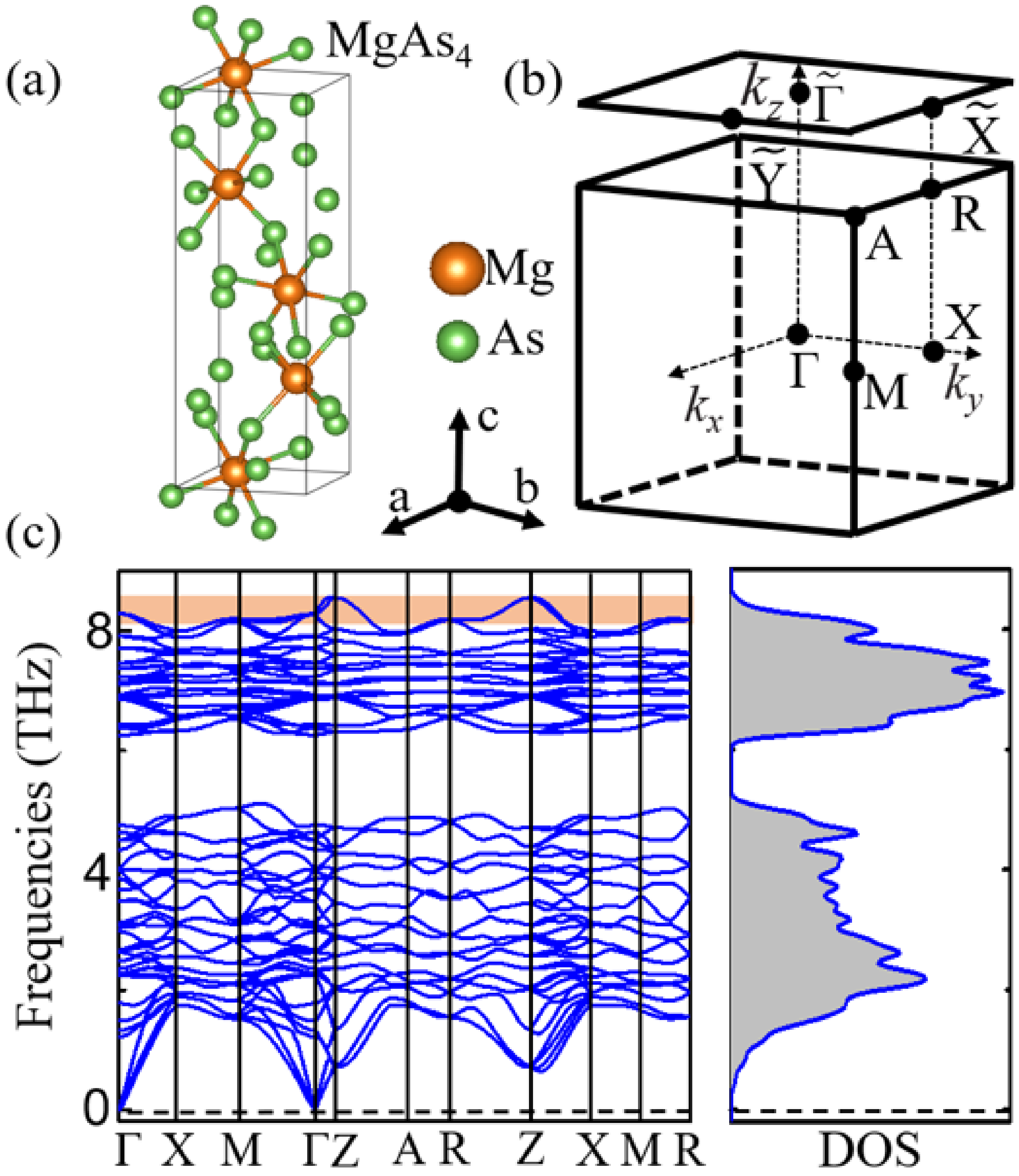

FIG. S15. A realistic candidate $MgAs_4$ in SG 96. (a) A unit cell contains 4 Mg and 16 As atoms. (b) The first BZ of $MgAs_4$. (c) The phononic dispersions along the high-symmetry directions and phononic density of states (DOSs) of $MgAs_4$. It is clearly seen that the a twofold IWP is localized at the high-symmetry point Γ (a red box), indicating the existence of IWP in this material.

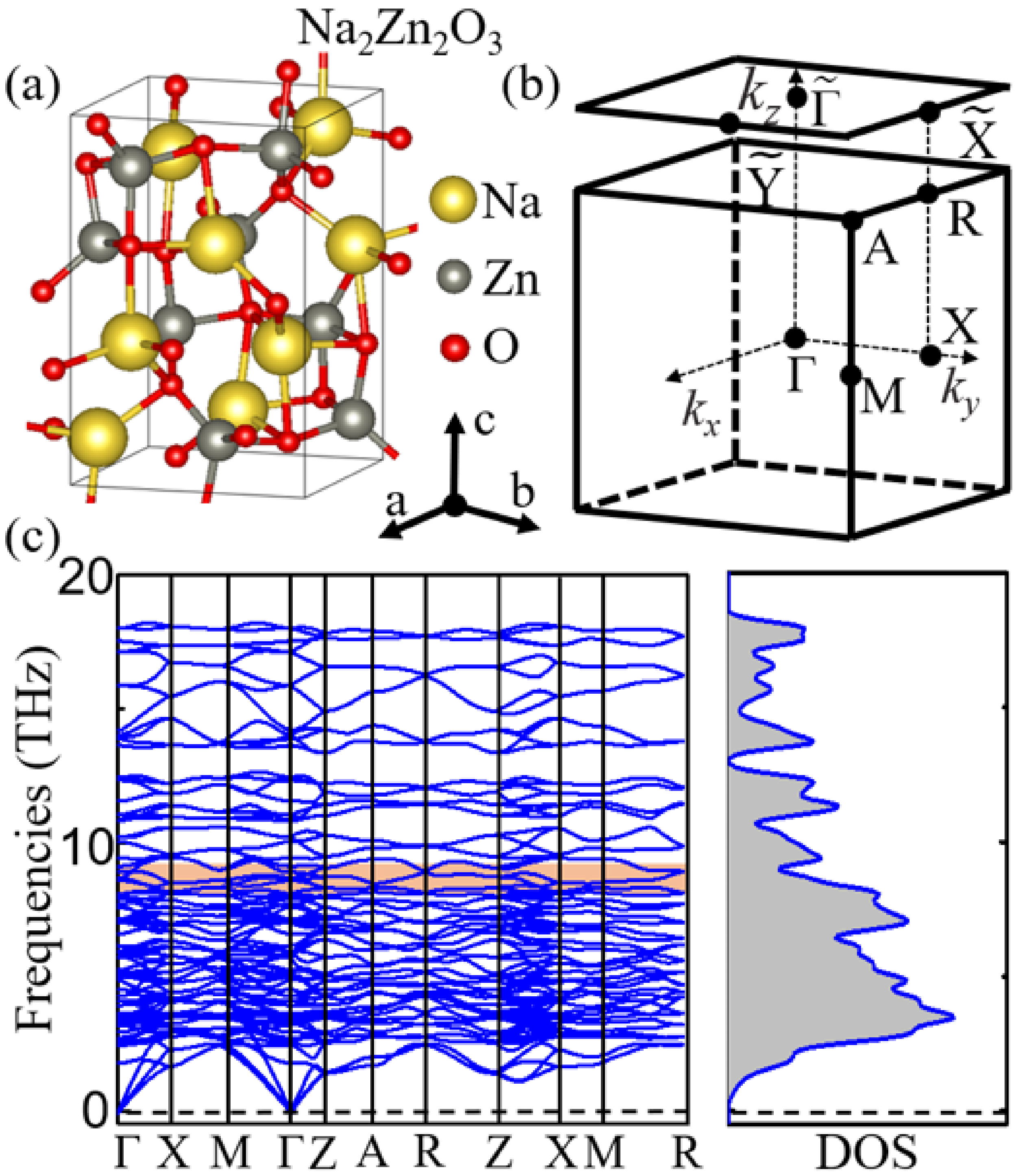

FIG. S16. A realistic candidate $Na_2Zn_2O_3$ in SG 96. (a) A unit cell contains 8 Na, 8 Zn and 12 As atoms. (b) The first BZ of $Na_2Zn_2O_3$. (c) The phononic dispersions along the high-symmetry directions and phononic density of states (DOSs) of $Na_2Zn_2O_3$. It is clearly seen that the a twofold IWP is localized at the high-symmetry point Γ (a red box), indicating the existence of IWP in this material.

E. IWPS WITH THE CHARGES OF ±4 IN SG 198 AND THE RELATED REALISTIC MATERIALS

Here, we present 16 realistic materials candidates in SG 198 in Table I in the main context, to illustrate the existence of the IWPs with the charge of ±4 in realistic materials. We investigate the all thermodynamically stable materials including $BaAsPt$, $BaPPt$, $BeAu$, $BiRhSe$, $BiTeIr$, $BiTePt$, $CoAsS$, $HfSO$, K_3SbO_3, $KMgBO_3$, $SbIrS$, $SiOs$, $SiRu$, $SrMgNiH_4$, $ZrSb$ and $ZrSeO$ in Materials Project (MP) [1]. Firstly, we draw their primitive unit cells and the first BZs to show the crystal structures. Then, we calculate their phononic dispersions in the first BZ to describe the existence of IWPs. The calculation methods are the same as those described in the main text. The crystallographics data of $BaAsPt$, $BaPPt$, $BeAu$, $BiRhSe$, $BiTeIr$, $BiTePt$, $CoAsS$, $HfSO$, K_3SbO_3, $KMgBO_3$, $SbIrS$, $SiOs$, $SiRu$, $SrMgNiH_4$, $ZrSb$ and $ZrSeO$ are adopted from Ref. [1] and the primitive cells are shown in Fig. S17(a), S18(a), S19(a), S20(a), S21(a), S22(a), S23(a), S24(a), S25(a), S26(a), S27(a), S28(a), S29(a), S30(a), S31(a) and S32(a), and the corresponding BZs are shown in Fig. S17(b), S18(b), S19(b), S20(b), S21(b), S22(b), S23(b), S24(b), S25(b), S26(b), S27(b), S28(b), S29(b), S30(b), S31(b) and S32(b). To confirm the above results from the symmetry analysis, the phononic dispersions and the some twofold bands (red area) at the point Γ of $BaAsPt$, $BaPPt$, $BeAu$, $BiRhSe$, $BiTeIr$, $BiTePt$, $CoAsS$, $HfSO$, K_3SbO_3, $KMgBO_3$, $SbIrS$, $SiOs$, $SiRu$, $SrMgNiH_4$, $ZrSb$ and $ZrSeO$ from the *ab initio* calculations [2] are illustrated in Fig. S17(c), S18(c), S19(c), S20(c), S21(c), S22(c), S23(c), S24(c), S25(c), S26(c), S27(c), S28(c), S29(c), S30(c), S31(c) and S32(c).

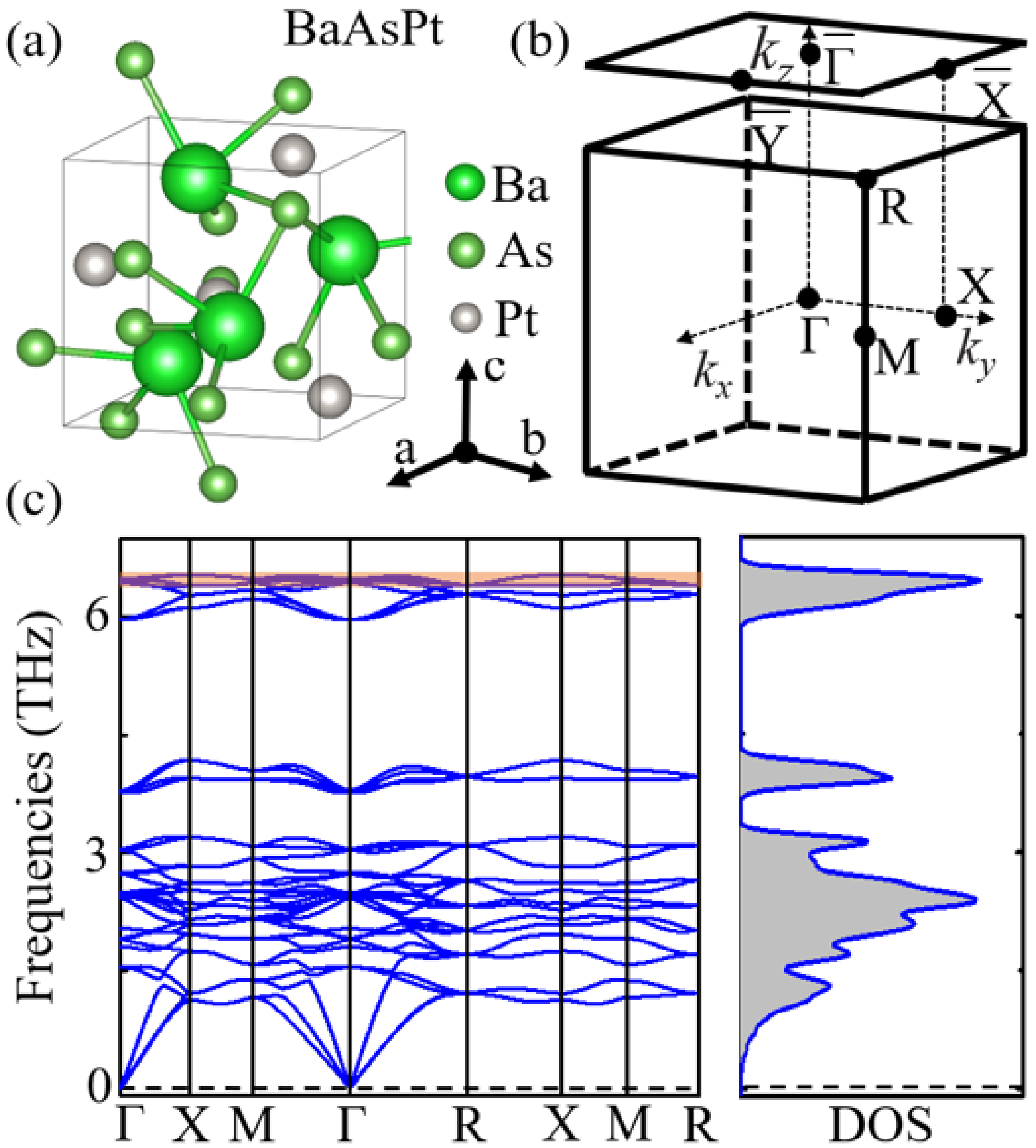

FIG. S17. A realistic candidate BaAsPt in SG 198. (a) A unit cell contains 4 Ba, 4 As and 4 Pt atoms. (b) The first BZ of BaAsPt. (c) The phononic dispersions along the high-symmetry directions and phononic density of states (DOSs) of BaAsPt. It is clearly seen that the a twofold IWP is localized at the high-symmetry point Γ (a red box), indicating the existence of IWP in this material.

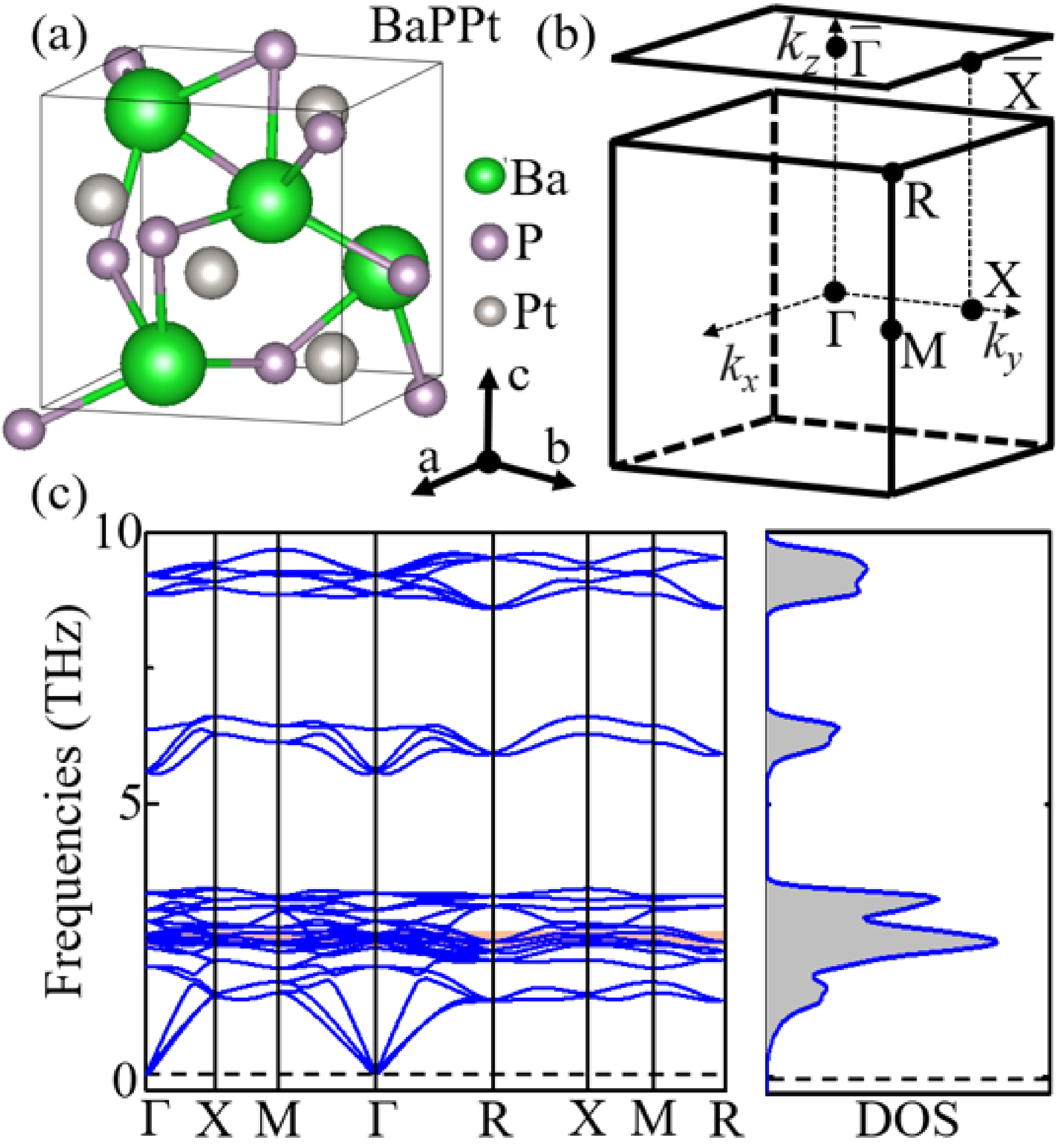

FIG. S18. A realistic candidate BaPPt in SG 198. (a) A unit cell contains 4 Ba, 4 P and 4 Pt atoms. (b) The first BZ of BaPPt. (c) The phononic dispersions along the high-symmetry directions and phononic density of states (DOSs) of BaPPt. It is clearly seen that the a twofold IWP is localized at the high-symmetry point Γ (a red box), indicating the existence of IWP in this material.

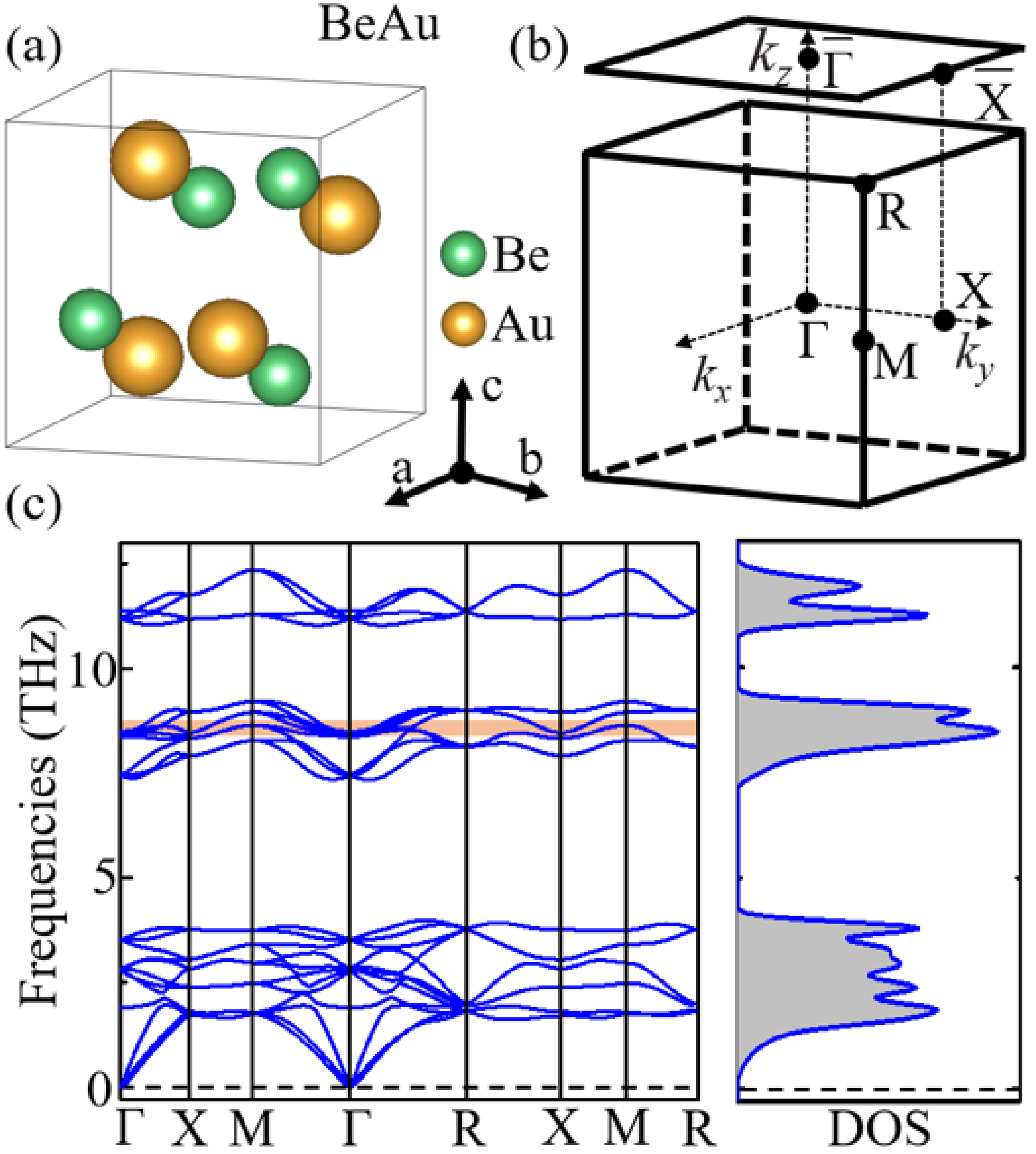

FIG. S19. A realistic candidate BeAu in SG 198. (a) A unit cell contains 4 Be and 4 Au atoms. (b) The first BZ of BeAu. (c) The phononic dispersions along the high-symmetry directions and the phononic density of states (DOSs) of BeAu. It is clearly seen that the a twofold IWP is localized at the high-symmetry point Γ (a red box), indicating the existence of IWP in this material.

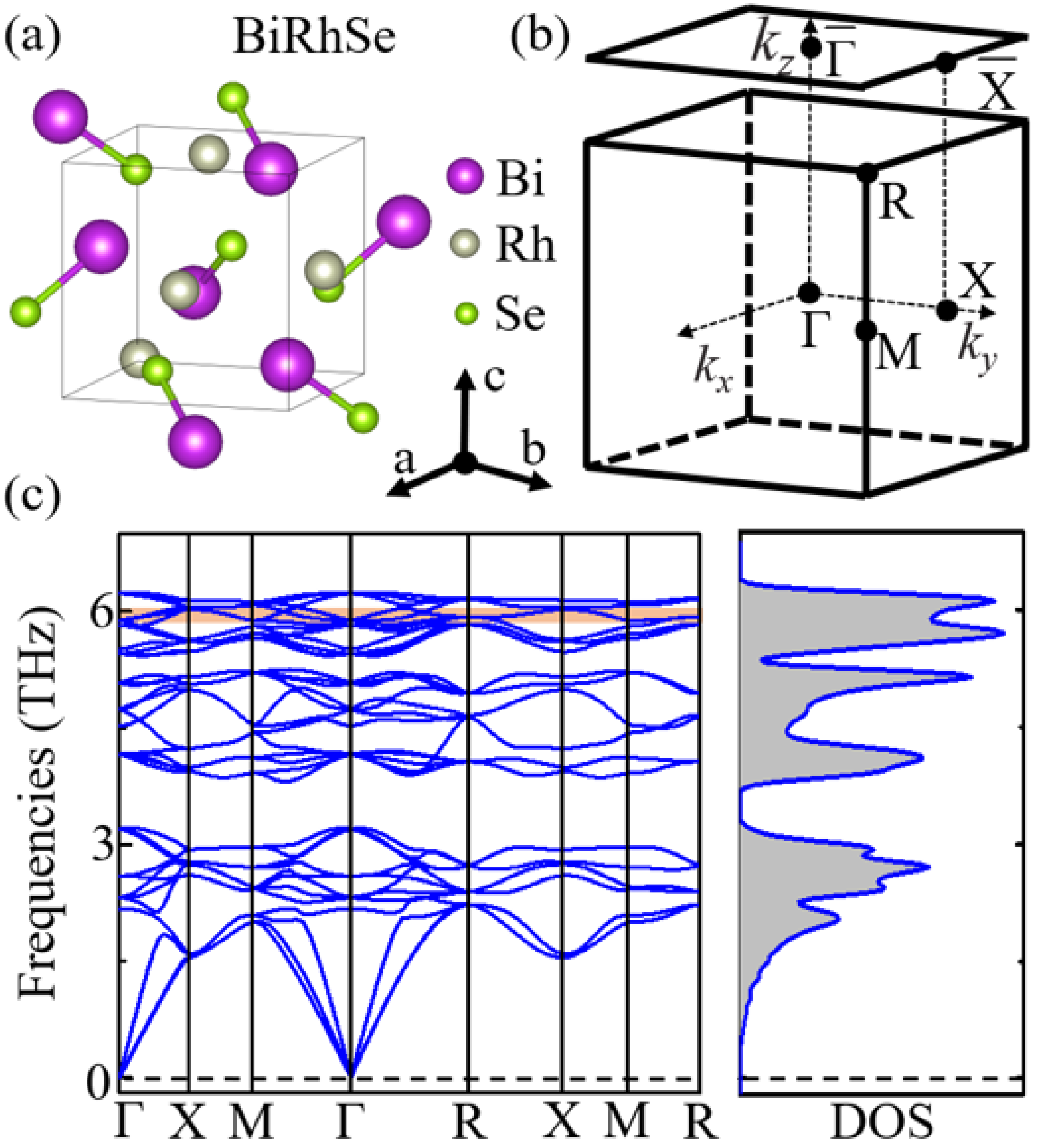

FIG. S20. A realistic candidate BiRhSe in SG 198. (a) A unit cell contains 4 Bi, 4 Rh and 4 atoms. (b) The first BZ of BiRhSe. (c) The phononic dispersions along the high-symmetry directions and phononic density of states (DOSs) of BiRhSe. It is clearly seen that the a twofold IWP is localized at the high-symmetry point Γ (a red box), indicating the existence of IWP in this material.

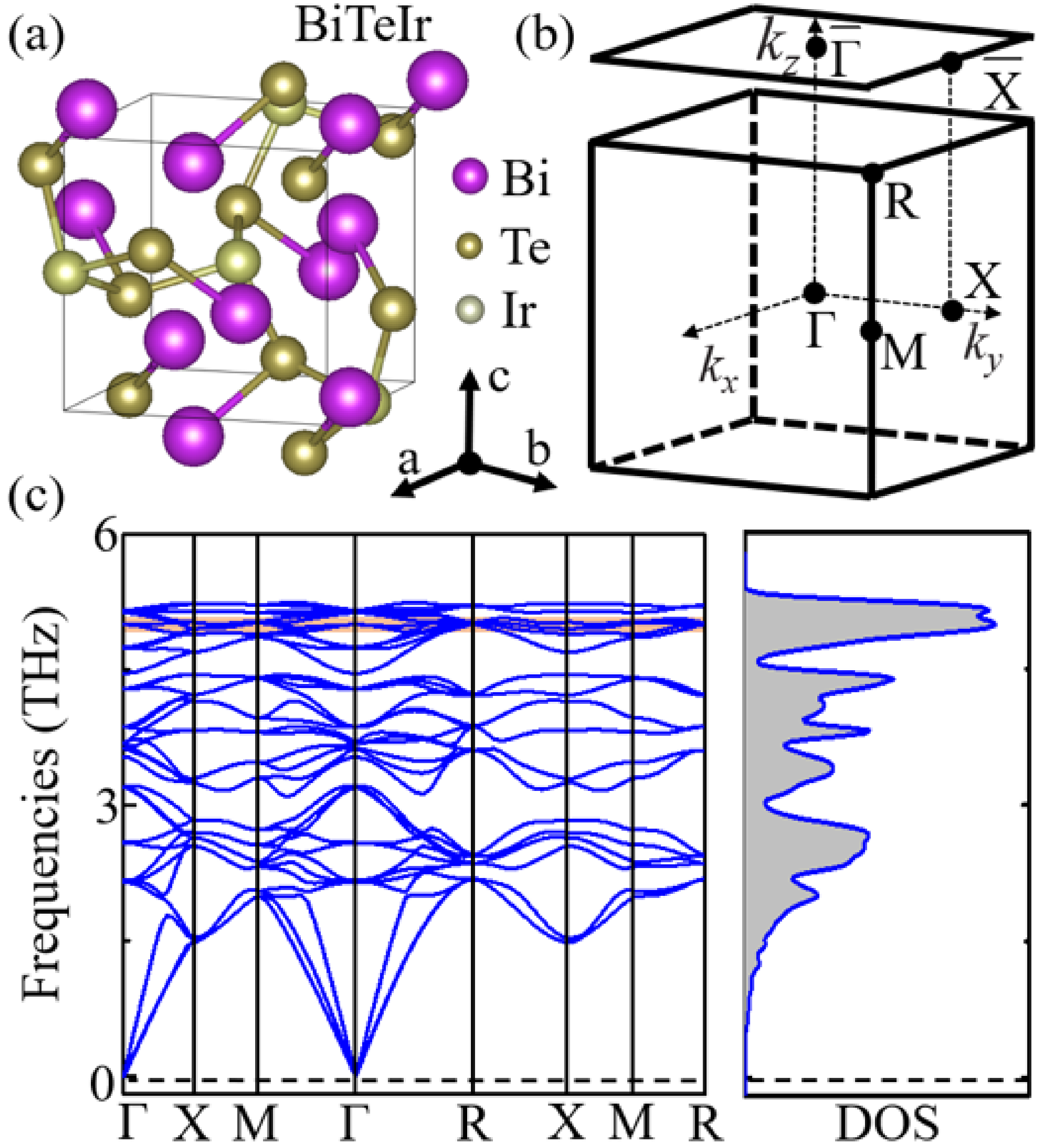

FIG. S21. A realistic candidate BiTeIr in SG 198. (a) A unit cell contains 4 Bi, 4 Te and 4 Ir atoms. (b) The first BZ of BiTeIr. (c) The phononic dispersions along the high-symmetry directions and phononic density of states (DOSs) of BiTeIr. It is clearly seen that the a twofold IWP is localized at the high-symmetry point Γ (a red box), indicating the existence of IWP in this material.

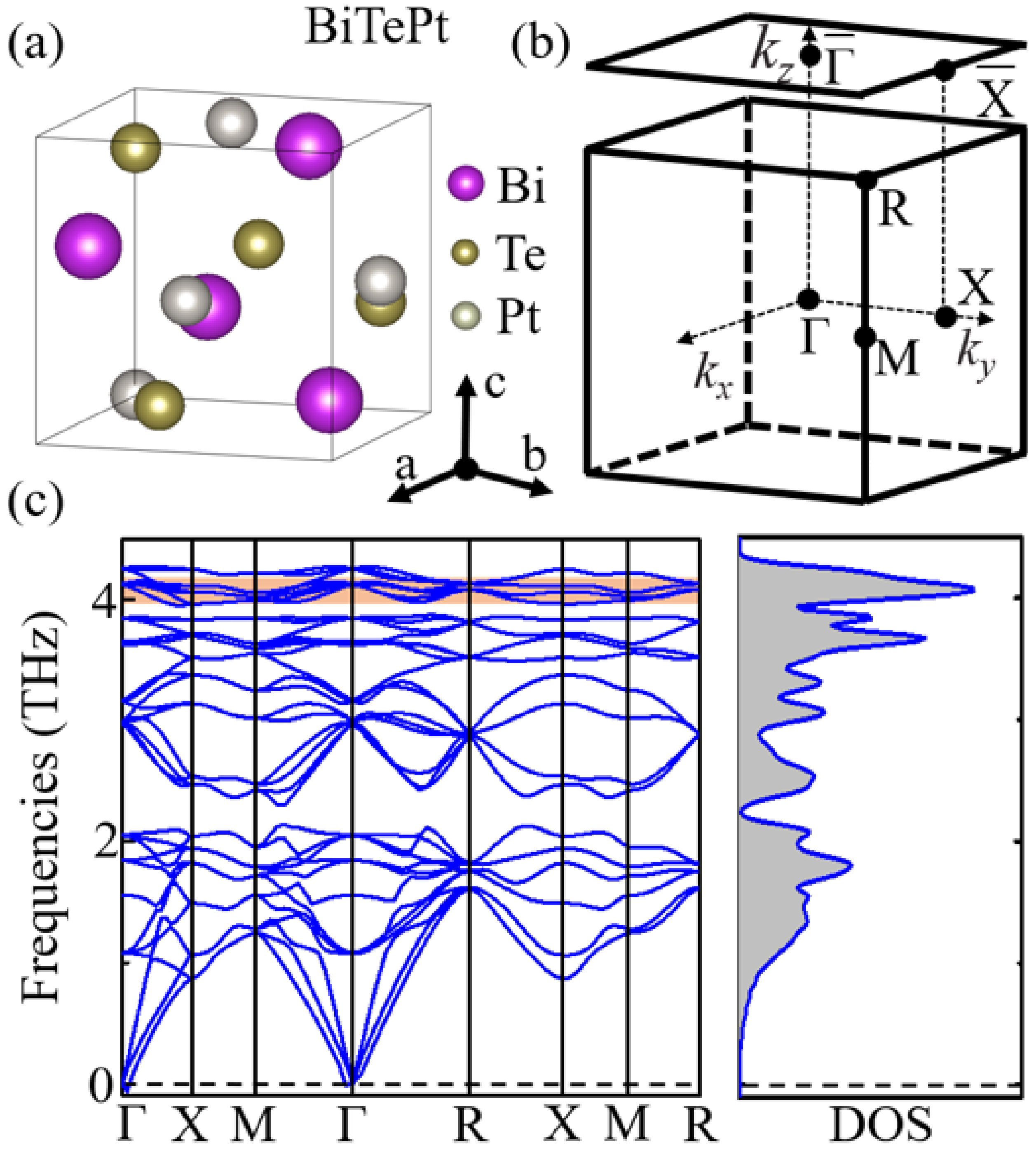

FIG. S22. A realistic candidate BiTePt in SG 198. (a) A unit cell contains 4 Bi, 4 Te and 4 Pt atoms. (b) The first BZ of BiTePt. (c) The phononic dispersions along the high-symmetry directions and the phononic density of states (DOSs) of BiTePt. It is clearly seen that the a twofold IWP is localized at the high-symmetry point Γ (a red box), indicating the existence of IWP in this material.

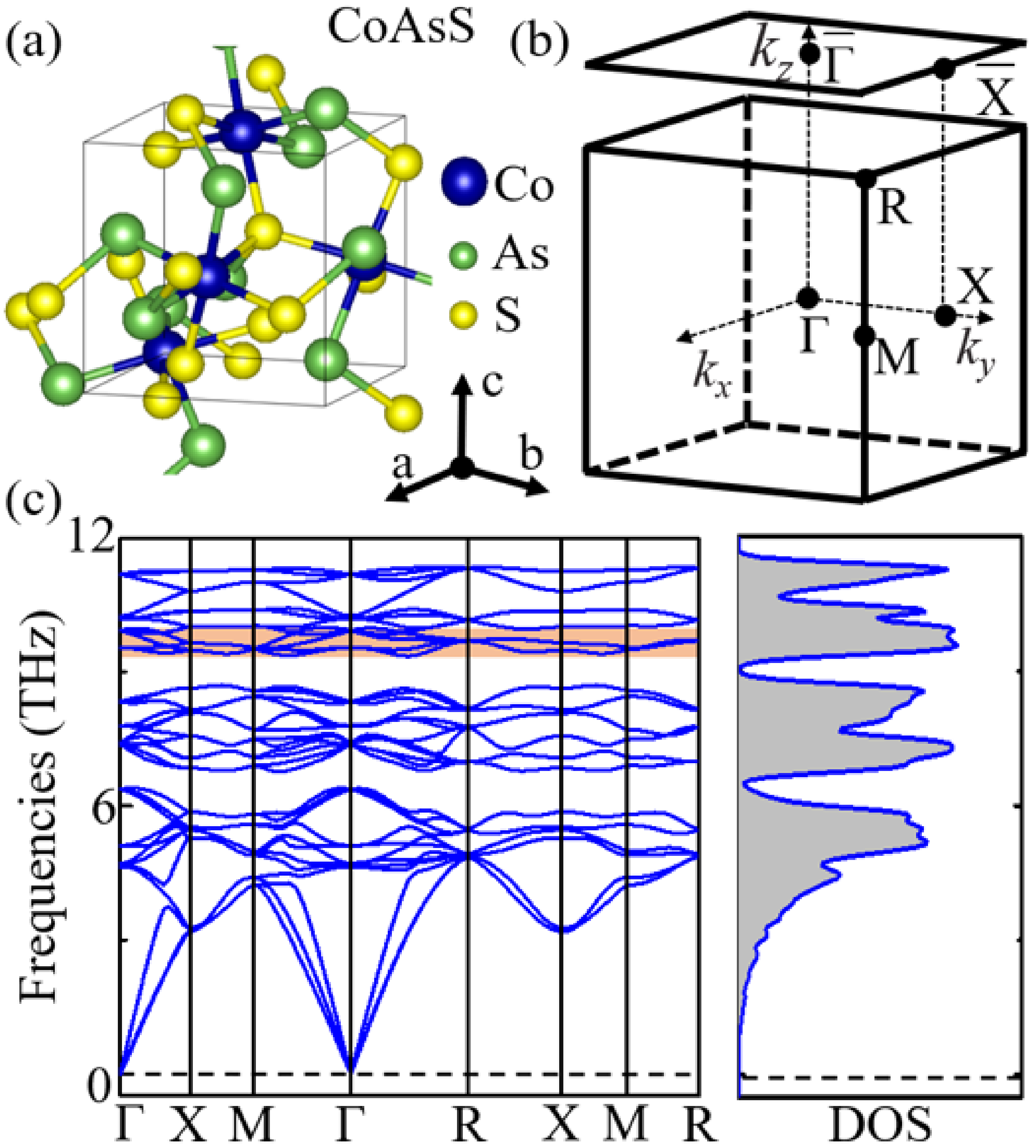

FIG. S23. A realistic candidate CoAsS in SG 198. (a) A unit cell contains 4 Co, 4 As and 4 S atoms. (b) The first BZ of CoAsS. (c) The phononic dispersions along the high-symmetry directions and the phononic density of states (DOSs) of CoAsS. It is clearly seen that the a twofold IWP is localized at the high-symmetry point Γ (a red box), indicating the existence of IWP in this material.

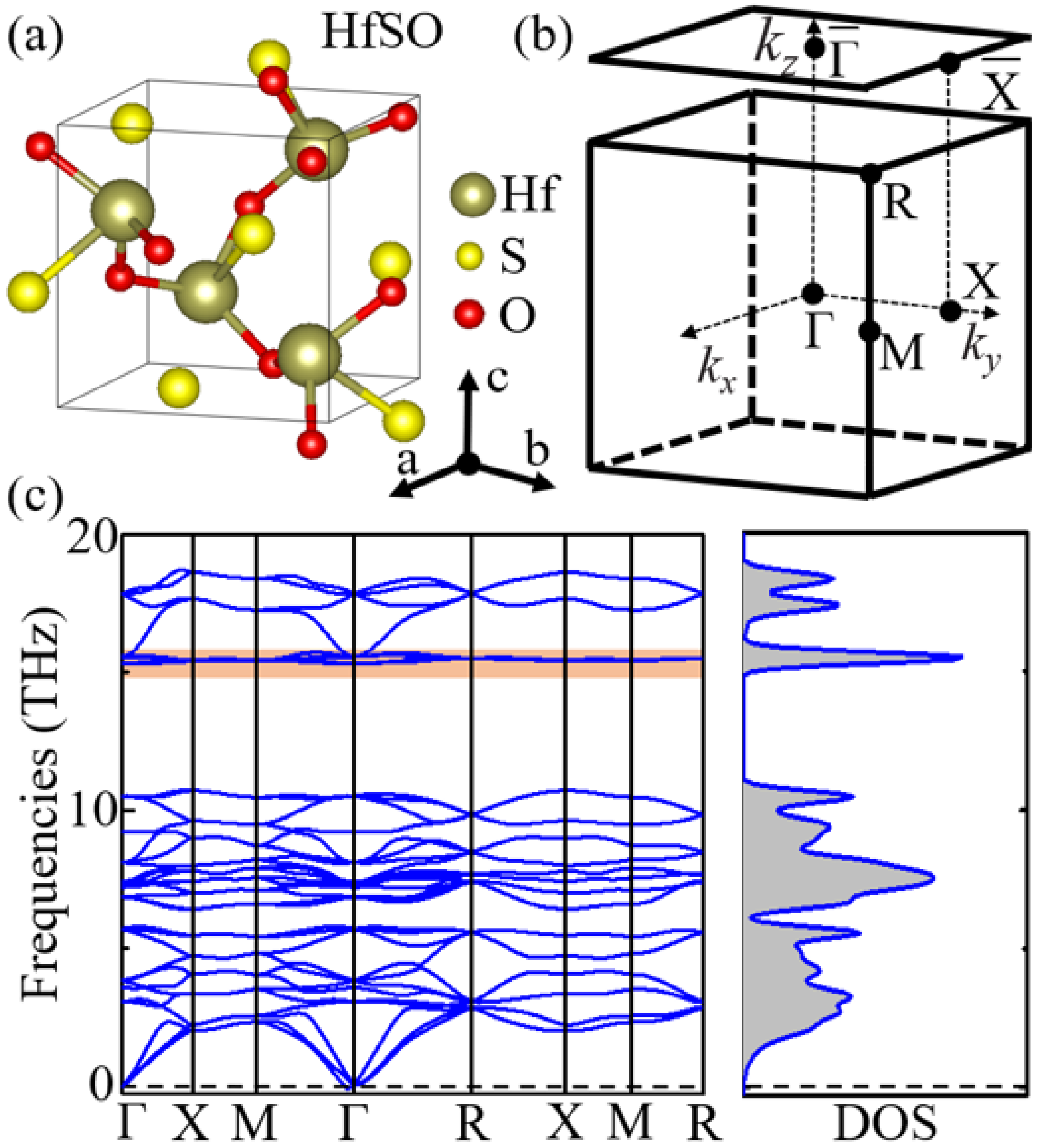

FIG. S24. A realistic candidate HfSO in SG 198. (a) A unit cell contains 4 Hf, 4 S and 4 O atoms. (b) The first BZ of HfSO. (c) The phononic dispersions along high-symmetry directions and phononic density of states (DOSs) of HfSO. It is clearly seen that the a twofold IWP is localized at the high-symmetry point Γ (a red box), indicating the existence of IWP in this material.

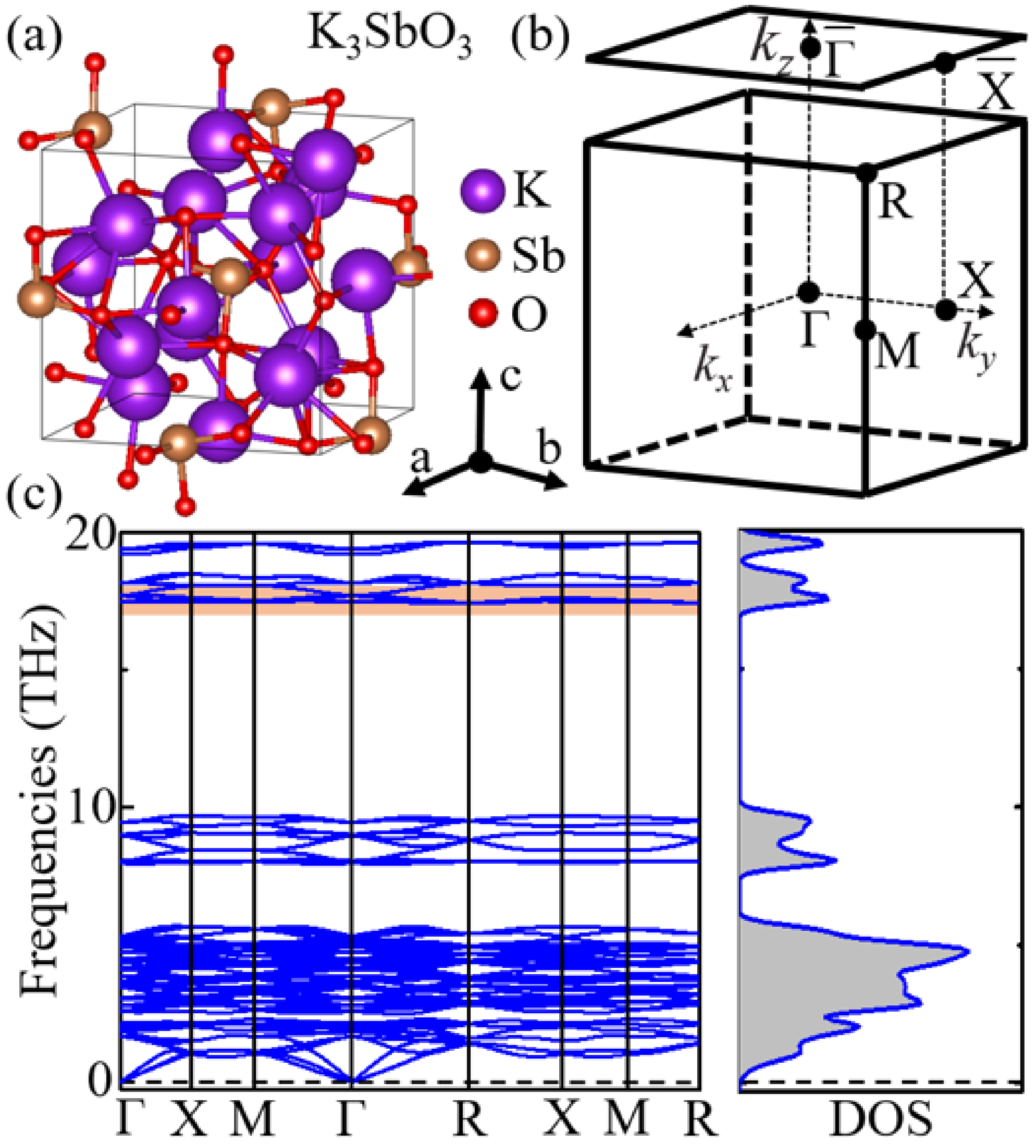

FIG. S25. A realistic candidate K_3SbO_3 in SG 198. (a) A unit cell contains 12 K, 4 Sb and 12 O atoms. (b) The first BZ of K_3SbO_3. (c) The phononic dispersions along the high-symmetry directions and phononic density of states (DOSs) of K_3SbO_3. It is clearly seen that the a twofold IWP is localized at the high-symmetry point Γ (a red box), indicating the existence of IWP in this material.

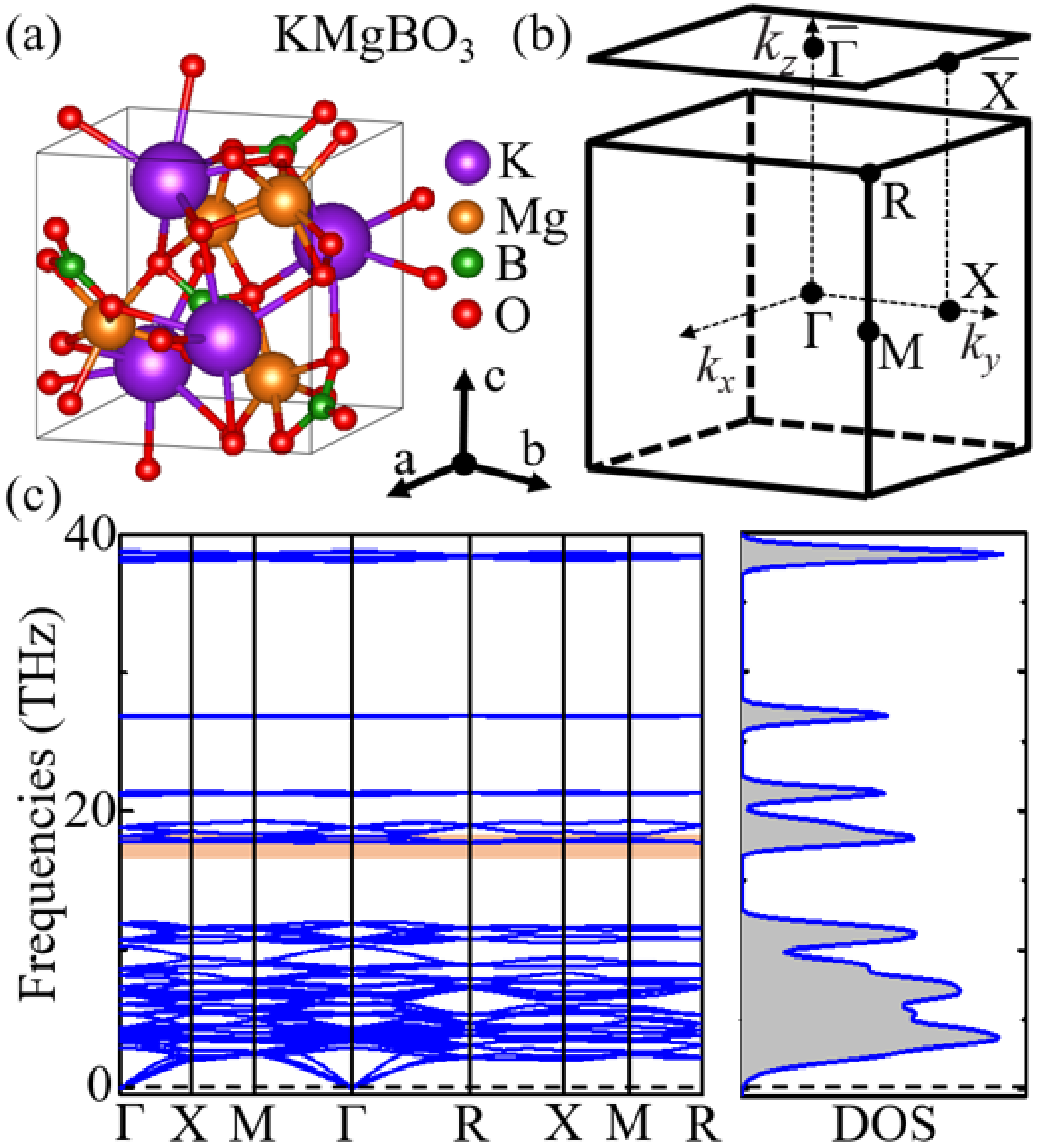

FIG. S26. A realistic candidate $KMgBO_3$ in SG 198. (a) A unit cell contains 4 K, 4 Mg, 4 B and 12 O atoms. (b) The first BZ of $KMgBO_3$. (c) The phononic dispersions along the high-symmetry directions and phononic density of states (DOSs) of $KMgBO_3$. It is clearly seen that the a twofold IWP is localized at the high-symmetry point Γ (a red box), indicating the existence of IWP in this material.

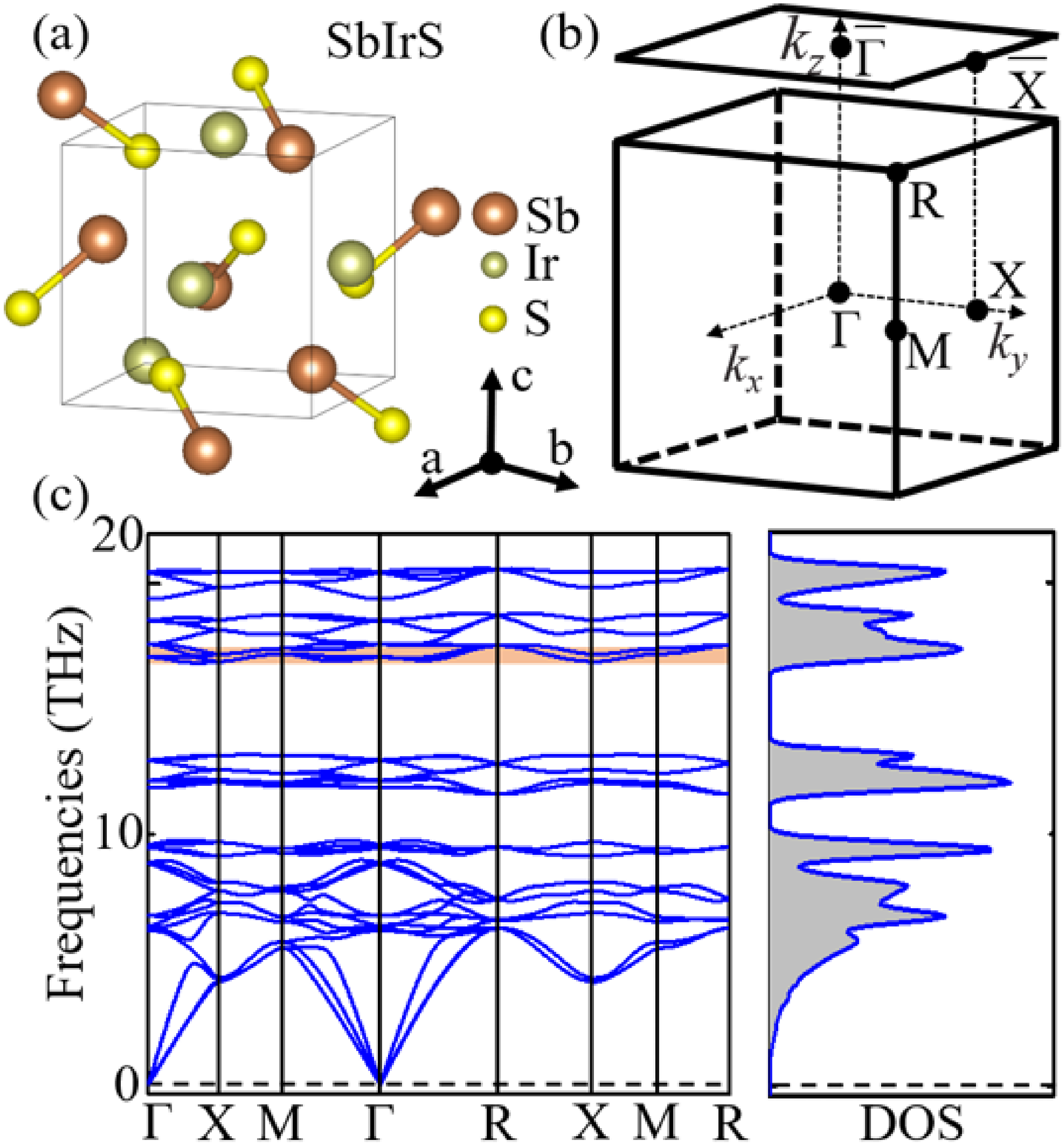

FIG. S27. A realistic candidate SbIrS in SG 198. (a) A unit cell contains 4 Sb, 4 Ir and 4 S atoms. (b) The first BZ of SbIrS. (c) The phononic dispersions along the high-symmetry directions and phononic density of states (DOSs) of SbIrS. It is clearly seen that the a twofold IWP is localized at the high-symmetry point Γ (a red box), indicating the existence of IWP in this material.

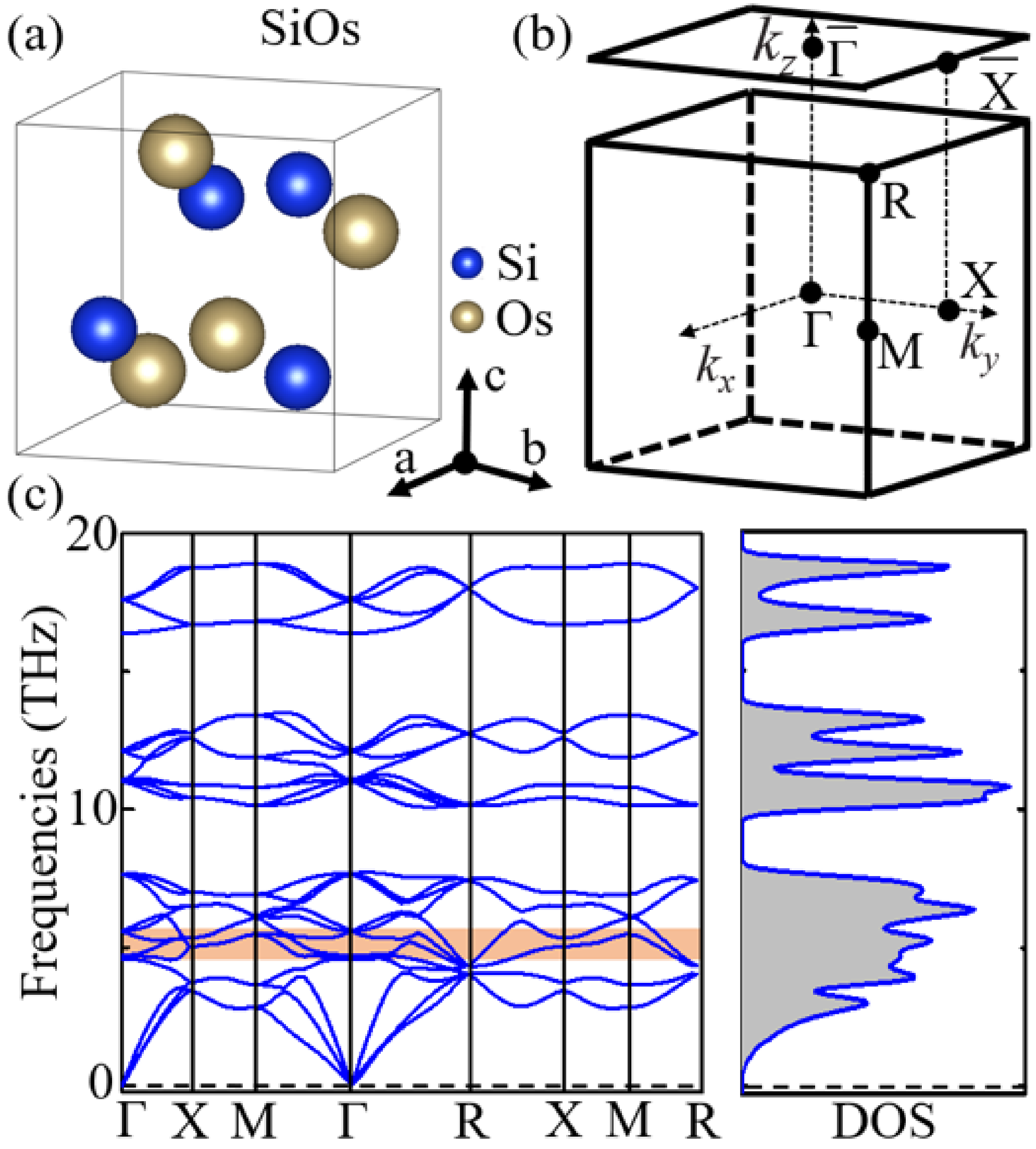

FIG. S28. A realistic candidate SiOs in SG 198. (a) A unit cell contains 4 Si and 4 Os atoms. (b) The first BZ of SiOs. (c) The phononic dispersions along the high-symmetry directions and the phononic density of states (DOSs) of SiOs. It is clearly seen that the a twofold IWP is localized at the high-symmetry point Γ (a red box), indicating the existence of IWP in this material.

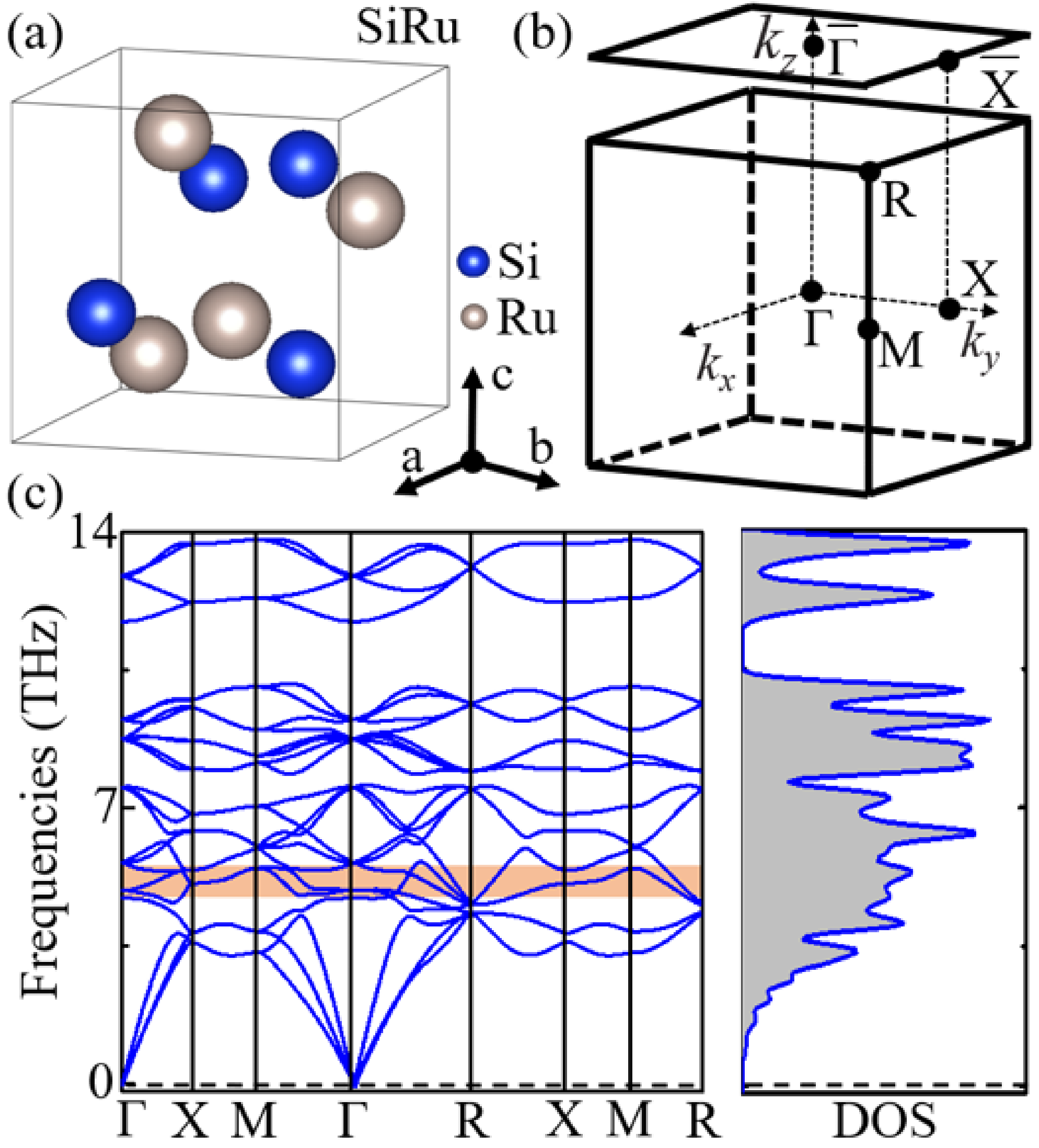

FIG. S29. A realistic candidate SiRu in SG 198. (a) A unit cell contains 4 Si and 4 Ru atoms. (b) The first BZ of SiRu. (c) The phononic dispersions along the high-symmetry directions and the phononic density of states (DOSs) of SiRu. It is clearly seen that the a twofold IWP is localized at the high-symmetry point Γ (a red box), indicating the existence of IWP in this material.

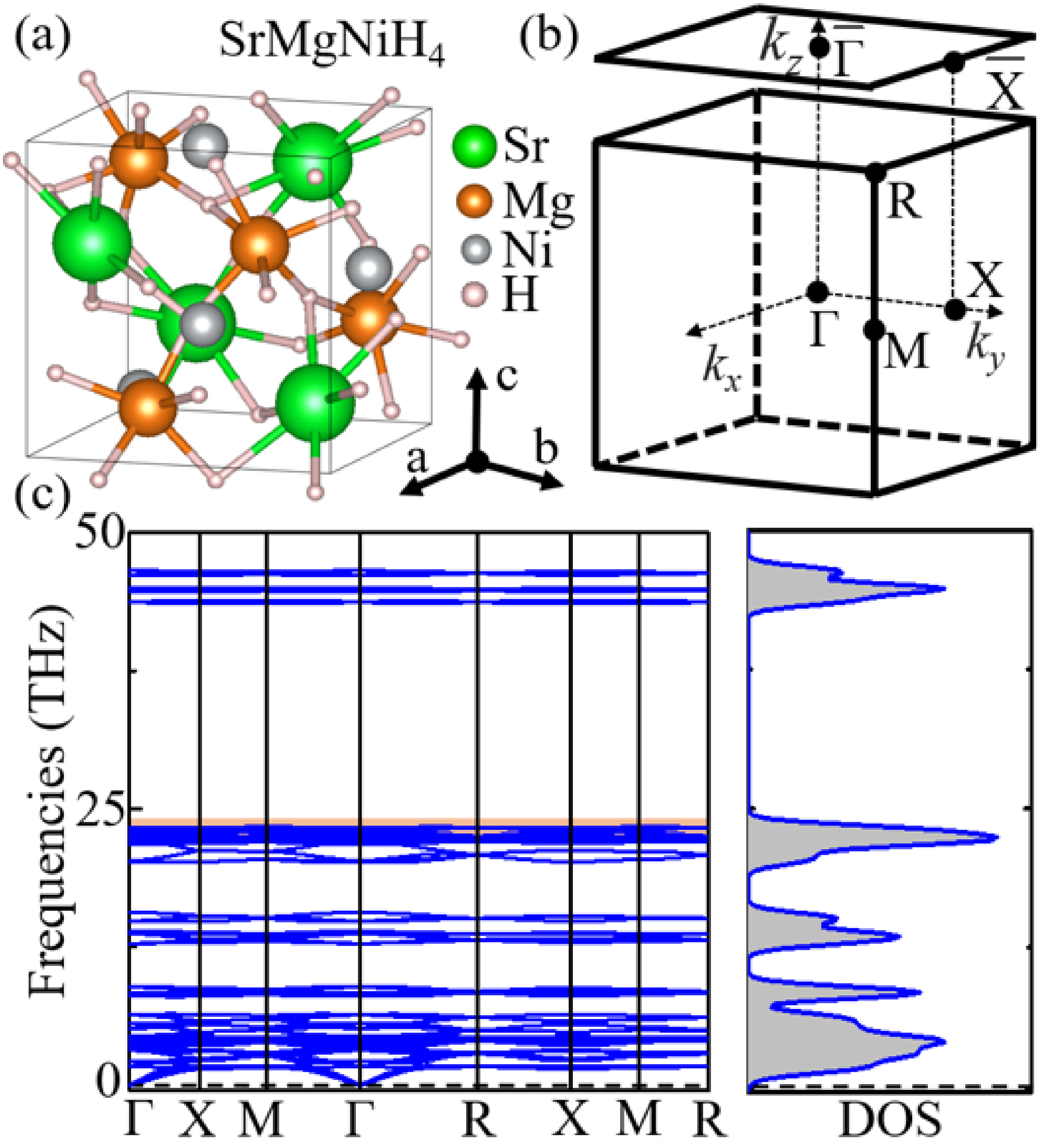

FIG. S30. A realistic candidate $SrMgNiH_4$ in SG 198. (a) A unit cell contains 4 Sr, 4 Mg, 4 Ni and 16 H atoms. (b) The first BZ of $SrMgNiH_4$. (c) The phononic dispersions along the high-symmetry directions and the phononic density of states (DOSs) of $SrMgNiH_4$. It is clearly seen that the a twofold IWP is localized at the high-symmetry point Γ (a red box), indicating the existence of IWP in this material.

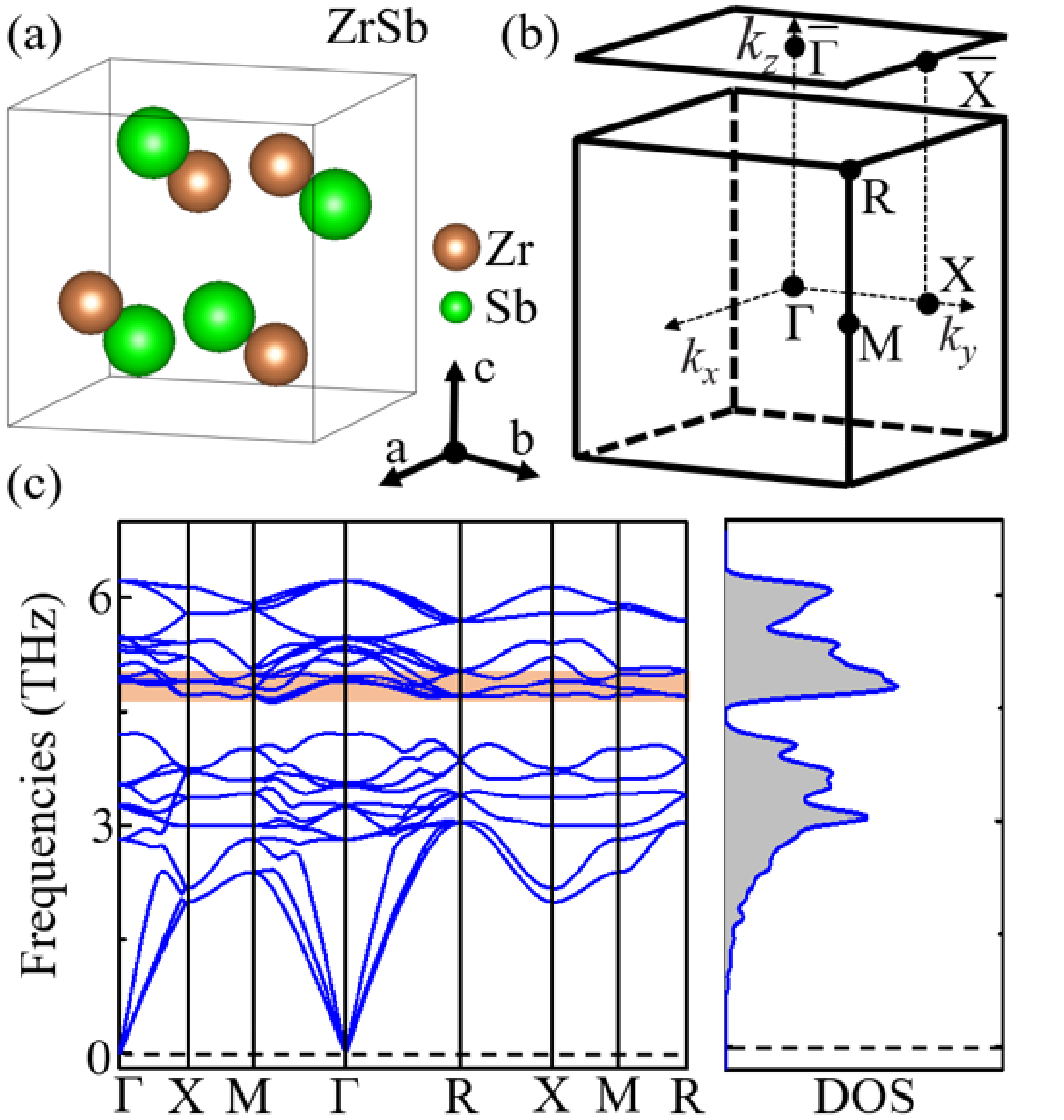

FIG. S31. A realistic candidate ZrSb in SG 198. (a) A unit cell contains 4 Zr and 4 Sb atoms. (b) The first BZ of ZrSb. (c) The phononic dispersions along the high-symmetry directions and the phononic density of states (DOSs) of ZrSb. It is clearly seen that the a twofold IWP is localized at the high-symmetry point Γ (a red box), indicating the existence of IWP in this material.

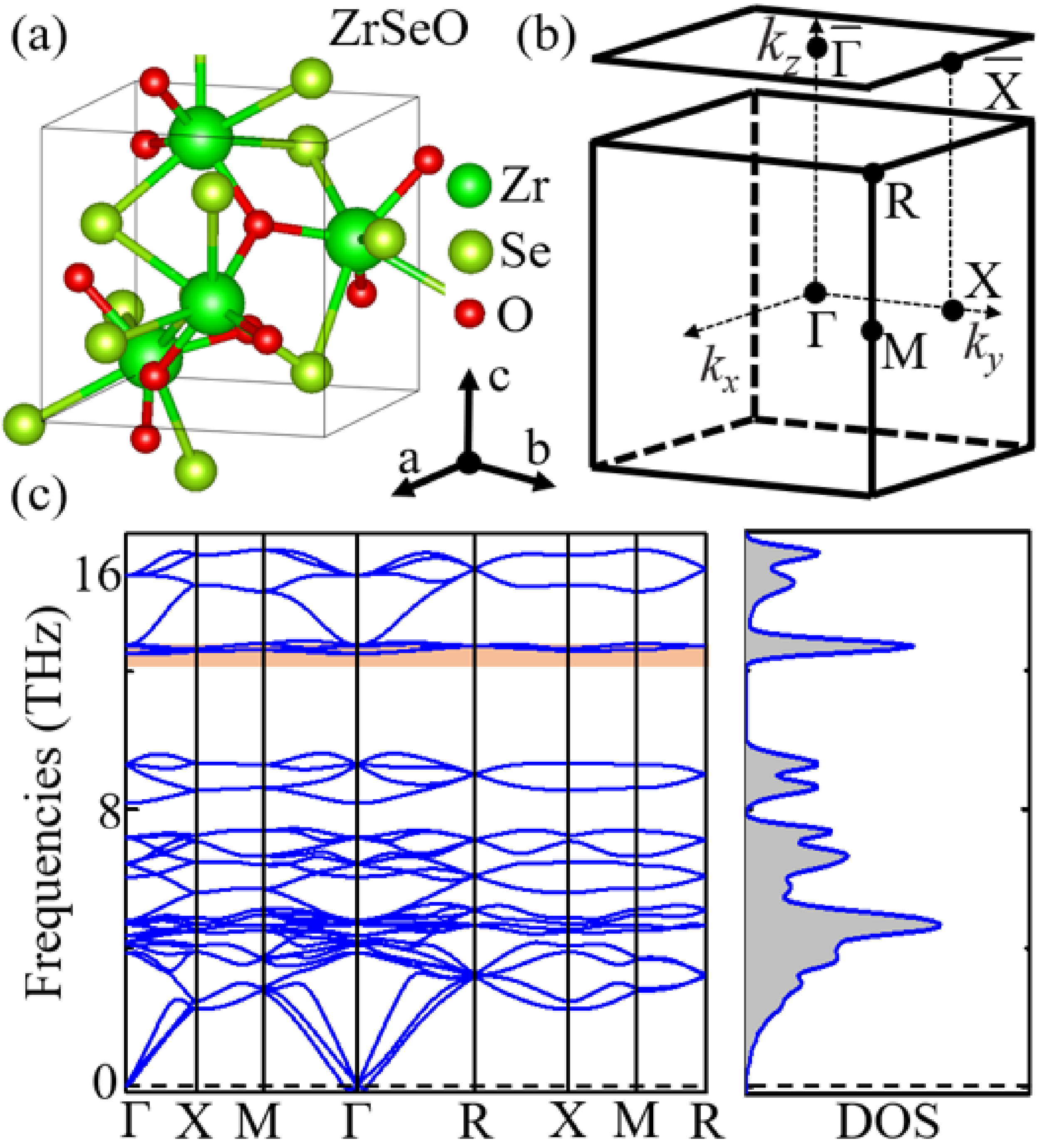

FIG. S32. A realistic candidate ZrSeO in SG 198. (a) A unit cell contains 4 Zr, 4 Se and 4 O atoms. (b) The first BZ of ZrSeO. (c) The phononic dispersions along the high-symmetry directions and the phononic density of states (DOSs) of ZrSeO. It is clearly seen that the a twofold IWP is localized at the high-symmetry point Γ (a red box), indicating the existence of IWP in this material.

F. IWPS WITH THE CHARGES OF ±4 IN SG 212 AND THE RELATED REALISTIC MATERIALS

In this section, we present 3 realistic materials candidates in SG 212 in Table I in the main context, to illustrate the existence of the IWPs with the charge of ±4 in realistic materials. We investigate the all thermodynamically stable materials: $BaGe_2$, $LiAl_5O_8$ and $SrSi_2$ in Materials Project (MP) [1]. Firstly, we draw their primitive unit cells and the first BZs to show the crystal structures. Then, we calculate their phononic dispersions in the first BZ to describe the existence of IWPs. The calculation methods are the same as those described in the main text. The crystallographics data of $BaGe_2$, $LiAl_5O_8$ and $SrSi_2$ are adopted from Ref. [1] and the primitive cells are shown in Fig. S33(a), S34(a) and S35(a), and the corresponding BZs are shown in Fig. S33(b), S34(b) and S35(b). To confirm the above results from the symmetry analysis, the phonon dispersions and the the twofold bands (red area) at the point Γ of $BaGe_2$, $LiAl_5O_8$ and $SrSi_2$ from the *ab initio* calculations [2] are illustrated in Fig. S33(c), S34(c) and S35(c).

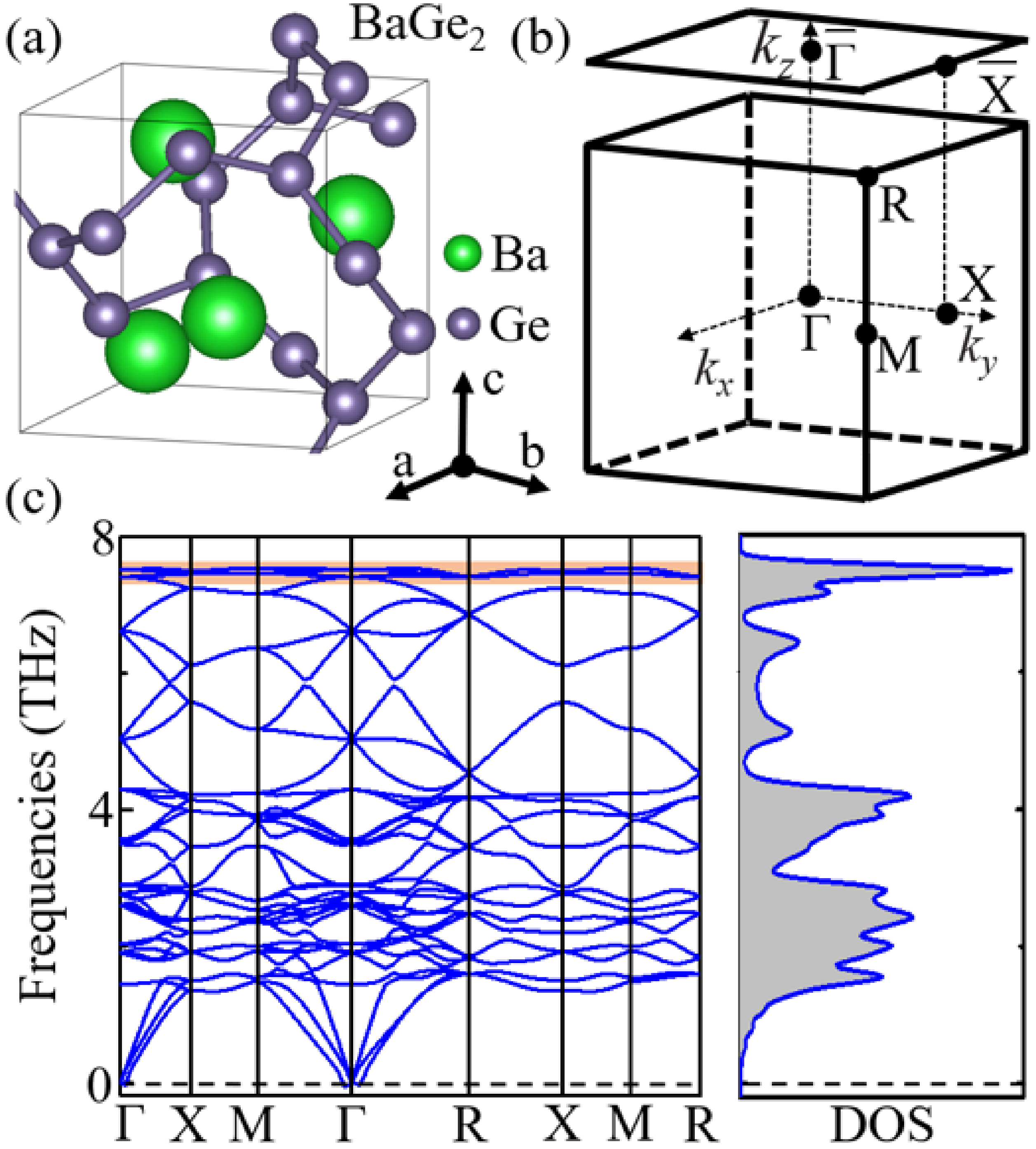

FIG. S33. A realistic candidate $BaGe_2$ in SG 212. (a) A unit cell contains 4 Ba and 8 Ge atoms. (b) The first BZ of $BaGe_2$. (c) The phononic dispersions along the high-symmetry directions and phononic density of states (DOSs) of $BaGe_2$. It is clearly seen that the a twofold IWP is localized at the high-symmetry point Γ (a red box), indicating the existence of IWP in this material.

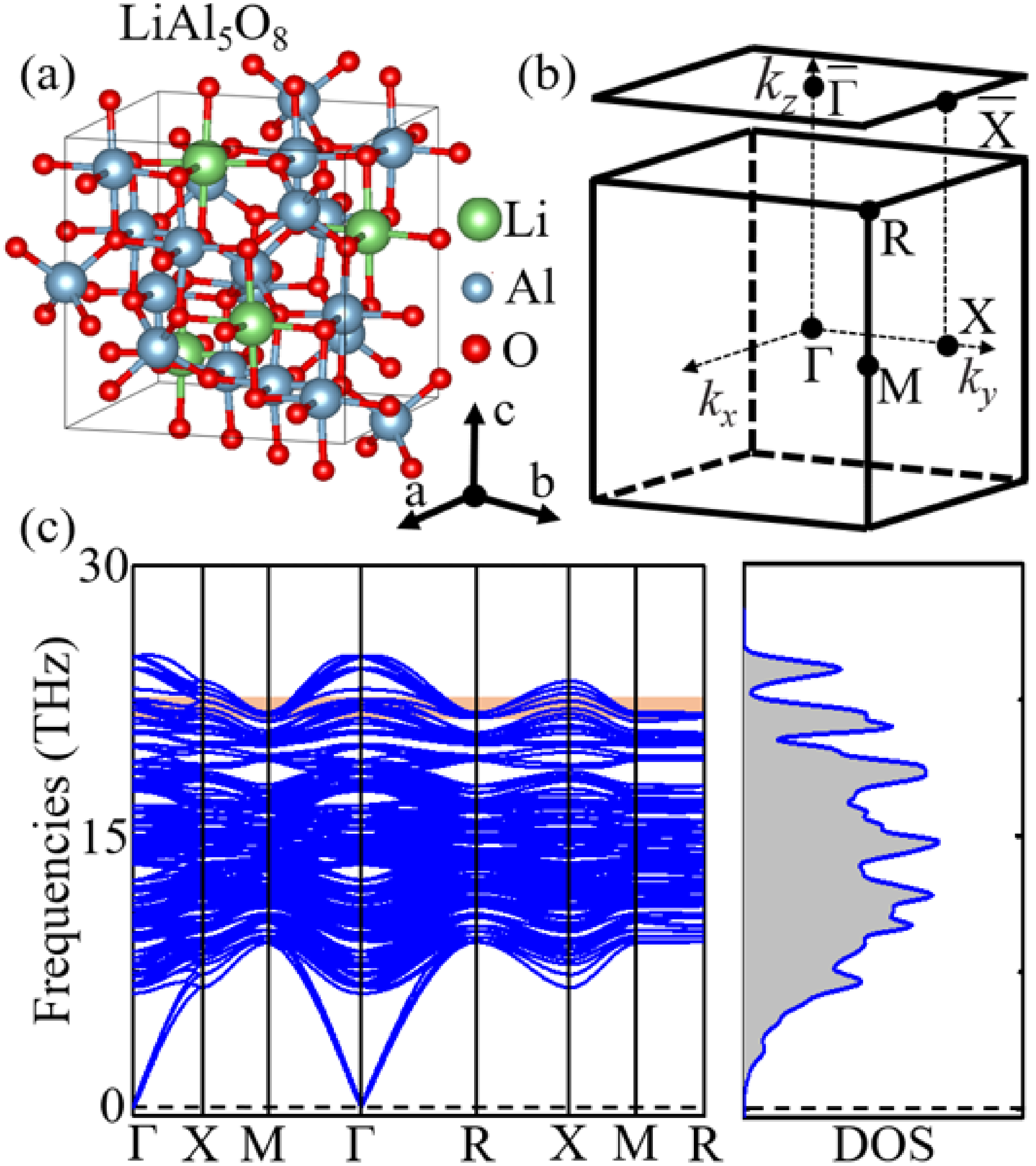

FIG. S34. A realistic candidate $LiAl_5O_8$ in SG 212. (a) A unit cell contains 4 Li, 20 Al and 32 O atoms. (b) The first BZ of $LiAl_5O_8$. (c) The phononic dispersions along the high-symmetry directions and the phononic density of states (DOSs) of $LiAl_5O_8$. It is clearly seen that the a twofold IWP is localized at the high-symmetry point Γ (a red box), indicating the existence of IWP in this material.

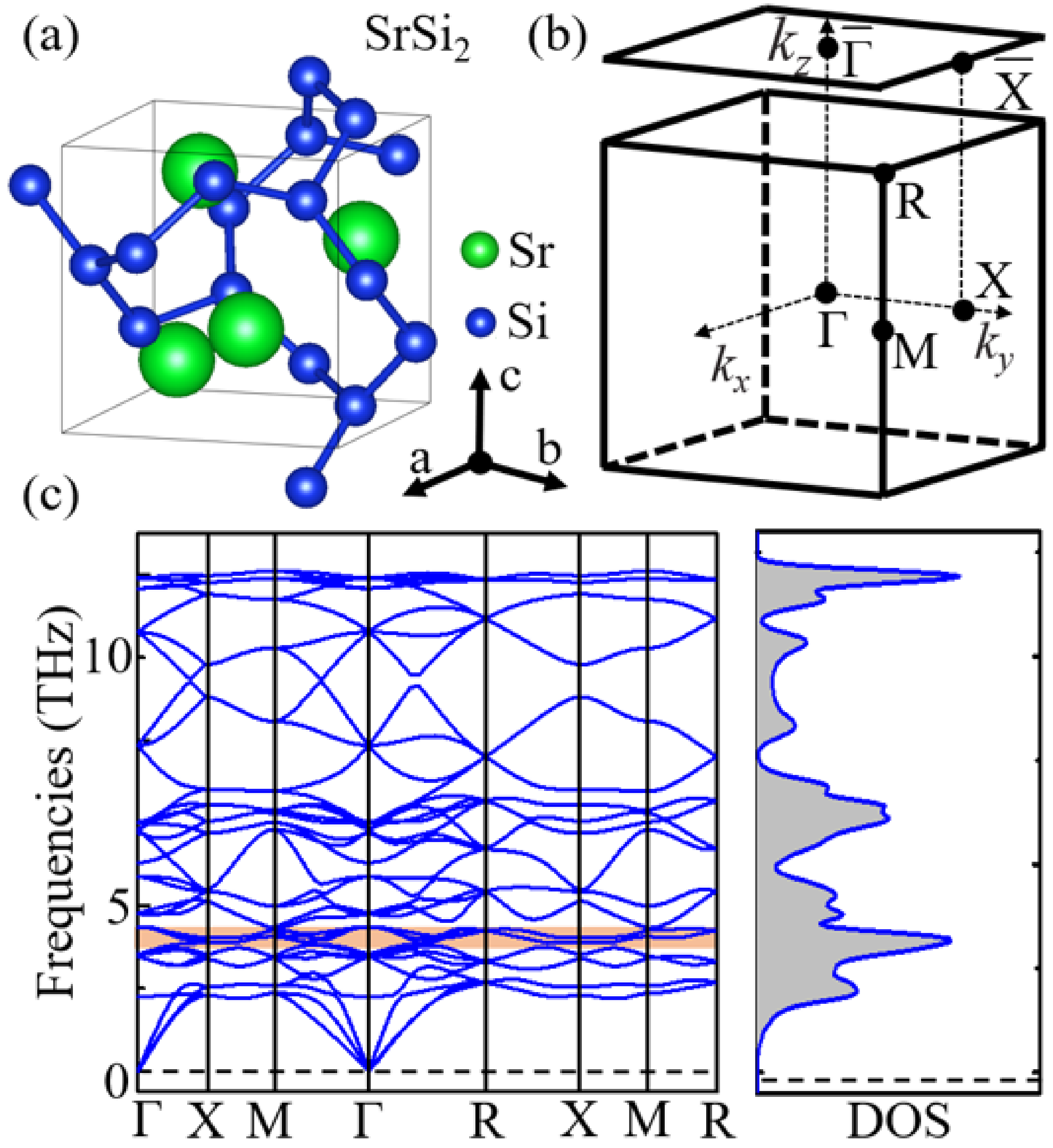

FIG. S35. A realistic candidate $SrSi_2$ in SG 212 (a) A unit cell contains 4 Sr and 8 Si atoms. (b) The first BZ of $SrSi_2$. (c) The phononic dispersions along the high-symmetry directions and phononic density of states (DOSs) of $SrSi_2$. It is clearly seen that the a twofold IWP is localized at the high-symmetry point Γ (a red box), indicating the existence of IWP in this material.

G. IWPS WITH THE CHARGE OF ±4 IN SG 213 AND THE RELATED REALISTIC MATERIALS

In this section, we present other 12 realistic materials candidates in SG 213 in Table I in the main context, to illustrate the existence of IWPs with the charge of ±4 in realistic materials. We investigate the all thermodynamically stable materials including $CsBe_2F_5$, Mg_3Ru_2, Mo_3Co_2N, Mo_3Ni_2N, Mo_3Pd_2N, $Na_4Sn_3O_8$, Nb_3Al_2C, Ta_3Al_2C, V_3Ga_2N, V_3Zn_2N, W_3Ni_2N and W_3Pd_2N in Materials Project (MP) [1]. Firstly, we draw their primitive unit cells and the first BZs to show the crystal structures. Then, we calculate their phononic dispersions in the first BZ to describe the existence of IWPs. The calculation methods are the same as those described in the main text. The crystallographics data of $CsBe_2F_5$, Mg_3Ru_2, Mo_3Co_2N, Mo_3Ni_2N, Mo_3Pd_2N, $Na_4Sn_3O_8$, Nb_3Al_2C, Ta_3Al_2C, V_3Ga_2N, V_3Zn_2N, W_3Ni_2N and W_3Pd_2N are adopted from Ref. [1] and the primitive cells are shown in Fig. S36(a), S37(a), S38(a), S39(a), S40(a), S41(a), S42(a), S43(a), S44(a), S45(a), S46(a) and S47(a), and the corresponding BZs are shown in Fig. S36(b), S37(b), S38(b), S39(b), S40(b), S41(b), S42(b), S43(b), S44(b), S45(b), S46(b), S47(b). To confirm the above results from the symmetry analysis, the phononic dispersions and the twofold bands (red area) at the point Γ of $CsBe_2F_5$, Mg_3Ru_2, Mo_3Co_2N, Mo_3Ni_2N, Mo_3Pd_2N, $Na_4Sn_3O_8$, Nb_3Al_2C, Ta_3Al_2C, V_3Ga_2N, V_3Zn_2N, W_3Ni_2N and W_3Pd_2N from the *ab initio* calculations [2] are illustrated in Fig. S36(c), S37(c), S38(c), S39(c), S40(c), S41(c), S42(c), S43(c), S44(c), S45(c), S46(c) and S47(c).

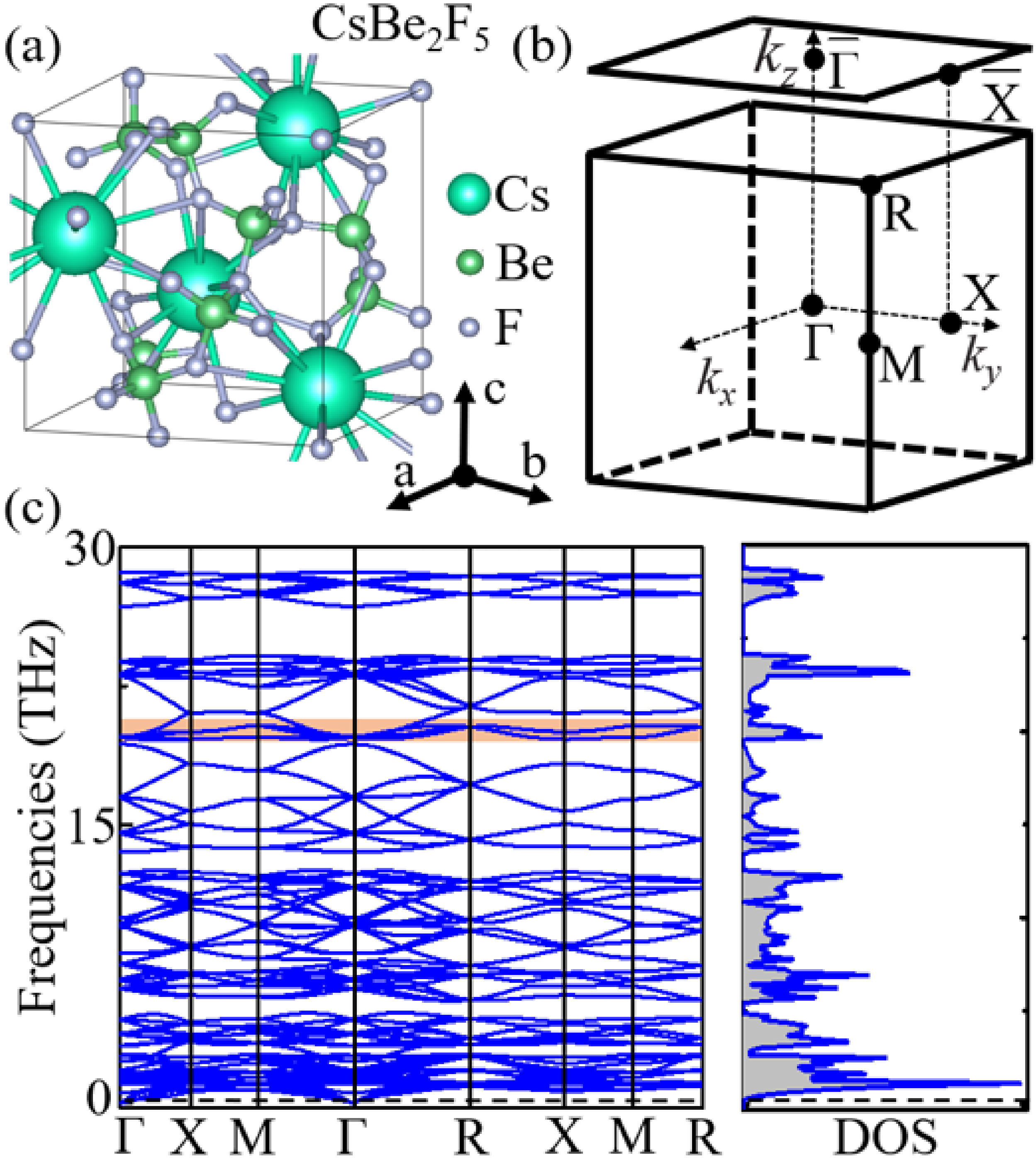

FIG. S36. A realistic candidate $CsBe_2F_5$ in SG 213. (a) A unit cell contains 4 Cs, 8 Be and 20 F atoms. (b) The first BZ of $CsBe_2F_5$. (c) The phononic dispersions along the high-symmetry directions and the phononic density of states (DOSs) of $CsBe_2F_5$. It is clearly seen that the a twofold IWP is localized at the high-symmetry point Γ (a red box), indicating the existence of IWP in this material.

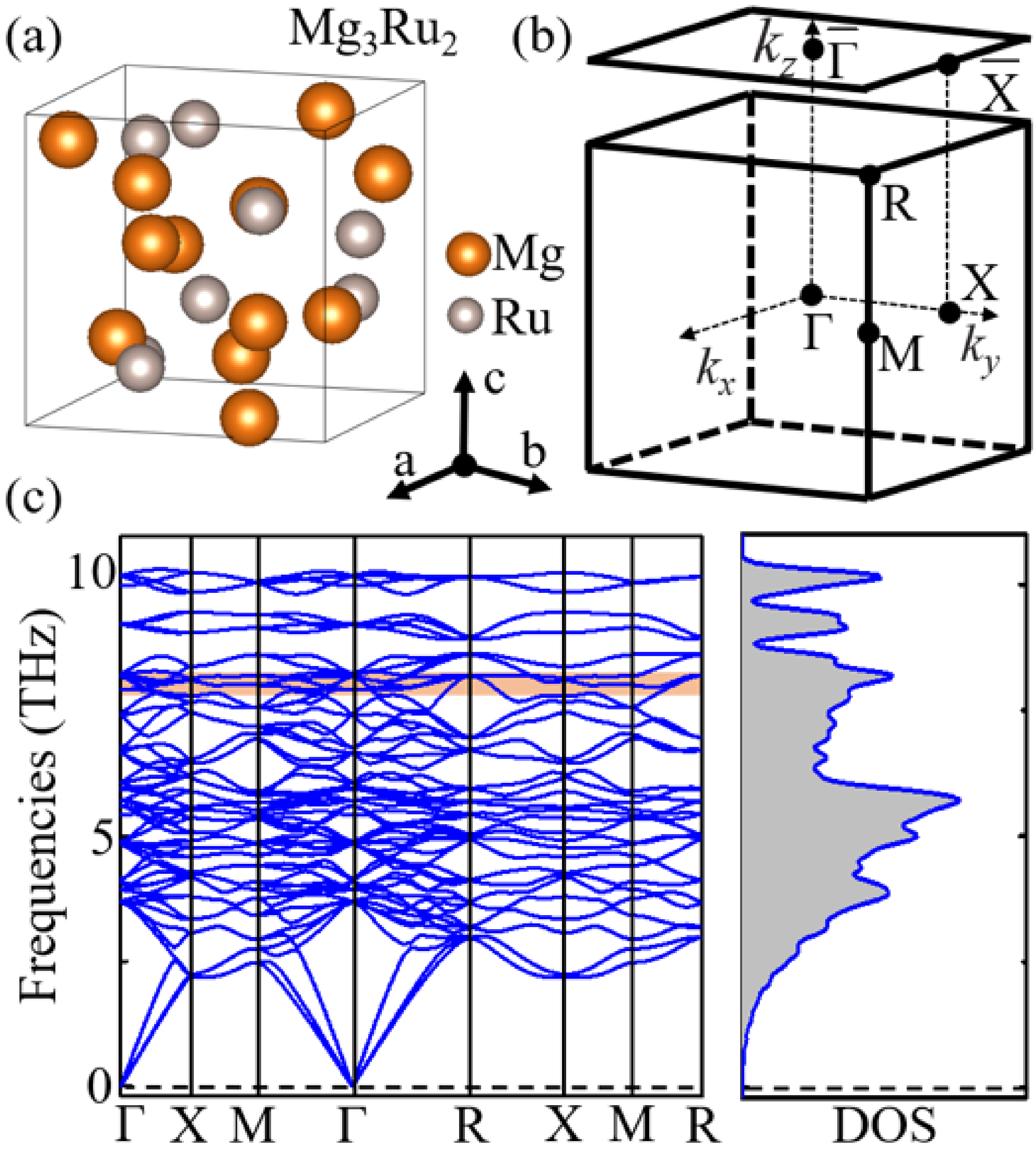

FIG. S37. A realistic candidate Mg_3Ru_2 in SG 213. (a) A unit cell contains 12 Mg and 8 Ru atoms. (b) The first BZ of Mg_3Ru_2. (c) The phonon dispersions along the high-symmetry directions and the phononic density of states (DOSs) of Mg_3Ru_2. It is clearly seen that the a twofold IWP is localized at the high-symmetry point Γ (a red box), indicating the existence of IWP in this material.

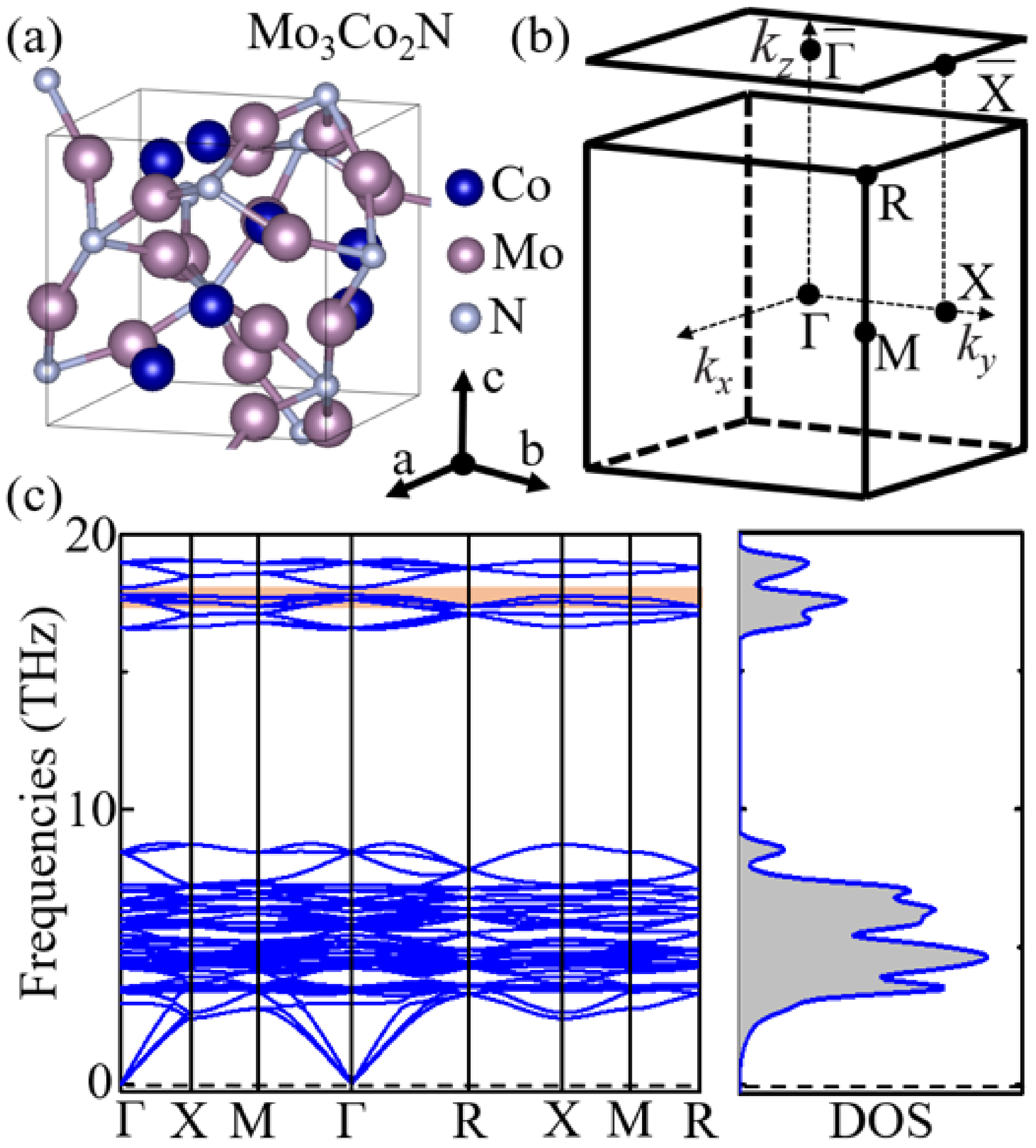

FIG. S38. A realistic candidate Mo_3Co_2N in SG 213. (a) A unit cell contains 12 Mo, 8 Co and 4 N atoms. (b) The first BZ of Mo_3Co_2N. (c) The phononic dispersions along the high-symmetry directions and the phononic density of states (DOSs) of Mo_3Co_2N. It is clearly seen that the a twofold IWP is localized at the high-symmetry point Γ (a red box), indicating the existence of IWP in this material.

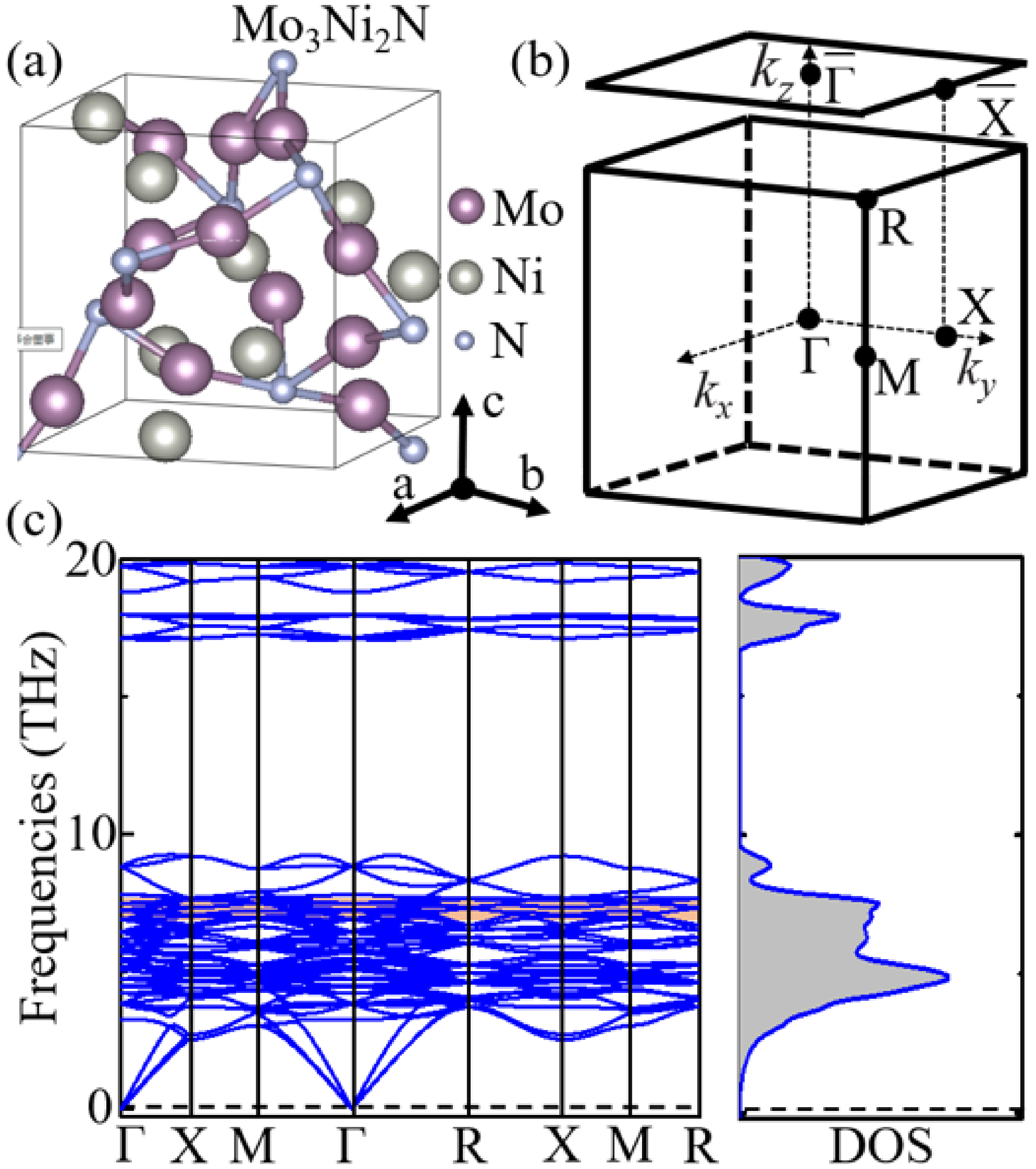

FIG. S39. A realistic candidate Mo_3Ni_2N in SG 213. (a) A unit cell contains 12 Mo, 8 Ni and 4 N atoms. (b) The first BZ of Mo_3Ni_2N. (c) The phononic dispersions along the high-symmetry directions and the phononic density of states (DOSs) of Mo_3Ni_2N. It is clearly seen that the a twofold IWP is localized at the high-symmetry point Γ (a red box), indicating the existence of IWP in this material.

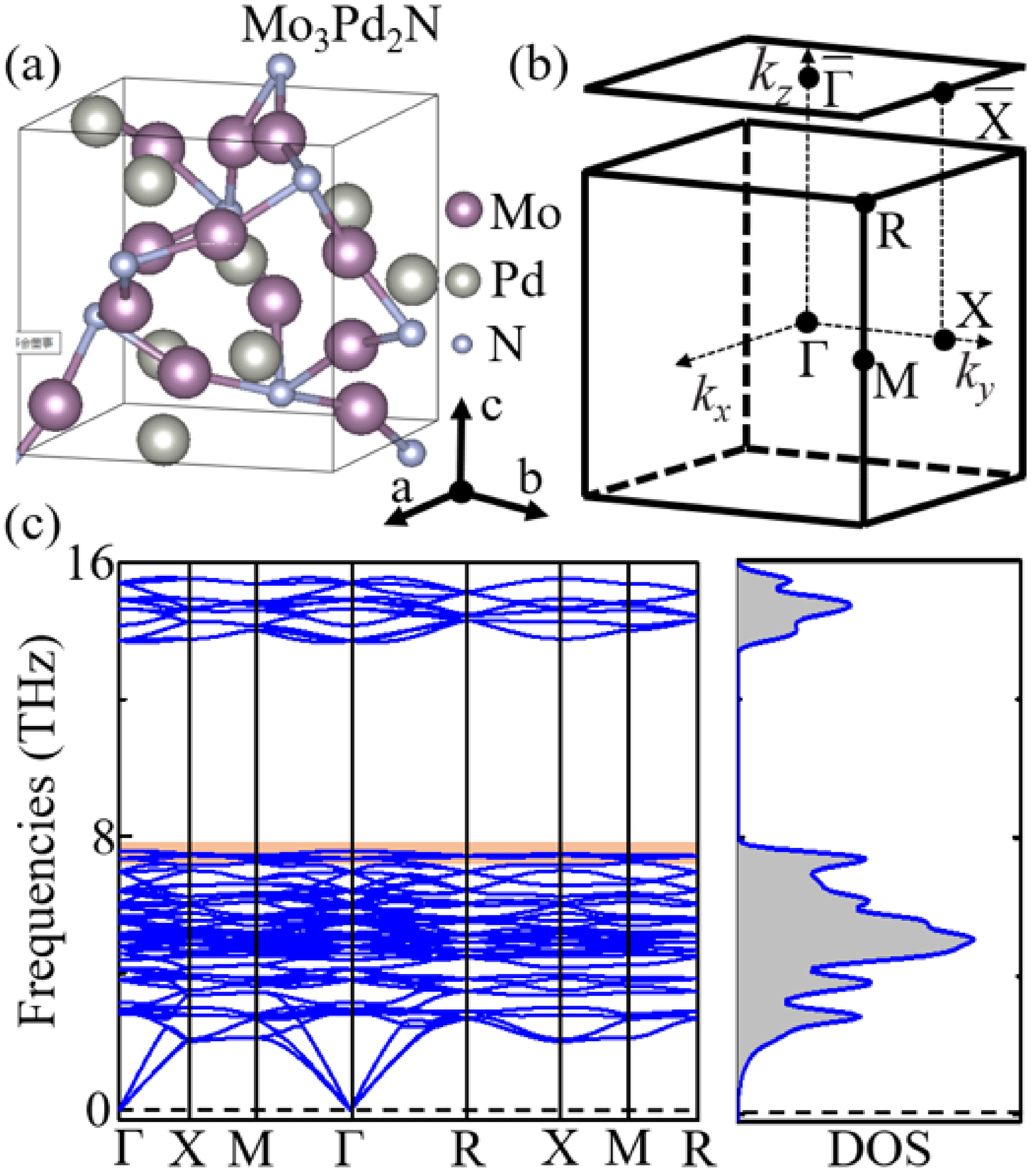

FIG. S40. A realistic candidate Mo_3Pd_2N in SG 213. (a) A unit cell contains 12 Mo, 8 Pd and 4 N atoms. (b) The first BZ of Mo_3Pd_2N. (c) The phononic dispersions along the high-symmetry directions and the phononic density of states (DOSs) of Mo_3Pd_2N. It is clearly seen that the a twofold IWP is localized at the high-symmetry point Γ (a red box), indicating the existence of IWP in this material.

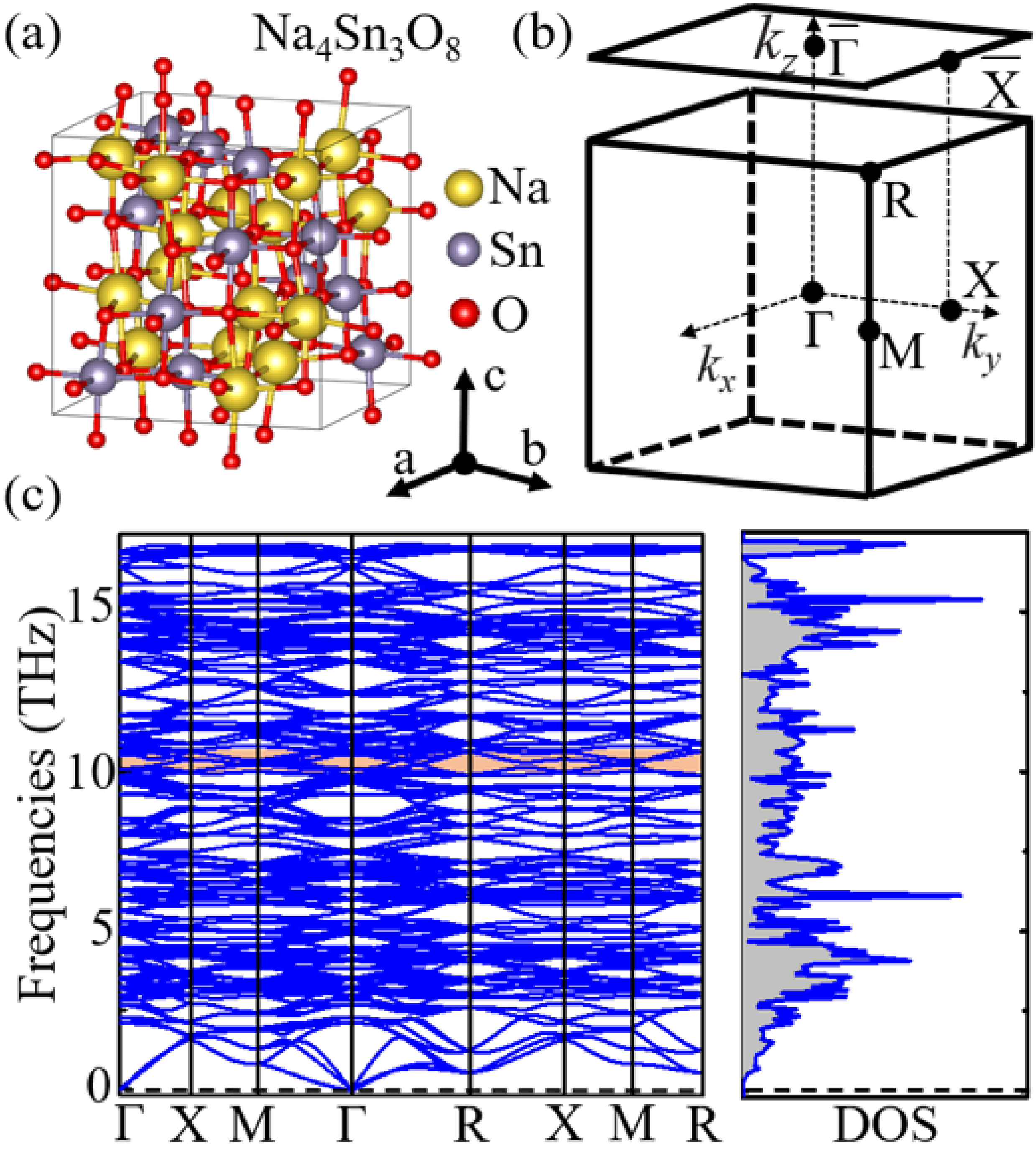

FIG. S41. A realistic candidate $Na_4Sn_3O_8$ in SG 213. (a) A unit cell contains 16 Na, 12 Sn and 32 O atoms. (b) The first BZ of $Na_4Sn_3O_8$. (c) The phononic dispersions along the high-symmetry directions and the phononic density of states (DOSs) of $Na_4Sn_3O_8$. It is clearly seen that the a twofold IWP is localized at the high-symmetry point Γ (a red box), indicating the existence of IWP in this material.

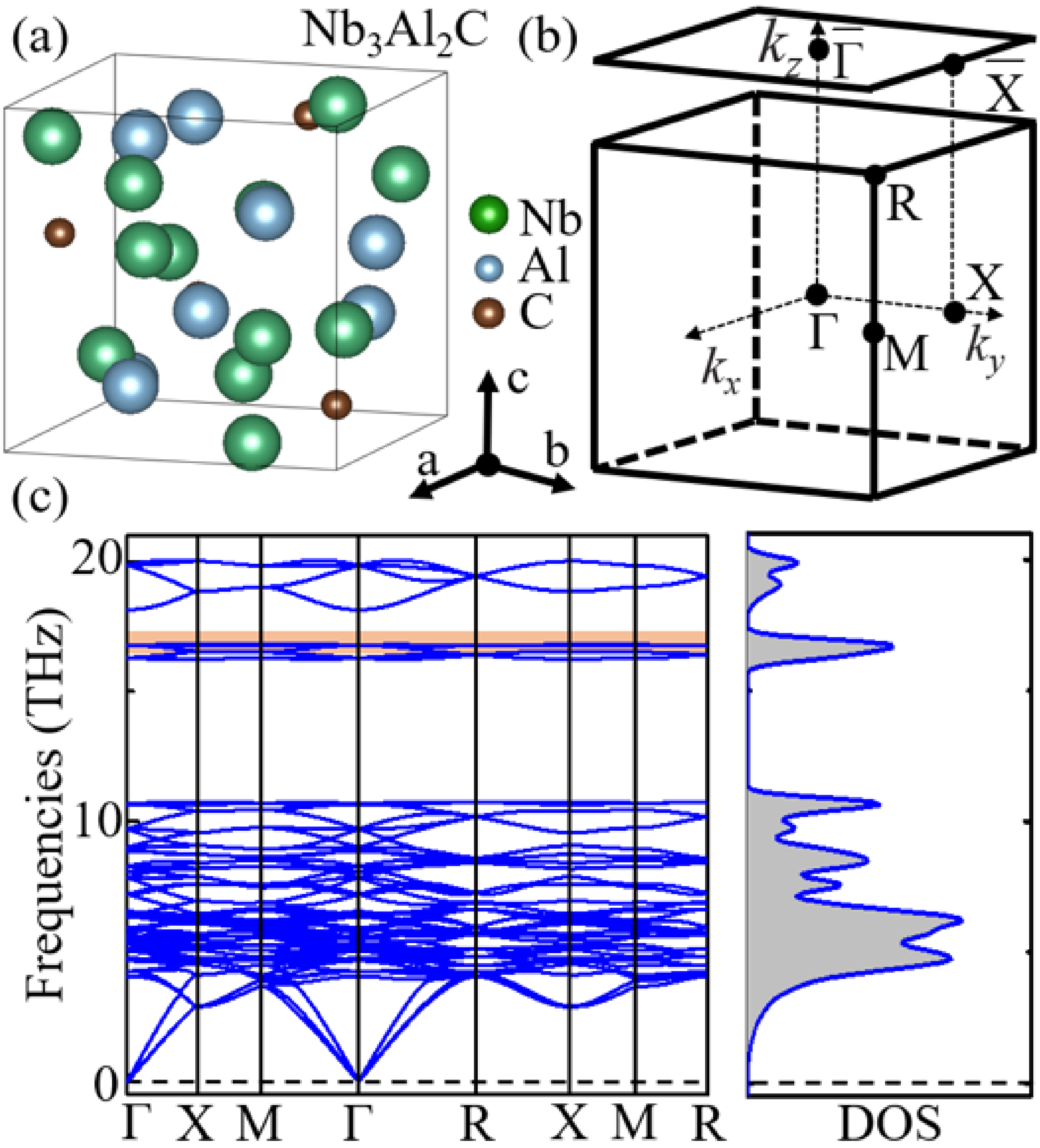

FIG. S42. A realistic candidate Nb_3Al_2C in SG 213. (a) A unit cell contains 12 Nb, 8 Al and 4 C atoms. (b) The first BZ of Nb_3Al_2C. (c) The phononic dispersions along the high-symmetry directions and the phonon density of states (DOSs) of Nb_3Al_2C. It is clearly seen that the a twofold IWP is localized at the high-symmetry point Γ (a red box), indicating the existence of IWP in this material.

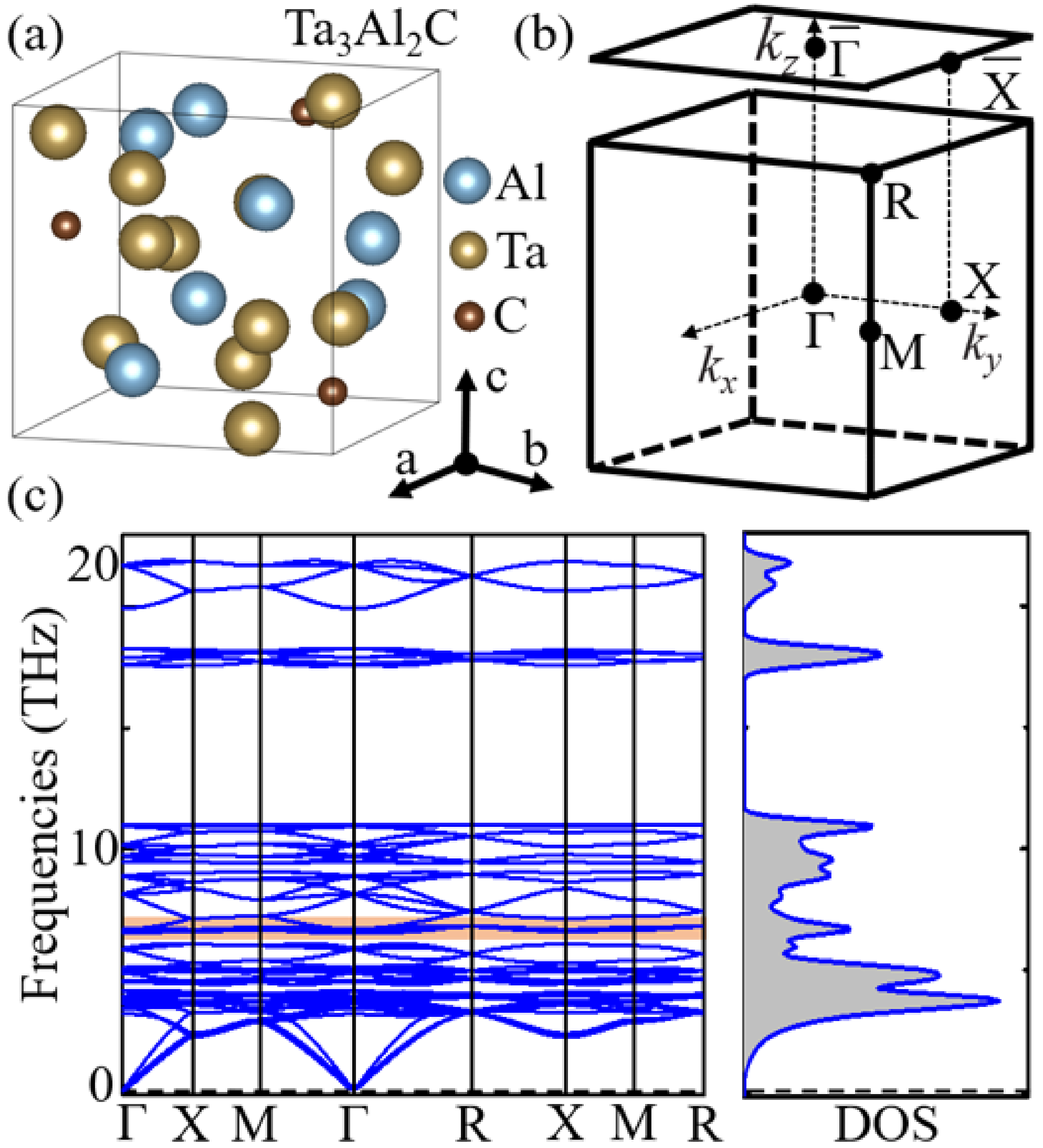

FIG. S43. A realistic candidate Ta_3Al_2C in SG 213. (a) A unit cell contains 12 Ta, 8 Al and 4 C atoms. (b) The first BZ of Ta_3Al_2C. (c) The phononic dispersions along the high-symmetry directions and the phononic density of states (DOSs) of Ta_3Al_2C. It is clearly seen that the a twofold IWP is localized at the high-symmetry point Γ (a red box), indicating the existence of IWP in this material.

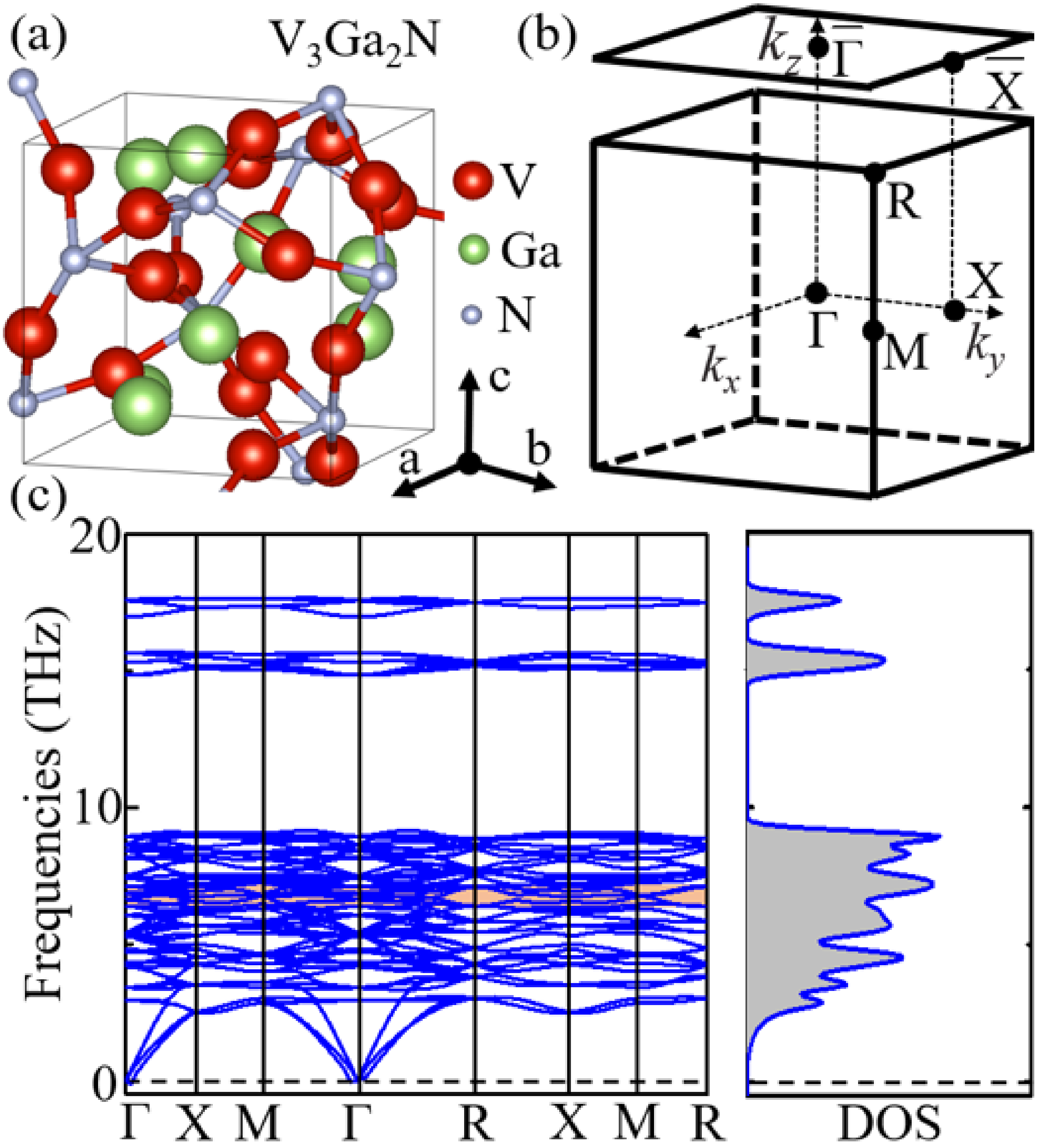

FIG. S44. A realistic candidate V_3Ga_2N in SG 213. (a) A unit cell contains 12 V, 8 Al and 4 N atoms. (b) The first BZ of V_3Ga_2N. (c) The phononic dispersions along the high-symmetry directions and the phononic density of states (DOSs) of V_3Ga_2N. It is clearly seen that the a twofold IWP is localized at the high-symmetry point Γ (a red box), indicating the existence of IWP in this material.

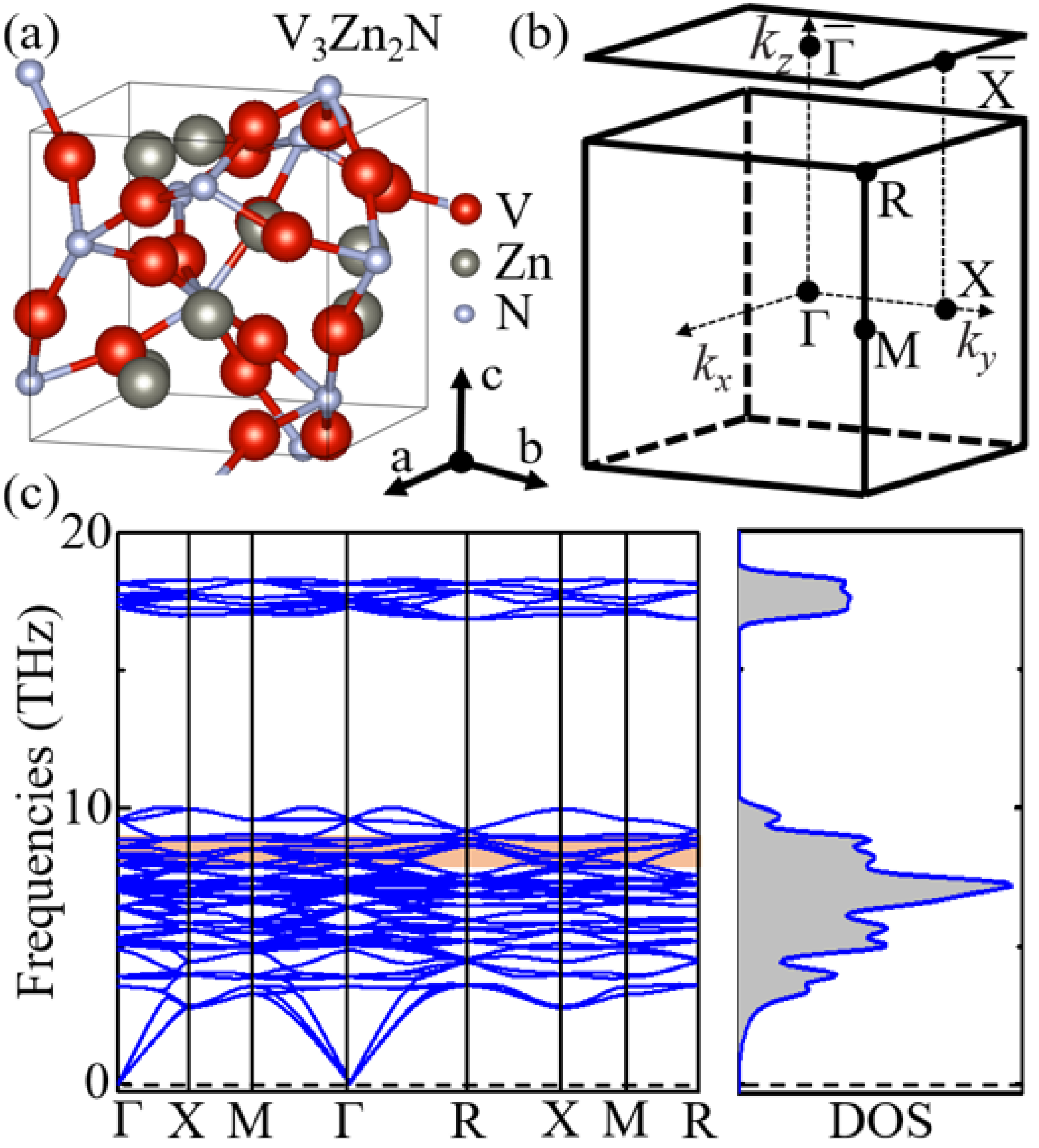

FIG. S45. A realistic candidate V_3Zn_2N in SG 213. (a) A unit cell contains 12 V, 8 Zn and 4 N atoms. (b) The first BZ of V_3Ga_2N. (c) The phononic dispersions along the high-symmetry directions and the phononic density of states (DOSs) of V_3Zn_2N. It is clearly seen that the a twofold IWP is localized at the high-symmetry point Γ (a red box), indicating the existence of IWP in this material.

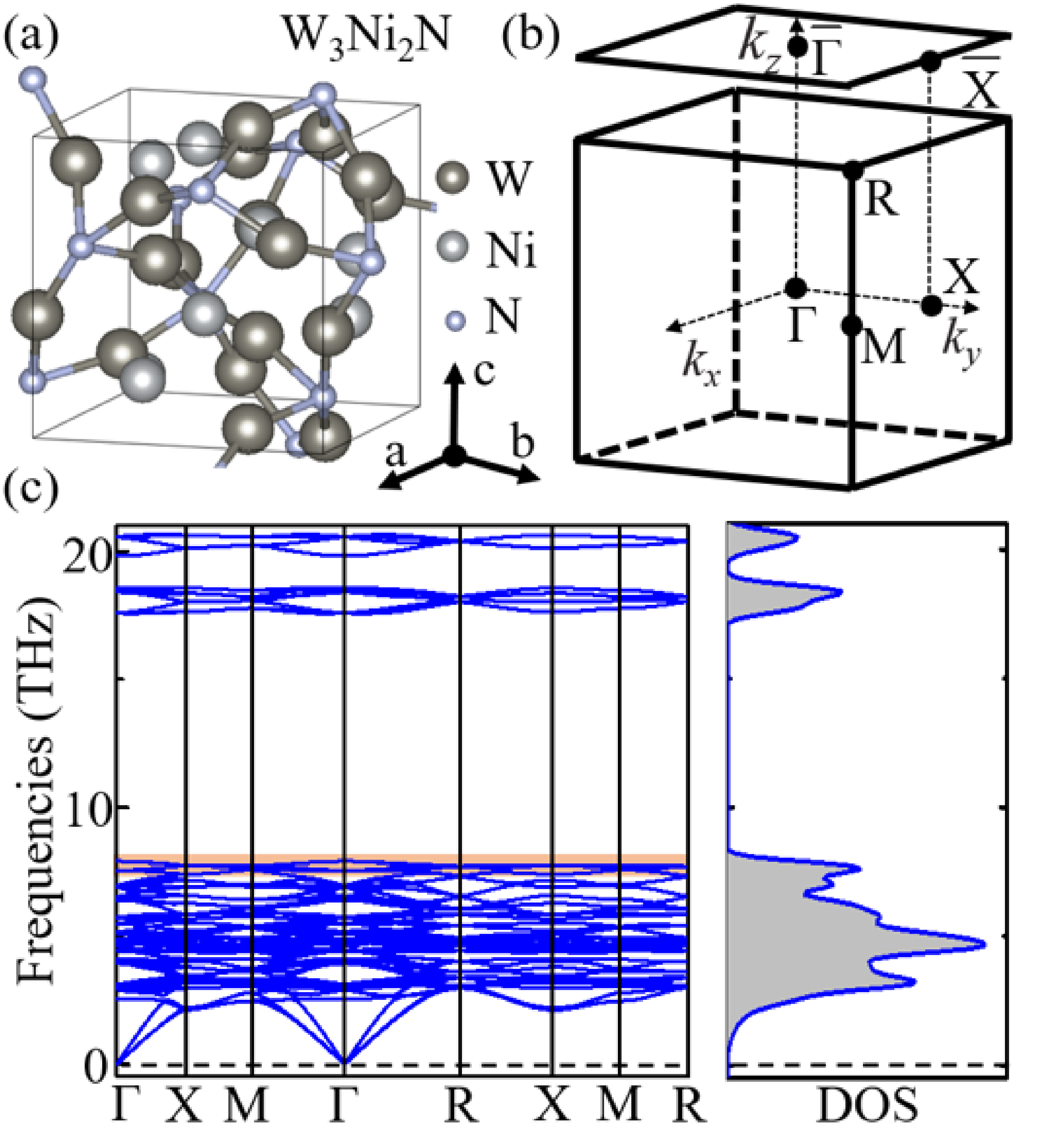

FIG. S46. A realistic candidate W_3Ni_2N in SG 213. (a) A unit cell contains 12 W, 8 Ni and 4 N atoms. (b) The first BZ of W_3Ni_2N. (c) The phononic dispersions along the high-symmetry directions and the phononic density of states (DOSs) of W_3Ni_2N. It is clearly seen that the a twofold IWP is localized at the high-symmetry point Γ (a red box), indicating the existence of IWP in this material.

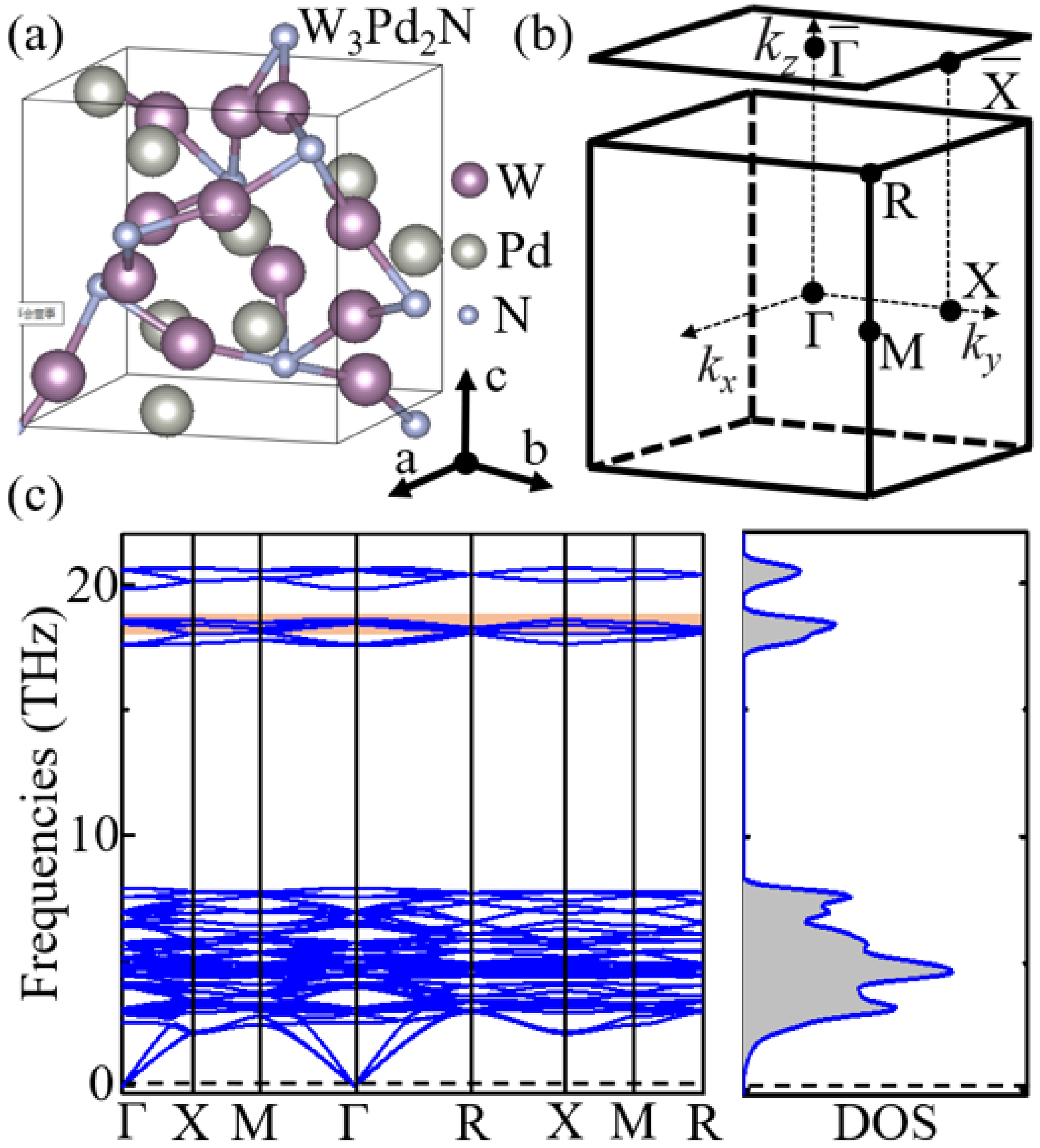

FIG. S47. A realistic candidate W_3Pd_2N in SG 213. (a) A unit cell contains 12 W, 8 Pd and 4 N atoms. (b) The first BZ of W_3Pd_2N. (c) The phononic dispersions along the high-symmetry directions and phononic density of states (DOSs) of W_3Pd_2N. It is clearly seen that the a twofold IWP is localized at the high-symmetry point Γ (a red box), indicating the existence of IWP in this material.

References

[1] H. Geoffroy, F. Chris, E. Virginie, J. Anubhav and C. Gerbrand, Data mined ionic substitutions for the discovery of new compounds, Inorg. Chem. **50**, 656 (2011).
[2] A. Togo and I. Tanaka, First principles phonon calculations in materials science, Scr. Mater. **108**, 1 (2015).
